# Sex steroids and steroidogenesis-related genes in the sea cucumber, *Holothuria scabra* and their potential role in gonad maturation

**DOI:** 10.1038/s41598-021-81917-x

**Published:** 2021-01-26

**Authors:** Tipsuda Thongbuakaew, Saowaros Suwansa-ard, Arada Chaiyamoon, Scott F. Cummins, Prasert Sobhon

**Affiliations:** 1 School of Medicine, Walailak University, Nakhon Si Thammarat, 80160 Thailand; 2 Genecology Research Centre, School of Science and Engineering, University of the Sunshine Coast, Sippy Downs, QLD 4556 Australia; 3 Department of Anatomy, Faculty of Medicine, Khon Kaen University, Khon Kaen, 40002 Thailand; 4 Department of Anatomy, Faculty of Science, Mahidol University, Bangkok, 10400 Thailand

**Keywords:** Transcriptomics, Animal physiology

## Abstract

The sea cucumber *Holothuria scabra* is an economically valuable marine species which is distributed throughout the Asia–Pacific region. With the natural population declining due to over fishing, aquaculture of this species is deemed necessary. Hence, it is essential to understand the mechanisms regulating the reproduction in order to increase their populations. Sex steroids, including estrogens, androgens and progestogens, play an important role in reproduction in most vertebrates and several invertebrates. It has been proposed that sea cucumbers have the same sex steroids as vertebrates but the steroidogenic pathway in the sea cucumbers is still unclear. In this study, we demonstrated by using liquid chromatography-tandem mass spectrometry (LC–MS/MS) that sex steroids (estradiol, progesterone, and testosterone) were present in *H. scabra* neural and gonadal tissues*. *In silico searches of available sea cucumber transcriptome data identified 26 steroidogenesis-related genes. Comparative analysis of encoded proteins for the steroidogenic acute regulatory protein (HscStAR), CYP P450 10, 17 and 3A (HscCYP10, HscCYP17, HscCYP3A) and hydroxysteroid dehydrogenases (Hsc3β-HSD, Hsc17β-HSD) with other species was performed to confirm their evolutionary conservation. Gene expression analyses revealed widespread tissue expression. Real-time PCR analysis revealed that *HscStAR*, *HscCYP10*, *Hsc3β-HSD,* and *Hsc17β-HSD* gene expressions were similar to those in ovaries and testes, which increased during the gonad maturation. *HscCYP17* mRNA was increased during ovarian development and its expression declined at late stages in females but continued high level in males. The expression of the *HscCYP3A* was high at the early stages of ovarian development, but not at other later stages in ovaries, however it remained low in testes. Moreover, a role for steroids in reproduction was confirmed following the effect of sex steroids on vitellogenin (Vtg) expression in ovary explant culture, showing upregulation of Vtg level. Collectively, this study has confirmed the existence of steroids in an echinoderm, as well as characterizing key genes associated with the steroidogenic pathway. We propose that sex steroids might also be associated with the reproduction of *H. scabra*, and the identification of biosynthetic genes enables future functional studies to be performed.

## Introduction

Sea cucumbers are one of the most important commercial sea cucumber species in the tropical Indo-Pacific countries. Being nutritious and a source of traditional medicine, *H. scabra* and other sea cucumbers have increased markedly in demand, particularly in the regions of Asia and the Middle East^1–3^. Sea cucumbers are considered nutritious due to their relatively high levels of proteins and low fat content, compared to most other foods^4,5^. They also contain valuable vitamins and minerals^2,6^. As a traditional medicine, sea cucumbers have been reported to contain biomolecules with positive pharmacological properties, including anti-angiogenic, anticancer, anticoagulant, anti-hypertension, anti-inflammatory, antimicrobial, antioxidant, antithrombotic, antitumor and wound healing bioactivity^2,6^.

*Holothuria scabra* is considered to be one of the most preferable sea cucumber species in the markets. The fishery of *H. scabra*, therefore, has grown rapidly^1,7^ and this has led to a depletion of species populations in the wild. Aquaculture of this species is, hence, needed for supporting a high demand as well as to repopulate this species in the wild. Nevertheless, their reproductive mechanism has not been well studied at the molecular level, yet knowledge gained could be applied aquaculture.

It is well known that steroid molecules have established roles as hormones in controlling reproduction in various animal groups such as gonadal maturation, germ cell proliferation, and sexual behavior in vertebrates^8,9^. Sex steroids (estrogens, androgens, and progestogens), commonly referred to vertebrate-type steroids, are also involved in regulation of reproductive processes in several groups of invertebrates^8–10^. To date, vertebrate-type steroids have been little studied in echinoderms besides the detection of vertebrate-type steroids in some species of starfish and sea urchin^11–13^. Moreover, the conversion of cholesterol into vertebrate-type steroids and reduced metabolites were also detected in the starfish and echinoid^14–19^. There is evidence that sex steroid hormones are also involved in regulation of reproductive processes in echinoderms^20–22^. Up to now, there is still no study of steroids or steroidogenesis in sea cucumbers. Furthermore, the identification of steroids in echinoderms has relied solely on immunoassays, which although quite accurate, cannot be held as definitive proof. Further proof often requires the isolation or characterization of the steroids using mass spectrometry or molecular identification of genes related to steroidogenesis. Recent genomic and transcriptomic information for sea cucumbers, including *H. scabra*, could help fill the gaps.

In this study, we first used mass spectrometry to confirm the presence of sex steroids in *H. scabra*. We subsequently identified steroidogenesis-related genes and defined their tissue expression patterns and changes during gonad maturation. The effect of steroids on vitellogenin (Vtg) expression in ovary explants was performed to confirm their role in oocyte development and reproduction.

## Methods

### Ethical statement

All the experimental procedures presented in this work were approved by the Animal Care and Use Committee of Walailak University, National Research Council of Thailand (NRCT), Protocol No. 008/2017. All protocols in this study were carried out in accordance with relevant guidelines and regulations for using animals, in compliance with ARRIVE guidelines.

### Steroid purification and liquid chromatograph-mass spectrometry (LC–MS/MS)

The protocols for steroid purification and LC–MS used in this study were based on Thongbuakaew et al.^23^. Briefly, the mature sea cucumbers *H. scabra* (n = 40) were obtained from Ko Libong, Trang Province, Thailand and anesthetized by immersion in ice-cold seawater for 30 min before sacrificed. Tissue samples [central nervous system (CNS); including combined radial nerve cords (RNC) and circumoral nerve ring (CNR), and gonads; including combined testes and ovaries] were collected and immediately frozen in liquid nitrogen and kept at – 80 °C until use. The tissues were homogenized separately in 3 ml of diethyl ether to extract the steroids. All samples were vortexed for 30 s then left for 5 min at 4 °C to allow steroids to separate. The supernatant (upper ether phase), containing steroids, was transferred to a fresh glass tube. The extraction was repeated three times for maximum extraction efficiency. Combined extracts were pooled and dried under a nitrogen stream at room temperature. For analysis, dried extracts were dissolved in 100 µl MeOH and then 10 µl of each sample was injected into the LC–MS/MS system for analysis.

LC–MS/MS analysis was performed using a Bruker Esquire HCT ion-trap mass spectrometer (Bruker Technologies, Bremen, Germany) equipped with an Agilent series 1100 LC system (Agilent Technologies, Waldbronn, Germany) and controlled by Bruker Daltonics DataAnalysis 3.4 (Bruker Technologies, Bremen, Germany). Chromatographic separation was achieved on a Thermo Scientific Hypersil GOLD aQ Polar Endcapped C18 column (2.1 mm × 150 mm, 5 µm) (Thermo Fisher Scientific, MA, USA). The column compartment was maintained at 40 °C with acetonitrile. The instrument was operated in both positive (progesterone and testosterone) and negative (estradiol) atmospheric pressure chemical ionization (APCI) and multiple reactions monitoring mode (MRM). A gradient elution program was conducted for chromatographic separation, with 0.1% formic acid in water as solvent A and acetonitrile as solvent B, and then pumped at a flow rate of 0.25 ml/min. The analytes were separated using the following gradient solvents: 0.0–1.0 min of 20–35% B; 1.0–20.0 min of 35–90% B; 20.0–25.0 min of 90% B; 25.0–25.1 min of 90–20% B; 25.1–30.0 min of 20% B. Drying gas flow and nebulizer pressure were set at 4 L/min and 30 psi. Drying gas temperature and capillary voltage of the system were adjusted at 350 °C and 4000 V, respectively. LC–MS/MS was performed using target ions at m/z 271 for estradiol^23,24^, m/z 315 for progesterone^23,25^, and m/z 289 for testosterone^26^, respectively.

### Sequence annotation, gene mining, and protein prediction

All relevant transcriptome data of *H. scabra* central nervous system (CNR and RNC) and gonads (testes and ovaries) were obtained from Suwansa-ard et al.^27^ reported on NCBI Sequence Read Archive (SRA) database under the accession number SRR5755244. Briefly, transcripts were selected and compared against the databases of NR, NT, Swiss-Prot, KEGG, COG, and GO, using BLAST and BLAST2GO software, with an E-value threshold of 1e−6. Relative abundance of all transcripts among different tissues was estimated by SOAP software version 2.21^28^. Transcripts encoding steroidogenesis-related genes were identified by tBLASTn searches against other known steroidogenesis-related genes, reported in previous studies using the CLC Main Workbench Version 7.7 (CLC Bio-Qaigen, AsiaPac, Taiwan). All hits were analyzed manually with their orthologous peptides from various species and the presence of conserved motifs. Analysis of protein similarity was performed by protein alignment using MUSCLE (http://www.ebi.ac.uk/Tools/msa/muscle/)^29^ and CLC Main Workbench Version 7.7. Prediction of conserved protein domains were performed by NCBI conserved domain database^30^ and InterPro (https://www.ebi.ac.uk/interpro)^31^. Protein precursors were analyzed for their evolutionary relationship with other known orthologous proteins, and illustrated by phylogenetic trees using the MEGA5 program using Neighbor-joining estimation (1000 bootstraps)^32^. Illustrations were indicated by the genus and species name.

### Tissue collection and total RNA extraction

Mature male (n = 10) and female (n = 10) sea cucumbers *H. scabra* were obtained from Ko Libong, Trang Province, Thailand, with an average weight of 100–150 g. They were then anesthetized by immersion in ice-cold seawater for 30 min before sacrifice. The various tissue samples including CNR, RNC, gonads (testes and ovaries), respiratory tree, longitudinal muscle, intestine, and body wall were collected and immediately frozen in liquid nitrogen and then kept at – 80 °C until preparation of total RNA. Frozen tissues were individually homogenized and the total RNA was extracted using the TRIzol reagent (Thermo Fisher Scientific, MA, USA), following the manufacturer’s protocol in combination with a DNase I (Thermo Fisher Scientific, MA, USA) treatment to eliminate potential genomic DNA contamination. The quantity and quality of RNA samples were measured using spectrophotometry (NanoDrop 1000; Thermo Fisher Scientific, DE, USA). Total RNA of each tissue was pooled and dried separately.

### Tissue distribution of steroidogenesis-related genes using reverse transcription polymerase chain reaction (RT-PCR)

Total RNA isolation was performed as described previously in “Tissue collection and total RNA extraction” section, then used for complementary DNA (cDNA) synthesis (RevertAid RT Reverse Transcription Kit; Thermo Scientific, USA). Gene-specific primers for target genes were designed using the Primer-BLAST program (https://www.ncbi.nlm.nih.gov/tools/primer-blast)^33^ (Table 1). PCR was carried out using the PCR SuperMix (Thermo Fisher Scientific, MA, USA) following a routine protocol optimized for the primers. As positive controls, the 16S rRNA gene was used, while the negative control was non-RT cDNA. PCR products were analyzed by agarose gel electrophoresis with ethidium bromide and amplicon sequences were confirmed by sequencing.

**Table 1:** Gene-specific primers of target genes involved in steroidogenesis, vitellogenin (Vtg), and 16S rRNA and of *H. scabra* and expected amplicon sizes.

Genes of interest	Forward primer (5′–3′)	Reverse primer (5′–3′)	Size (bp)
StAR	GTTGCAATCACCCCAGCAAG	GCTTTAGGCATCCACCCCTT	157
CYP10	ACGATCTGTTGCTTGGTGCT	TCTGGTGTTATCGGCGTTCC	145
CYP17	AGGATGAATGACCGTGGCAG	TCGCCCCAGAGTTTCTTGTC	197
CYP3A	TGTTCTGACTAACCGCTGCT	CTGTTTGGGTTGCTCTGGGA	165
3β-HSD	TGGGAGGGACTGGTTTTATTGG	GTGTTCTTCATTCATCCATGCCA	122
17β-HSD	GGTGCAGTTCATCTTACGCA	CTGCAACACCATCTACAGCAC	151
Vtg	TGAACACGTCATGGTACGCT	CAGTTCATCGGCCCTGACAA	139
16S rRNA	AAGCTGACCTGACTTGCGTC	ACGAGAAGGTTTGCGACCTC	130

### Quantitative real-time PCR of steroidogenesis-related genes

Quantitative real-time PCR (qRT-PCR) was used to study the changes in mRNA levels of target genes of interest in *H. scabra* during gonad maturation. Gonad maturation was divided into 5 stages based on the classification by Rasolofonirina et al.^34^. Total RNA and cDNA of testes (n = 10/stage) and ovaries (n = 10/stage) were prepared as described previously in “Tissue collection and total RNA extraction” and Tissue distribution of steroidogenesis-related genes using reverse transcription polymerase chain reaction (RT-PCR) sections. All gene-specific primers for the analysis are shown in Table 1. Expression of 16S rRNA was used as an internal control. The qRT-PCR was performed using the GeneRead qPCR SYBR Green Mastermix (Qiagen, Hilden, Germany) following the manufacturer’s protocol. Thermocycling conditions were 95 °C for 5 min and 40 cycles of 95 °C for 30 s, and 60 °C for 30 s and 72 °C for 30 s. Dissociation curve analysis was also included; one cycle of 95 °C for 1 min, 60 °C for 30 s, and 95 °C for 30 s. The transcripts were quantified using a standard curve method^35^. Standard curves for selected genes of interest and 16S rRNA were generated by tenfold serial dilutions of known concentrations of the plasmids containing the target transcripts. The detection range, linearity, and real-time PCR amplification efficiency of each primer pair were checked before continuing with sample analysis. The qRT-PCR reaction efficiency was calculated from the standard curve, which ranged from 90 to 100%. Expression of 16S rRNA was verified before continuing used as the internal reference to correct for differences in reverse transcription efficiency and template quantity. All standards and experimental samples were run in duplicate. The amounts of target and internal reference in experimental samples were determined from the respective standard curves using Rotor-Gene 6000 Series Software 1.7. Transcript levels of selected genes of interest were normalized to the level of 16S rRNA and the data will be expressed as relative mRNA levels. The data of each group was expressed as a mean ± SD and performed with a SPSS program using a one-way analysis of variance (ANOVA), followed by a Tukey’s post hoc multiple comparison. The probability value less than 0.05 (p < 0.05) indicated a significant difference.

### Effect of steroids on vitellogenin (Vtg) expression in ovary explants

The protocol for ovarian tissue culture used in this study was based on Merlin et al.^36^ and Thongbuakaew et al.^37^. Briefly, fragments of stage 3 ovaries, a late developing stage (0.2–0.3 g) based on the classification by Rasolofonirina et al.^34^ obtained from female *H. scabra* (n = 3) were dissected and washed in Leibovitz's L-15 medium (Gibco, Grand Island, NY, USA) containing 1000 IU/ml penicillin and 1000 μg/ml streptomycin. These samples were then cultured in 24 well culture plates (Falcon 35-3078, Beckton Dickinson, Franklin Lakes, NJ, USA) containing 2 mL Leibovitz's L-15 medium (Gibco, Grand Island, NY, USA) containing 100 μg/ml of streptomycin, 100 IU/ml of penicillin. The concentration used in this study was based on screening tests of efficacy concentration. For screening tests, estradiol and progesterone were added into the media containing ovaries to obtain final concentrations of 10^–3^, 10^–4^, and 10^–5^ M, respectively. Among three doses of estradiol and progesterone, it appeared that 10^–5^ M was the most effective concentration for stimulation of *Vtg* expression. The ovarian explant cultures were then treated with 10^–5^ M estradiol and progesterone at 0 min. Physiological saline was added to negative control groups instead of estradiol and progesterone. All plates were incubated with gentle shaking in the dark at 25 °C for 30, 60, 120 and 180 min, and experiments were performed in duplicate. At each time-point, the ovarian fragments were collected and immediately frozen in liquid nitrogen, then stored at − 80 °C until total RNA extraction. Total RNA was extracted as described previously in “Tissue collection and total RNA extraction” section. The expression level of Vtg were measured using quantitative real-time PCR with gene-specific primers shown in Table 1, as described previously in “Quantitative real-time PCR of steroidogenesis-related genes” section. The data of each group was expressed as a mean ± SD and performed with a SPSS program using a one-way analysis of variance (ANOVA), followed by a Tukey’s post hoc multiple comparison. The probability value less than 0.05 (p < 0.05) indicated a significant difference.

## Results

### Identification of sex steroids in *H. scabra*

**Figure 1:**
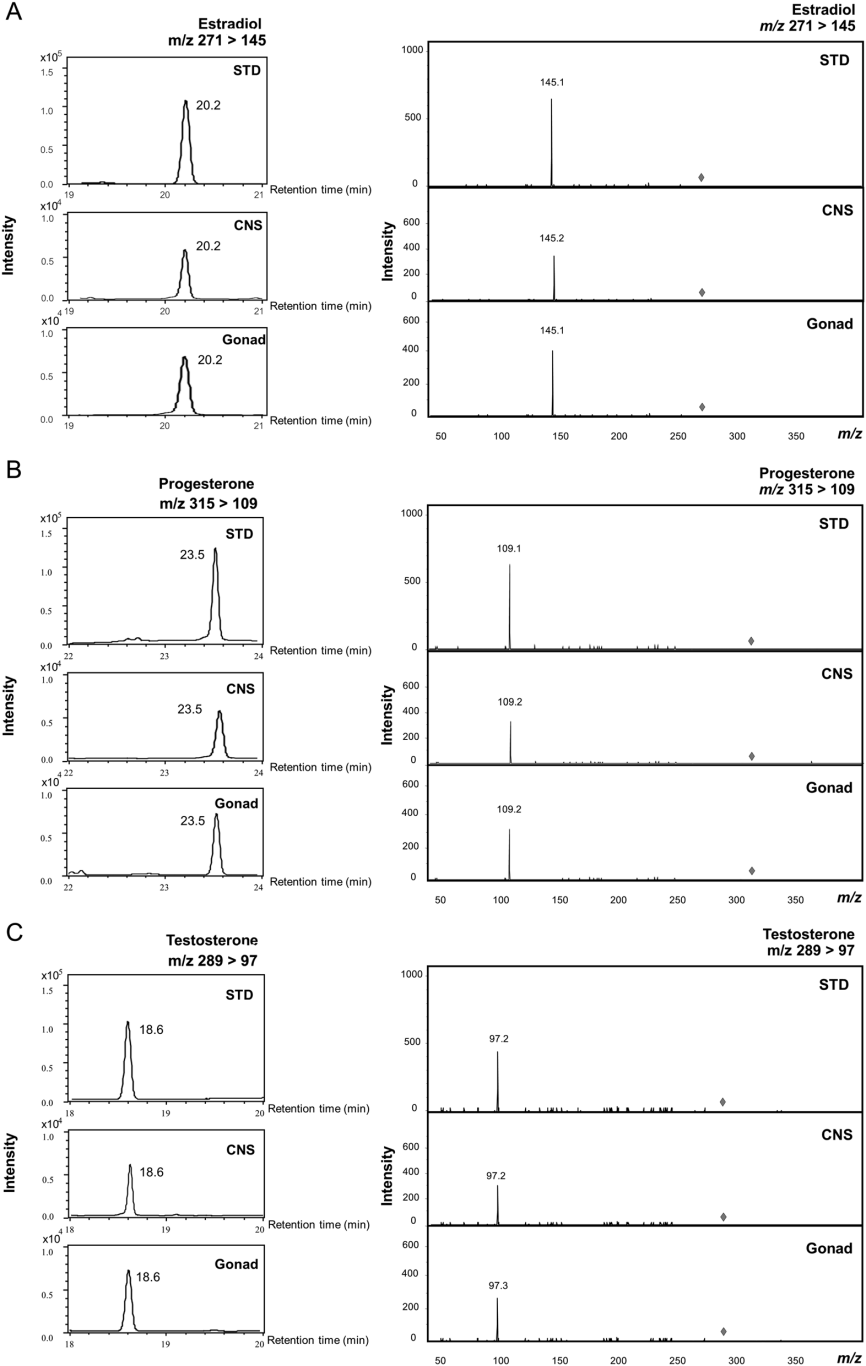
Chromatograms and mass spectra corresponding to the LC–MS/MS analysis of estradiol, progesterone, and testosterone isolated from CNS and gonad tissue of *H. scabra.* (**A**) Representative chromatogram and MRM spectra for estradiol transitions m/z 271 → 145. (**B**) Representative chromatogram and MRM spectra for progesterone transitions m/z 315 → 109. (**C**) Representative chromatogram and MRM spectra for testosterone transitions m/z 289 → 97. STD; standard, CNS; central nervous system.

A representative chromatogram of the MRM transition of the analytes isolated from CNS and gonad tissue of *H. scabra* by comparing with a standard is shown in Fig. 1. The base peak selected for quantification of estradiol (m/z = 271) corresponded to the deprotonated molecule [M–H]^−^. The major product ions corresponded to the A–B ring moiety (m/z = 145) (Fig. 1A). On the other hand, progesterone was detected as protonated molecules [M+H]^+^ m/z = 315. The main product ions were m/z = 109 deriving from the cleavage of the B- and C-ring (Fig. 1B). Testosterone (m/z = 289) was also detected as protonated molecules [M+ H]^+^. In MS/MS spectra, the main product ions corresponded to the cleavage of the B- and C-ring (m/z = 97) (Fig. 1C).

### Identification of steroidogenesis-related genes

**Figure 2:**
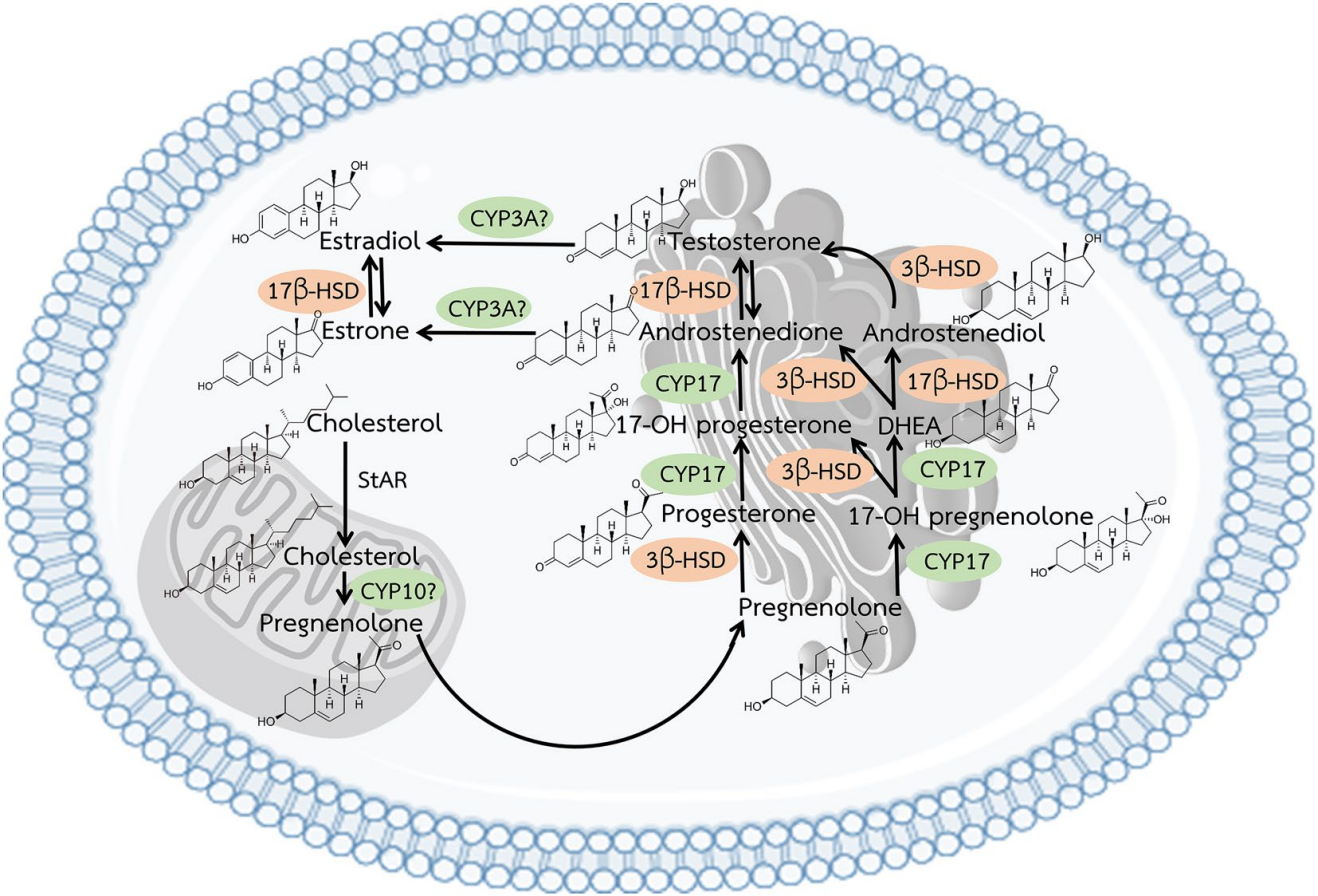
Schematic diagram showing the putative pathway for steroidogenesis in the *H. scabra* between mitochondria and the smooth endoplasmic reticulum (SER)*.* In the pathway of steroid hormone biosynthesis, there are two major types of enzymes: cytochromes P450 (green) and other hydroxysteroid dehydrogenases (orange). In *H. scabra*, we identified gene transcripts encoding steroidogenic acute regulatory protein (StAR), cytochrome P450 10 (CYP10), 17alpha-hydroxylase/17, 20-lyase (CYP17), cytochrome P450 3A (CYP3A), 3beta-hydroxysteroid dehydrogenase (3β-HSD), and 17beta-hydroxysteroid dehydrogenase (17β-HSD). Question marks remain unclear. Steroidogenesis begins in the cells where cholesterol transport into the mitochondria facilitated by StAR. Once in the mitochondria, cholesterol may be converted to pregnenolone through the action of CYP10. Pregnenolone then diffuses to the SER, where it is further converted into a series of reactions to testosterone. Testosterone may be subsequently aromatized to estradiol through the action of CYP3A.

We used a de novo assembled transcriptome for *H. scabra* to identify steroidogenic-related genes. We found 26 transcripts encoding steroidogenesis-related genes, including members of the steroidogenic activator, steroid hormone receptor, and various steroidogenic enzymes. Sequence and annotation information is provided in Table 2 and S1. Moreover, Fig. 2 illustrates a proposed biosynthesis pathway that includes those enzymes involved in the production of the active steroid hormones, progesterone, estradiol and testosterone. The cholesterol side-chain cleavage performed by CYP11 may be replaced by CYP10 in this sea cucumber, however, this remains unclear. Similarly, the aromatase (CYP19) may be replaced by CYP3A, which converts androgens to estrogens in some species, but has not been documented in the sea cucumber.

**Table 2:** Summary, including BLAST annotation, of genes found in *H. scabra* involved in the steroidogenesis pathway.

Steroidogenic-related genes	Transcripts	Length (aa)	BLAST hit and species	E-value	Accession numbers
**Steroidogenic activator**					
Steroidogenic acute regulatory protein (StAR)	Contig_2100	F/381	Steroidogenic acute regulatory protein, mitochondrial-like [*Strongylocentrotus purpuratus*]	7.00E−69	XP_011660912.1
StAR-related lipid transfer protein	Contig_1443	P/184	stAR-related lipid transfer protein [*Strongylocentrotus purpuratus*]	2.00E−91	XP_789877.3
Steroid receptor RNA activator 1	Contig_22299	F/238	Steroid receptor RNA activator 1 [*Orycteropus afer afer*]	1.00E−24	XP_007937187.1
Androgen-induced gene 1 protein	Contig_55776	P/233	Androgen-induced gene 1 protein isoform X1 [*Strongylocentrotus purpuratus*]	2.00E−64	XP_793283.4
**Steroid hormone receptor**					
Steroid hormone receptor 3	Contig_4954	P/547	Steroid hormone receptor 3 [*Strongylocentrotus purpuratus*]	0.00E+00	NP_001020384.1
Steroid hormone receptor spshr2	Contig_61406	P/135	Steroid hormone receptor spshr2 [*Strongylocentrotus purpuratus*]	2.00E−57	NP_001116968.1
Orphan steroid hormone receptor 2-like	Contig_15452	P/316	Orphan steroid hormone receptor 2-like [*Saccoglossus kowalevskii*]	6.00E−122	XP_002739506.2
Membrane-associated progesterone receptor component	Contig_13885	F/175	Membrane-associated progesterone receptor component 1 [*Strongylocentrotus purpuratus*]	7.00E−75	XP_783332.1
Estrogen-related receptor-like (ERR)	Contig_41919	P/205	Steroid hormone receptor ERR2-like [*Lingula anatina*]	3.00E−41	XP_013405267.1
**Steroidogenic enzymes**					
17-Beta-hydroxysteroid dehydrogenase type 1	Contig_13797	P/212	Estradiol 17-beta-dehydrogenase 1 [*Miniopterus natalensis*]	6.00E−31	XP_016057672.1
17-Beta-hydroxysteroid dehydrogenase type 2	Contig_63395	P/170	Estradiol 17-beta-dehydrogenase 2-like [*Elephantulus edwardii*]	1.00E−41	XP_006888866.1
17-Beta-hydroxysteroid dehydrogenase type 4	Contig_19638	F/738	17 Beta hydroxysteroid dehydrogenase 4 [*Salmo trutta fario*]	0.00E+00	ACN66287.1
17-Beta-hydroxysteroid dehydrogenase type 12	Contig_36373, 34365	F/331	Estradiol 17-beta-dehydrogenase 12 [*Saccoglossus kowalevskii*]	9.00E−54	XP_002733503.1
17-Beta-hydroxysteroid dehydrogenase type 13	Contig_27928	P/280	17-Beta-hydroxysteroid dehydrogenase 13-like [*Saccoglossus kowalevskii*]	3.00E−85	XP_002732320.1
17-Beta-hydroxysteroid dehydrogenase type 14	Contig_13478	P/162	17-Beta-hydroxysteroid dehydrogenase 14-like [*Plutella xylostella*]	3.00E−70	XP_011558998.1
3-Beta-hydroxysteroid dehydrogenase	Contig_33425	P/54	3-Beta-hydroxysteroid dehydrogenase, putative [*Ixodes scapularis*]	6.00E−13	XP_002407062.1
Steroid 17-alpha-hydroxylase/17,20 lyase	Contig_19734	P/379	Steroid 17-alpha-hydroxylase/17,20 lyase [*Strongylocentrotus purpuratus*]	1.00E−117	XP_789963.1
Inactive hydroxysteroid dehydrogenase-like protein	Contig_48833	P/108	Inactive hydroxysteroid dehydrogenase-like protein 1-like [*Saccoglossus kowalevskii*]	8.00E−39	XP_002733500.1
Hydroxysteroid dehydrogenase protein 2	Contig_6984	F/415	Hydroxysteroid dehydrogenase protein 2 [*Daphnia magna*]	3.00E−165	KZS05818.1
Estrogen sulfotransferase type 1	Contig_63795	P/93	Estrogen sulfotransferase [*Strongylocentrotus purpuratus*]	5.00E−11	XP_003726411.2
Estrogen sulfotransferase type 2	Contig_71705	P/88	Estrogen sulfotransferase-like [*Strongylocentrotus purpuratus*]	2.00E−21	XP_793921.2
NAD(P) dependent steroid dehydrogenase-like	Contig_64252	P/136	NAD(P) dependent steroid dehydrogenase-like [*Apostichopus japonicus*]	2.00E−69	PIK44317.1
Cytochrome P450 4V2 isoform 1	Contig_68628	P/216	Cytochrome P450 4V2 [*Strongylocentrotus purpuratus*]	7.00E−86	XP_799260.2
**Steroidogenic enzymes**					
Cytochrome P450 4V2 isoform 2	Contig_41285	P/202	Putative cytochrome P450 4V2 [*Apostichopus japonicus*]	8.00E−97	PIK50796.1
Cytochrome P450 10	Contig_59906, 27680	P/364	Predicted: cytochrome P450 10 [*Strongylocentrotus purpuratus*]	7.00E−67	XP_003727902.1
Cytochrome P450 3A24	Contig_4256	P/464	Putative cytochrome P450 3A24 [*Apostichopus japonicus*]	0.00E+00	PIK42887.1

P; Partial sequence, F; Full-length sequence. Amino acid sequences are shown in S1.

### Characterization of target genes involved in steroidogenesis

#### Steroidogenic acute regulatory protein (StAR)

**Figure 3:**
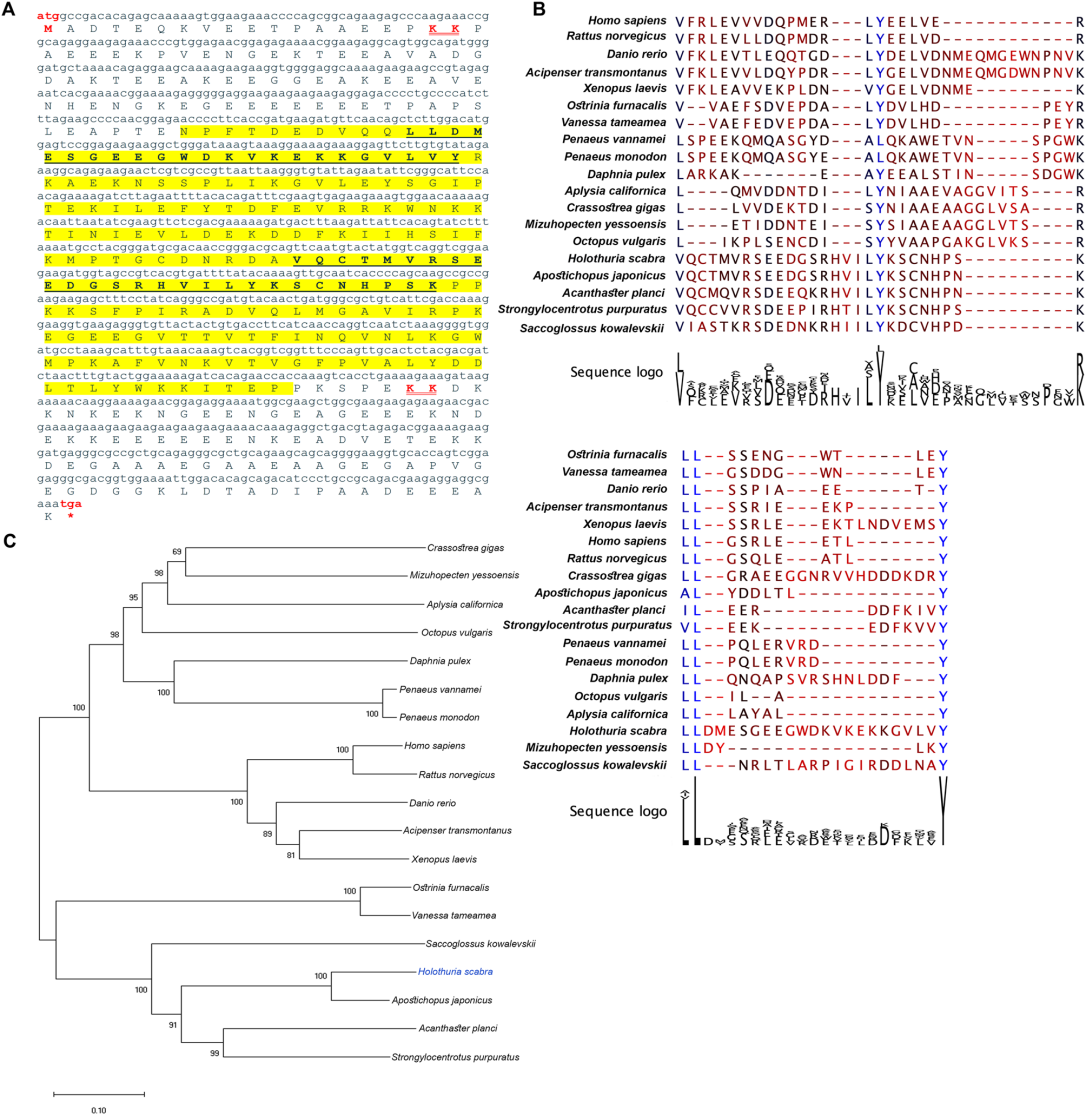
Characterization and phylogenetic tree analysis of *H. scabra* steroidogenic acute regulatory protein (StAR). (**A**) HscStAR contains the steroidogenic acute regulatory protein-related lipid transfer domain (START domain) (yellow highlight) which consists of cholesterol recognition/interaction amino acid consensus (CRAC) (L/V-(x)-Y-(x)-R/K) and dileucine- and tyrosine- (LL-(x)-Y) motifs (bold underlined letters), cleavage site (red double-underlined letters), start and stop codons (red letters). (**B**) Multiple sequence alignment of CRAC and LL-(x)-Y key motifs of StAR displays conservation among species. Sequence alignment represented by * = identical, : = strong homology, and . = less homology. (**C**) Phylogenetic tree of HscStAR constructed based on Neighbour-joining analysis with 1000 replicates bootstrap. Scale bar represents amino acid differences. Amino acid sequences and their accession number are shown in S2.

The *HscStAR* transcript encodes a full-length protein composed of 381 amino acids, which contains a steroidogenic acute regulatory protein-related lipid transfer domain (START domain) at positions 192–218 (Fig. 3A). The START domain has the cholesterol recognition/interaction amino acid consensus (L/V-(x)-Y-(x)-R/K) which is important for recognizing cholesterol and interacting with the cholesterol-rich domains of cell membranes. HscStAR also has dileucine- and tyrosine- motifs (LL-(x)-Y) at positions 97–98 and 119 that are critical for the targeting or the proper protein folding^38^. Alignment of HscStAR with other species homologs demonstrates conservation within these key motifs (Fig. 3B). Phylogenetic analysis shows that HscStAR clusters with homologs of echinoderms, hemichordate, cnidarians, and insects and is clearly distinguished from the vertebrates, crustaceans, and molluscs (Fig. 3C).

#### Cytochrome P450s (CYPP450s)

**Figure 4:**
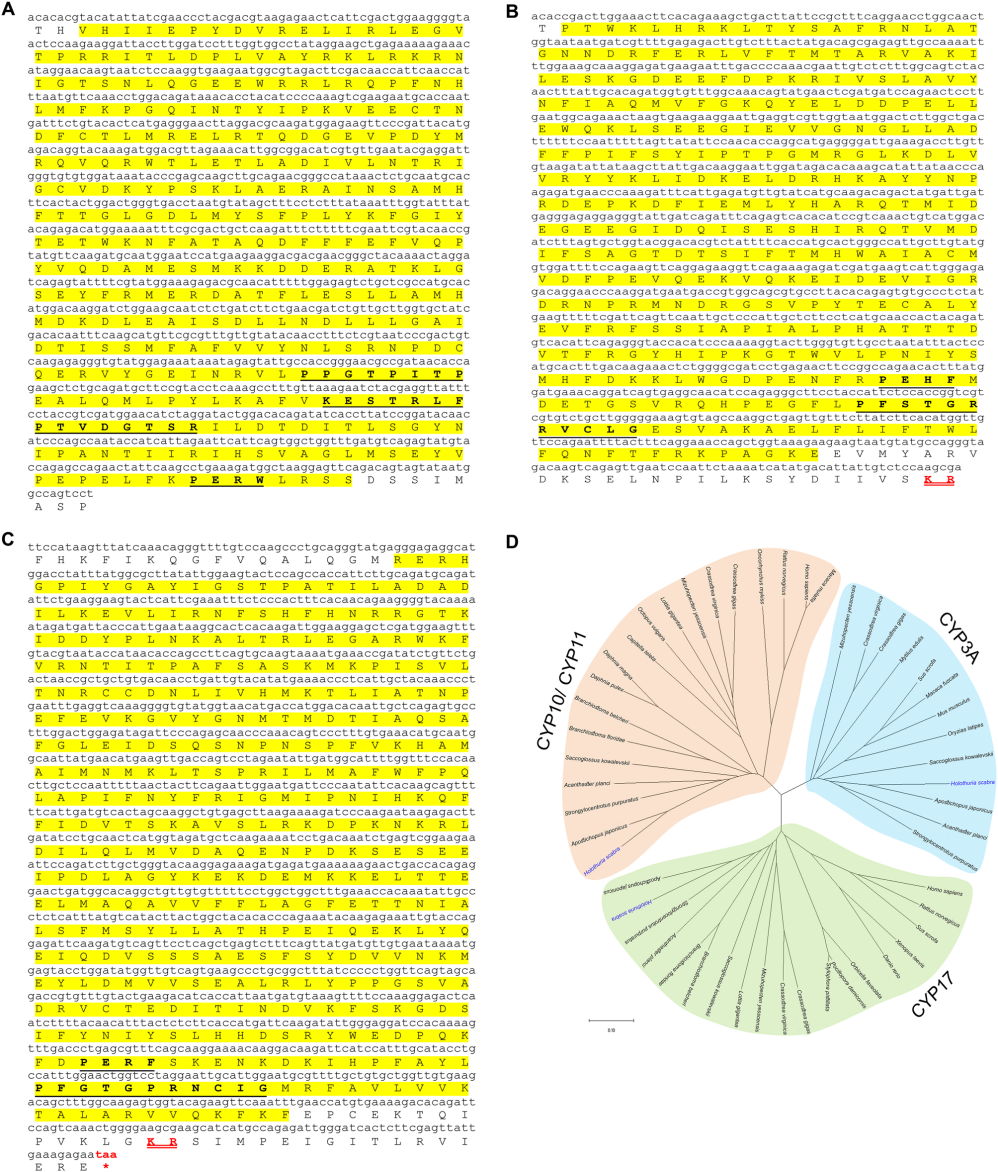
Characterization and phylogenetic tree analysis of *H. scabra* cytochrome P450 genes associated with steroidogenesis. (**A**) HscCYP10 contains the cytochrome P450 domain (yellow highlight) that includes a proline-rich motif (PPGTPITP), PERF/W, and KET/S(x)R(x)P(x)R regions (bold underlined letters). (**B**) HscCYP17 showed cytochrome P450 domain (yellow highlight) contains the heme-binding region (PFSTGRRVCLG) and PEHF region (bold underlined letters), and cleavage site (red double-underlined letters). (**C**) HscCYP3A contains cytochrome P450 domain (yellow highlight), which consists of PERF and heme-binding (PFGTGPRNCIG) regions (bold underlined letters), cleavage site (red double-underlined letters), and stop codons (red letters). (**D**) Phylogenetic tree of target CYP 450 involved in steroidogenesis constructed based on Neighbour-joining analysis with 1000 replicates bootstrap. Scale bar represents amino acid differences. Amino acid sequences and their accession number are shown in S2.

The well-known steroidogenesis CYP, CYP11 (or P450 side-chain cleavage enzyme), was not identified in *H. scabra*; however we identified a partial transcript that encodes the related HscCYP10 precursor. HscCYP10 contains the cytochrome P450 domain, as well as a proline-rich motif (PPGTPITP) and PERF/W motif, which is involved in heme incorporation and/or stability/catalytic activity of cytochrome P450^39,40^ (Fig. 4A). CYP10 and CYP11 sequences are conserved within the PERF/W motif; however, proline-rich motif was conserved within the CYP10 of echinoderms and hemichordate. Interestingly, we found that CYP10 and CYP11 sequences showed high similarity in the KET/S(x)R(x)P(x)R region (Fig. S1A). Phylogenetic analysis within the invertebrate CYPs shows that CYP10 and CYP11 are located in the same clades and share a relatively close evolutionary origin with CYP17 and CYP3A (Fig. 4D).

In *H. scabra*, a transcript was identified that encodes a partial CYP17 (17-alpha-hydroxylase/17, 20 lyase) precursor which has a cytochrome P450 domain containing the heme-binding region (PFSTGRRVCLG) and PEHF region (Fig. 4B). The heme-binding region (PFxxGxxxCxG) of CYP17 shows high similarity in all species, which contain the highly conserved cysteine residue that holds the heme-group in place^40^ (Fig. S1B). Notably, the PERF/W motif of CYP17 is variable in different species but still contains the highly conserved PxxF motif, which plays a role in heme pocket locking and core structure stabilizing^40^ (Fig. S1B). The HscCYP17 forms a phylogenetic clade with echinoderm homologs and is a sister clade with cephalochordate and mollusc homologs (Fig. 4D). Moreover, it was clearly distinguished from vertebrates and other invertebrate CYP17 and with evolutionary relatedness to CYP10/CYP11 and CYP3A. We also identified a transcript that encodes a partial HscCYP3A precursor, which recently may replace the CYP19 (also known as aromatase) in invertebrates. HscCYP3A precursor includes a cytochrome P450 domain with PERF and heme-binding (PFGTGPRNCIG) regions (Fig. 4C). HscCYP3A clusters within other echinoderms and hemichordates (Fig. 4D) and shares a common evolutionary origin with CYP10/CYP11 and CYP17.

#### Hydroxysteroid dehydrogenases (HSDs)

**Figure 5:**
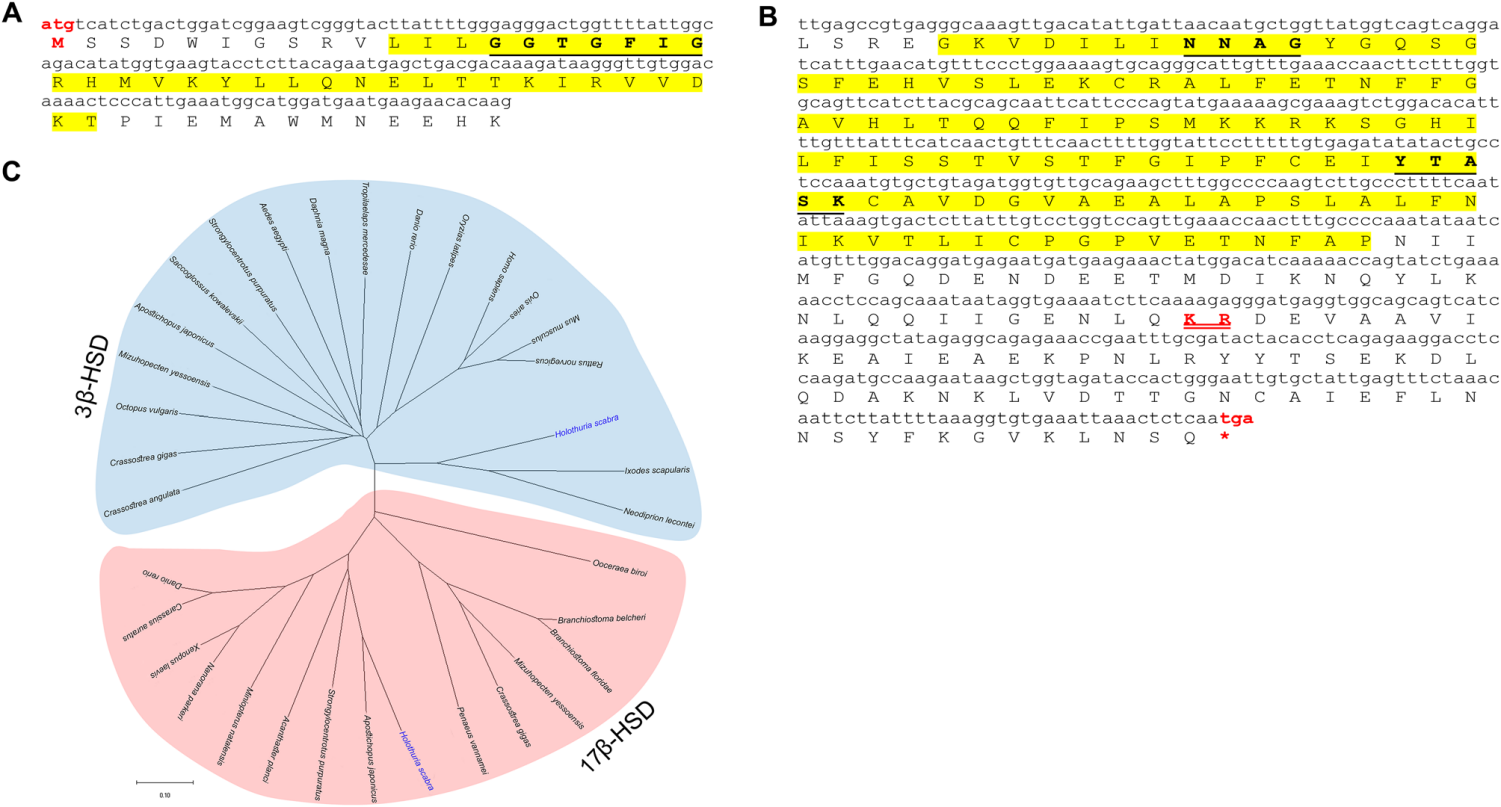
Characterization and phylogenetic tree analysis of *H. scabra* hydroxysteroid dehydrogenases (HSDs). (**A**) Hsc3β-HSD shows the start codons (red letters) and short-chain dehydrogenase/reductase (SDR) domain (yellow highlight) which contains NAD-binding motif (GGTGFIG) (bold underlined letters) (**A**). Shaded regions indicate motif sequences of amino acids identical to NAD-binding motif. (**B**) Hsc17β-HSD contains the short-chain dehydrogenase/reductase (SDR) domain (yellow highlight) that composed of 2 important motifs; NNAG and YxxxK (bold underlined letters), cleavage site (red double-underlined letters), and stop codons (red letters). (**C**) Phylogenetic tree of target HSDs involved in steroidogenesis constructed based on Neighbour-joining analysis with 1000 replicates bootstrap. Scale bar represents amino acid differences. Amino acid sequences and their accession number are shown in S2.

A *Hsc3β-HSD* gene was identified that encodes a partial precursor with a 3β-HSD domain containing a nicotinamide adenine dinucleotide (NAD)-binding motif (GGTGFIG). This amino acid motif is a fingerprint for the short-chain dehydrogenase/reductase (SDR) superfamily that binds with NAD, nicotinamide adenine dinucleotide phosphate (NADP) and related cofactors^10,23^ (Fig. 5A). *17β-HSD* transcripts were also identified in *H. scabra*. The partial Hsc17β-HSD precursor contains an SDR domain (Fig. 5B). The Hsc17β-HSD is composed of 2 important motifs; NNAG and YxxxK that act as a cofactor-binding and catalytic triad, where each of the residues is highly conserved^10,23^ (Fig. S2B). Multiple sequence alignment of the Hsc3β-HSD with the other species homologs shows little similarity besides the NAD-binding motif (GxxGxxG) (Fig. S2A), although phylogenetic tree analysis shows that Hsc3β-HSD clusters closely with crustaceans and is evolutionary related to Hsc17β-HSD (Fig. 5C). The Hsc17β-HSD is phylogenetically positioned within the clade of other echinoderms that is closest to the vertebrate group, thus is clearly distinguishable from other invertebrate species.

### Tissue-specific and temporal expression of target genes involved in steroidogenesis

**Figure 6:**
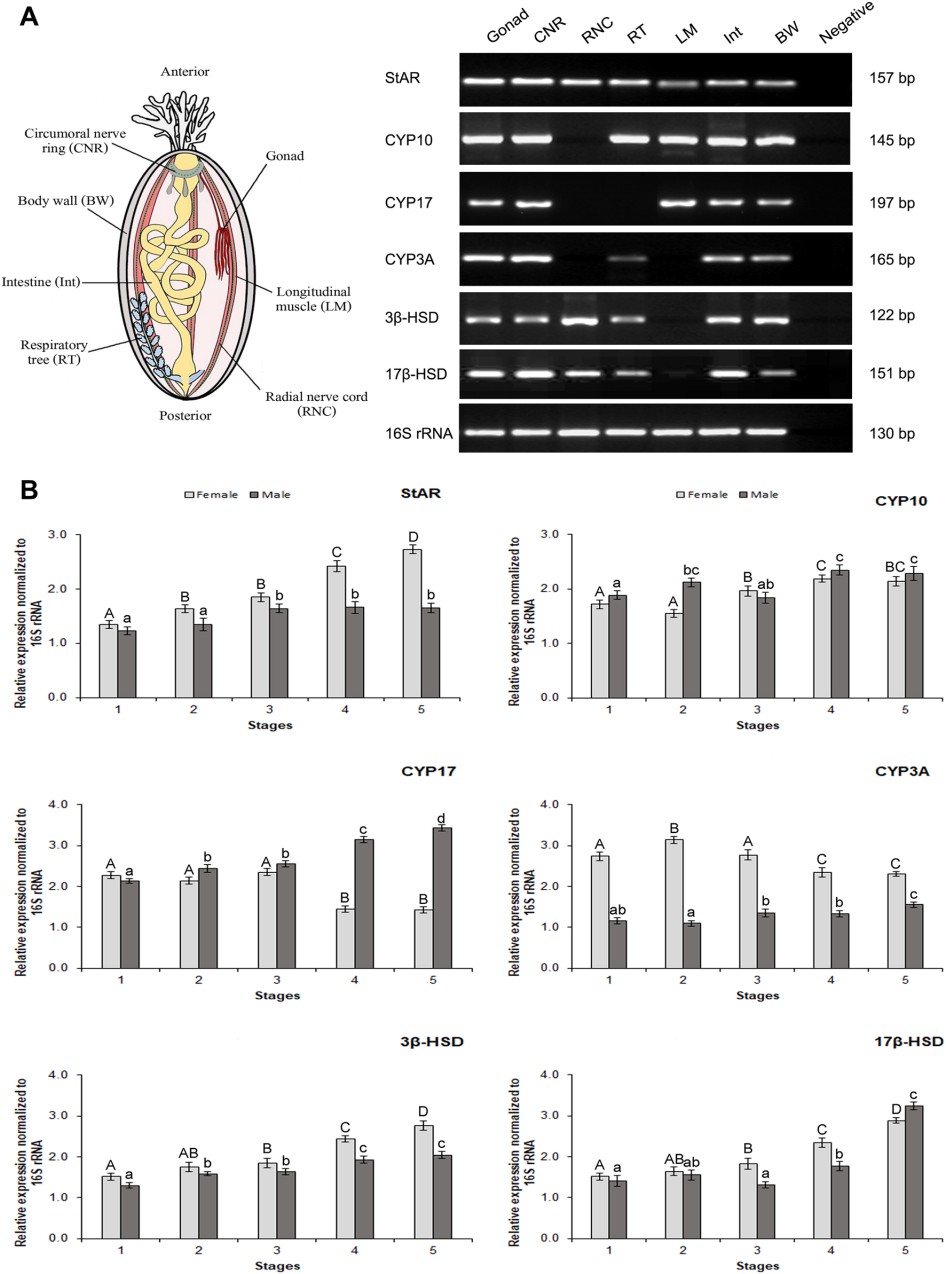
Tissue distribution and quantitative analysis of target genes involved in steroidogenesis in *H. scabra.* (**A**) Left—Schematic of sea cucumber showing tissues analysed by RT-PCR. Right—Agarose gel showing amplicons for StAR; steroidogenic acute regulatory protein, CYP10; cytochrome P450 10, CYP17; 17α-hydroxylase/17,20-lyase, CYP3A; cytochrome P450 3A, 3β-HSD; 3β-hydroxysteroid dehydrogenase, 17β-HSD; 17β-hydroxysteroid dehydrogenase, 16S rRNA; internal control, and negative; no RT-template. (**B**) Quantitative expression profiles in gonadal tissues of *H. scabra* during reproductive maturation (stages 1–5) (n = 10 for each sex at each stage). Data were normalized against 16S rRNA and the relative expression levels represented by the mean ± SD. Different letters indicate significant difference (p < 0.05) among reproductive stages of females (capital letters) and males (small letters) *H. scabra*.

RT-PCR was performed in order to determine the tissue-specific expression of *HscStAR*, *CYP* and *HSD* genes in *H. scabra* tissues (Fig. 6A). Results show that all genes expressed in the gonad, CNR, intestine and body wall. All CYPs are absent from the RNC, while *HscCYP17* and *HscCYP3A* are absent from the respiratory tree and longitudinal muscle, respectively. Both *HSD* genes are also not expressed within the longitudinal muscle. We further analyzed the relative gene expression in the gonadal tissues during reproductive maturation by qRT-PCR (Fig. 6B). Results revealed that the expression of *HscStAR*, *HscCYP10*, and *Hsc3β-HSD* slightly increased during the reproductive development in both ovaries and testes. In the ovaries, high expression of *HscCYP17* was detected at the early stage and declined at later stages of the reproductive cycle, whereas *HscCYP17* was upregulated during reproductive phase in testes. The expression of the *HscCYP3A* was high at the early stages of ovarian development, but not at other later stages in ovaries, however, *HscCYP3A* continued low in testes. *Hsc17β-HSD* gene expressions were similar to those in ovaries and testes, which increased as gonads matured during the reproductive cycle. Moreover, *Hsc17β-HSD* shows the highest degree of expression at stage 5 of the testes.

### Effect of steroids on vitellogenin (Vtg) genes expression

**Figure 7:**
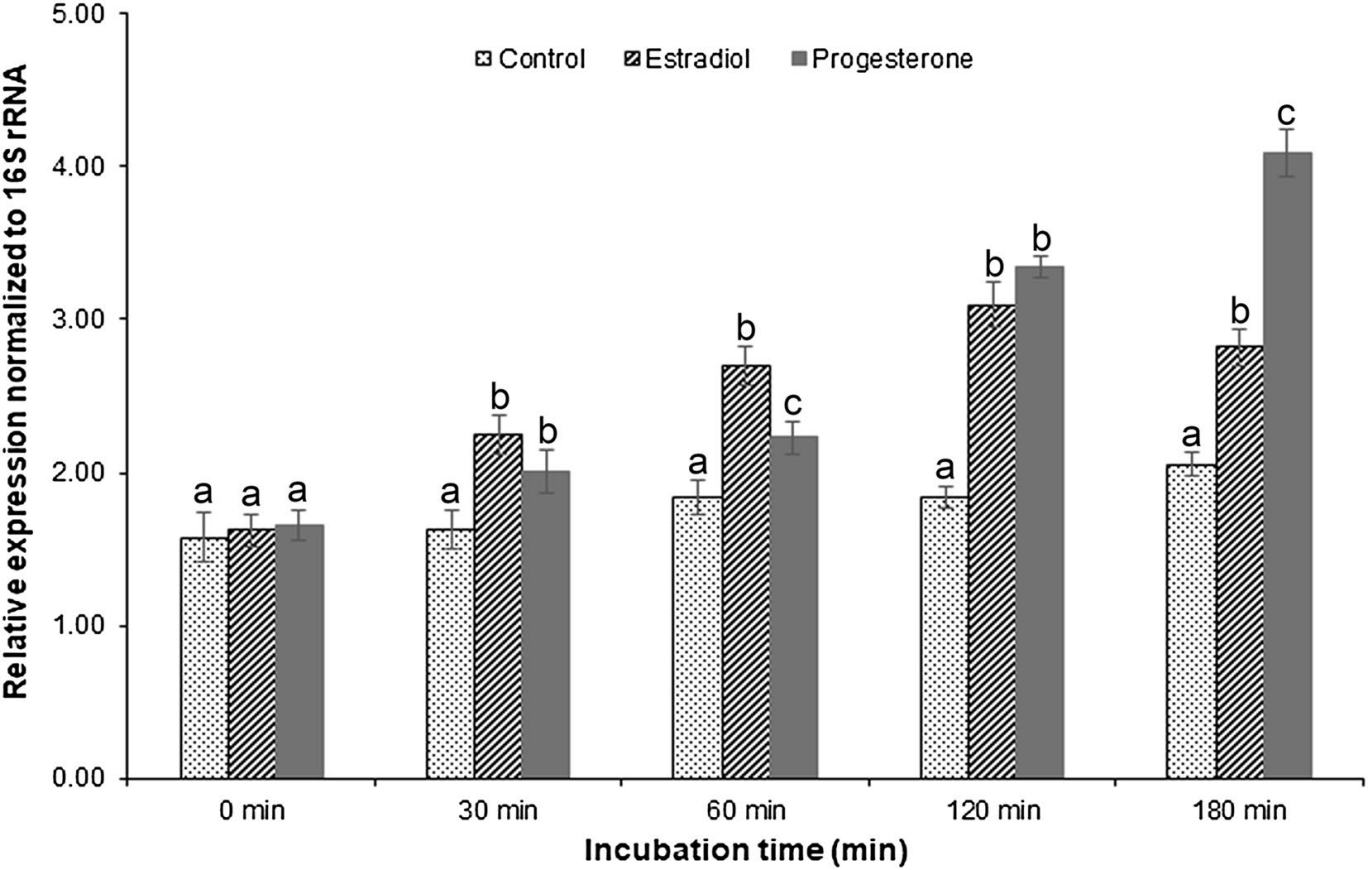
Effect of estradiol and progesterone on *Vtg* expression in ovarian tissue explants between 0 and 180 min. Relative gene expression levels of *Vtg* shown by quantitative real time-PCR (n = 3). Data were normalized against 16S rRNA and represented as mean ± S.D. Different letters indicate statistically significant different (p < 0.05) among the groups at the same incubation time.

Alqaisi et al. reported that echinoderms produce Vtg, which contribute to the yolk protein in mature eggs^41^. The developing stages (stages 2–3) of the reproductive cycle refer to the most active oocyte growth period that further develops to mature stages. Next, we investigated whether 10^–5^ M estradiol and progesterone could affect *Vtg* gene expression in stage 3 ovary explants. Results showed that estradiol and progesterone could significantly upregulate the expression of *Vtg* in ovarian explants at all incubation time points, compared with the control (Fig. 7). Moreover, incubated ovarian explants with estradiol and progesterone gradually increased *Vtg* gene expression compared with the control (Fig. 7). The expression of *Vtg* was higher in the estradiol treated group at the beginning of the incubation time (at 30 min and 60 min). During incubation for 120 min and 180 min, a large increase in mRNA levels of Vtg was detected in the progesterone treated group (Fig. 7).

## Discussion

Our study has described the presence of estradiol, progesterone and testosterone in the gonad tissues of *H. scabra* using LC–MS/MS. This contributes to the accumulating evidence for steroids in invertebrates, including echinoderms^8,9^. For example, within the starfish, steroids were extracted and identified from the gonads of *Pisaster ochraceous*^11^, *Asterias rubens*^13,20^, and *Sclerasterias mollis*^21^. Estradiol and progesterone were also extracted from ovaries of the sea urchin *Strongylocentrotus franciscanus*^12^. Although our study did not quantify steroid levels in *H. scabra*, specific concentrations for progesterone, testosterone, and estradiol have been determined from testes and ovaries of the starfish *Asterias vulgaris*^22^ and the echinoid *Lytechinus variegatus*^19^. Levels of testosterone and estradiol were determined in ovaries and testis of the sea urchin *Paracentrotus lividus*^42^. Correlations have been established between steroid levels and the reproductive cycle in a variety of echinoderms^20,21,42–44^. We have additionally shown that estradiol, progesterone and testosterone are present in *H. scabra* neural tissue. While steroids have been described in other invertebrate species neural tissues (called neurosteroids), such as the blue mussel *Mytilus edulis*^45^ and giant freshwater prawn *Macrobrachium rosenbergii*^23^, their existence in echinoderms had not been reported. However, many marine invertebrates can freely absorb vertebrate steroids from the environment and store them for long times, suggesting that the identified sex steroids in the sea cucumber *H. Scabra* may be uptake from exogenous sources^46,47^.

### Proposed steroidogenesis pathway for *H. scabra*

In echinoderms, despite evidence for steroids in various species, the steroidogenesis pathway had not yet been well described. It is well known, however, in the vertebrates that steroidogenesis requires various enzymes associated with biochemical pathways. Primary core enzymes in the pathway include StAR and CYP11, which help to transfer cholesterol across mitochondrial membranes for conversion into pregnenolone. From there, CYP17 is a key steroidogenic enzyme within the pathway for synthesis of testosterone. Also, CYP19 is required for the conversion of androgens into estrogen, while 3β-HSD is essential for synthesis of progesterone, 17beta-hydroxyprogesterone, androstenedione, and testosterone. The 17β-HSD is important for the conversion of dehydroepiandrosterone (DHEA) to androstenediol, androstenedione to testosterone, and estrone to estradiol^10,23,48,49^.

The activity of steroidogenic enzymes has been described in many echinoderm tissues especially gonads, where there is experimental evidence for the conversion of cholesterol into sex steroids that suggested the metabolism of sex steroids^16,18,19,42,44,50–53^. Furthermore, some steroidogenic-related genes have been described in echinoderms^43,44^. In the present study, we support the presence of steroidogenic-related enzymes and biosynthetic pathway for sex steroids in echinoderms by identifying *HscStAR, HscCYP10, HscCYP17, HscCYP3A, Hsc3β-HSD* and *Hsc17β-HSD* genes from in *H. scabra* transcriptome data that are known to be the genes responsible for sex steroid biosynthesis.

The *HscStAR* gene has also been reported in the giant freshwater prawn *M. rosenbergii*^23^, the aquatic snail *Lymnaea palustris*^54^, and the Yesso scallop *Mizuhopecten yessoensis*^10^. Its presence is a key indicator for a core function in steroid synthesis through cholesterol conversion. Further downstream, the HscCYP10 may be important in a role similar to that of the evolutionarily related CYP11, since a *CYP11* gene was not identified in echinoderms and mollusks^46,47^. Phylogenetic analysis indicates that invertebrate *CYP10s* and vertebrate *CYP11s* are located in similar sister clades and shares an evolutionary origin. Similarly, molluscan *CYP10* genes also belong to the cluster of vertebrate *CYP11*^10,55^, suggesting that invertebrate *CYP10* gene encode the steroid metabolizing enzyme. The HscCYP17 genes have also been found in the sea urchin *Strongylocentrotus purpuratus*^48,49^, the mangrove oyster *Crassostrea brasiliana*^56^, the giant freshwater prawn *M. rosenbergii*^23^, and the Yesso scallop *M. yessoensis*^10^, suggesting that CYP17 is could act as a key enzyme in sex steroid synthesis similar to vertebrates. The CYP19 is important in steroid biosynthesis due to its aromatization activity, although can only be found in vertebrate species, but with the exception being amphioxus^46,47,49,57^. Not surprisingly, a CYP19 was not identified in our study. Nevertheless, an aromatization reaction occurs is known to occur in at least some echinoderms^42,44,52,53^, suggesting that aromatization is carried out by another enzyme. In support of this, it was found that aromatase-like enzyme in bivalves *Mytilus trossulus* could perform aromatization, which similar to vertebrate’ aromatase mechanism of action^58^. Recently, other enzymes from the cytochrome P450 family, CYP3A is postulated to be an ancestral gene of CYP19, which could possess aromatization activity, suggesting a potential role for it in converting androgens to estrogens instead of CYP19^10,57,59,60^. The HscCYP3A identified may function as per the vertebrate CYP19. We also identified *Hsc3β-HSD* and *Hsc17β-HSD* within the *H. scabra* transcriptome, of which the *3β-HSD* and *17β-HSD* are known to be involved in steroidogenesis. To date, many isoforms of the 3β-HSD and 17β-HSD have been broadly reported in invertebrates, including echinoderms^10,23,61–67^, which may be involved in the formation of progestogen, estrogens, and androgens. However, sex steroids in invertebrates might be derived from exogenous sources due to a lack of key enzymes involved in steroid biosynthesis^46,47^. It has been demonstrated that exogenous cholesterol is converted to sex steroid hormones in marine invertebrates^68^. Thus, further studies are needed to verify whether sex steroids are synthesized endogenously in invertebrates.

We found using RT-PCR that the steroidogenesis-related genes had a broad tissue distribution in *H. scabra*. In other invertebrates^10,23,64^ and vertebrates^69–71^, steroidogenic enzymes are also expressed in several tissues. This is consistent with the enzymes functional roles in producing steroids, for lipid metabolism and detoxification, and steroidal roles in sexual behaviors, paracrine/autocrine actions, neuronal inhibition and neural plasticity^10,23,70,72,73^.

Previous studies in echinoderms had found correlations between steroid level and changes in reproductive stage, providing evidence that steroids regulate reproductive functions such as oogenesis, vitellogenesis, or spermiogenesis^20–22,48,49^. Steroid levels may be reflected in steroidogenic-associated enzyme expression levels, as demonstrated in the abalone *Haliotis diversicolor supertexta* and Yesso scallop *M. yessoensis* where steroidogenic genes were differentially expressed during its reproductive stages^10,62,63^. Our qPCR analysis of *H. scabra* gonads also found differential expression of steroidogenesis genes during reproductive stage changes. This provides a clear indication of the involvement of steroidogenic genes in reproductive functions. In our study, the expression of *HscStAR* increased during the reproductive development in both ovaries and testes, which corresponds to the expression of *StAR* in fishes^74,75^. We suggest that the expression of *StAR* is necessary for transporting cholesterol into mitochondria for regulating steroid production, resulted in gonad maturation^74^. *HscCYP10* may act as vertebrate *CYP11*, which catalyzes the conversion of cholesterol to pregnenolone^10,55^. The expression of *HscCYP10* obtained in this study is consistent with the expression of CYP11 in fishes, which increased in correlation to gonadal development in both sexes^75,76^. Thus, *HscCYP10* may participate in the synthesis of sex steroids resulting and gonadal development. *HscCYP3A* may play a role in converting androgens to estrogens instead of *CYP19*^10,57,59,60^, which showed a high level of expression at the early stages of ovarian development and decreased at late stages, but low expression in the testes. Similarly, *CYP19* is predominantly expressed in the ovary and its expression was high at the early stage of ovaries in fishes^74,77^. A high correlation between plasma estradiol and expression pattern of ovarian CYP19 has also been demonstrated in vertebrates, which is important for the initiation of follicular growth^77–79^. Our study showed that *HscCYP17* mRNA expression was increased towards the beginning of the reproductive cycle in the testes. However, high expression of *HscCYP17* was detected at the early stage of ovaries and declined at late stages. Consistently, the testis of adult frogs showed an extremely strong expression of *CYP17* as opposed to the ovary^80^. Moreover, *CYP17* transcript levels being low during the early stages, then strongly increasing in maturing male salmon, indicated that androgens production is required during the initiation of spermatogenesis^81^. The expression profile of *Hsc3β-HSD* and *Hsc17β-HSD* were higher when compared to those of the early stages in both ovaries and testes, which is consistent with the expression of *3β-HSD* and *17β-HSD* in vertebrates and invertebrates^10,82^. Moreover, the highest expression level of *Hsc17β-HSD* was detected at stage 5 in the testes. It is well recognized that *17β-HSD* is a steroidogenic enzyme essential for invertebrate spermatogenesis, which functions as a regulator controlling the concentrations of testosterone^63,83^. In addition, the ability to synthesize the cholesterol of echinoderms is very low^68^. Low levels of cholesterol in sea urchin were correlated with plasma membrane lipid diffusion^84^, which may be attributed to reduced cholesterol in the membrane and transported to mitochondria, where steroidogenesis is initiated^85,86^. This is in agreement with our proposed steroidogenesis pathway of *H. scabra* and the potential role of steroidogenic enzymes in steroid production, which is necessary for gonad maturation*.*

The present study also revealed that estradiol and progesterone significantly enhance *Vtg* expression in ovarian explant culture. Similarly, estradiol and progesterone could stimulate the vitellogenesis, which involved in the regulation of reproduction in invertebrates including echinoderms^8,36,87,88^. Moreover, *Vtg* mRNA expression was higher in the estradiol treated group at the beginning of the incubation time, which ovarian tissue is still at developing stage. Subsequently, the expression of *Vtg* was higher in the progesterone treated group, which ovaries turn into mature stage. Consistently, the estradiol level was higher at the beginning of vitellogenesis and concentration of progesterone was higher in mature stage in marine invertebrates^22,36,72^. Taken together, we suggest that sex steroids could control oocyte development and ovarian maturation in *H. scabra* via the regulation of Vtg synthesis.

## Conclusions

We have confirmed the presence of steroids in echinoderms, from both gonad and neural tissues, and further identified and characterized the key steroidogenesis-related genes in the commercially important sea cucumber *H. scabra*. The relative expression of steroidogenesis-related genes throughout gonadal maturation and the effect of steroids on the expression of Vtg further support the idea that steroids can regulate reproduction in echinoderms. Further studies necessary in order to conclude the endogenous synthesis of sex steroids and their role in sea cucumber physiology.

## Supplementary Information

Supplementary Figure Legends.

Supplementary Information 1.

## References

[1] Conand C (1990). The fishery resources of Pacific island countries: Holothurians. Food Agric. Org..

[2] Bordbar S, Anwar F, Saari N (2011). High-value components and bioactives from sea cucumbers for functional foods—A review. Mar. Drugs.

[3] Santos R (2016). Sea cucumber *Holothuria forskali*, a new resource for aquaculture? Reproductive biology and nutraceutical approach. Aquac. Res..

[4] Wen J, Hu C, Fan S (2010). Chemical composition and nutritional quality of sea cucumbers. J. Sci. Food Agric..

[5] Bechtel PJ, Oliveira AC, Demir N, Smiley S (2013). Chemical composition of the giant red sea cucumber, *Parastichopus californicus*, commercially harvested in Alaska. Food Sci. Nutr..

[6] Mamelona J, Saint-Louis R, Pelletier É (2010). Nutritional composition and antioxidant properties of protein hydrolysates prepared from echinoderm byproducts. Int. J. Food Sci. Tech..

[7] Purcell SW, Hair CA, Mills DJ (2012). Sea cucumber culture, farming and sea ranching in the tropics: Progress, problems and opportunities. Aquaculture.

[8] Lafont R, Mathieu M (2007). Steroids in aquatic invertebrates. Ecotoxicology.

[9] Janer G, Porte C (2007). Sex steroids and potential mechanisms of non-genomic endocrine disruption in invertebrates. Ecotoxicology.

[10] Thitiphuree T, Nagasawa K, Osada M (2019). Molecular identification of steroidogenesis-related genes in scallops and their potential roles in gametogenesis. J. Steroid Biochem. Mol. Biol..

[11] Botticelli CR, Hisaw FL, Wortz HH (1960). Estradiol-17β and progesterone in ovaries of starfish (*Pisaster ochraceous*). Proc. Soc. Exp. Biol. Med..

[12] Botticelli CR, Hisaw FL, Wotiz HH (1961). Estrogens and progesterone in the sea urchin (*Strongylocentrotus franciscanus*) and pecten (*Pecten hericius*). Proc. Soc. Exp. Biol. Med..

[13] Dieleman SJ, Schoenmakers HJN (1979). Radioimmunoassays to determine the presence of progesterone and estrone in the starfish *Asterias rubens*. Gen. Comp. Endocrinol..

[14] Schoenmakers HJN (1979). *In vitro* biosynthesis of steroids from cholesterol by the ovaries and pyloric caeca of the starfish *Asterias rubens*. Comp. Biochem. Physiol. B Comp. Biochem..

[15] Schoenmakers HJN, Voogt PA (1980). In vitro biosynthesis of steroids from progesterone by the ovaries and pyloric ceca of the starfish *Asterias rubens*. Gen. Comp. Endocrinol..

[16] Schoenmakers HJN, Voogt PA (1981). In vitro biosynthesis of steroids from androstenedione by the ovaries and pyloric caeca of the starfish *Asterias rubens*. Gen. Comp. Endocrinol..

[17] Voogt PA, Den Besten PJ, Jansen M (1991). Steroid metabolism in relation to the reproductive cycle in *Asterias rubens* L.. Comp. Biochem. Physiol. B Comp. Biochem..

[18] Wasson KM, Watts SA (2000). Progesterone metabolism in the ovaries and testes of the echinoid *Lytechinus variegatus* Lamarck (Echinodermata). Comp. Biochem. Physiol. C Pharmacol. Toxicol. Endocrinol..

[19] Wasson KM, Gower BA, Hines GA, Watts SA (2000). Levels of progesterone, testosterone, and estradiol, and androstenedione metabolism in the gonads of *Lytechinus variegatus*(Echinodermata: Echinoidea). Comp. Biochem. Physiol. C Pharmacol. Toxicol. Endocrinol..

[20] Schoenmakers HJN, Dieleman SJ (1981). Progesterone and estrone levels in the ovaries, pyloric ceca, and perivisceral fluid during the annual reproductive cycle of starfish, *Asterias rubens*. Gen. Comp. Endocrinol..

[21] Xu RA, Barker MF (1990). Effect of diet on steroid levels and reproduction in the starfish, *Sclerasterias mollis*. Comp. Biochem. Physiol. A Comp. Physiol..

[22] Hines GA, Watts SA, Sower SA, Walker CW (1992). Sex steroid levels in the testes, ovaries, and pyloric caeca during gametogenesis in the sea star *Asterias vulgaris*. Gen. Comp. Endocrinol..

[23] Thongbuakaew T (2016). Steroids and genes related to steroid biosynthesis in the female giant freshwater prawn, *Macrobrachium rosenbergii*. Steroids.

[24] Wooding KM (2013). Mechanism of formation of the major estradiol product ions following collisional activation of the molecular anion in a tandem quadrupole mass spectrometer. J. Am. Soc. Mass Spectrom..

[25] Marwah A, Nianzu X, Marwah P, Barry TP (2010). Development and validation of a high performance liquid chromatography-mass spectrometry method for 17a-methyltestosterone in aquatic water systems. J. Appl. Nat. Sci..

[26] Kauppila TJ (2011). Desorption atmospheric pressure photoionization–mass spectrometry in routine analysis of confiscated drugs. Forensic Sci. Int..

[27] Suwansa-ard S (2018). Transcriptomic discovery and comparative analysis of neuropeptide precursors in sea cucumbers (Holothuroidea). Peptides.

[28] Suwansa-ard S (2015). In silico neuropeptidome of female *Macrobrachium rosenbergii* based on transcriptome and peptide mining of eyestalk, central nervous system and ovary. PLoS ONE.

[29] Edgar RC (2004). MUSCLE: Multiple sequence alignment with high accuracy and high throughput. Nucleic Acids Res..

[30] Marchler-Bauer A (2017). CDD/SPARCLE: Functional classification of proteins via subfamily domain architectures. Nucleic Acids Res..

[31] Hunter S (2008). InterPro: The integrative protein signature database. Nucleic Acids Res..

[32] Kumar S, Tamura K, Nei M (2004). MEGA3: Integrated software for molecular evolutionary genetics analysis and sequence alignment. Brief. Bioinform..

[33] Ye J (2012). Primer-BLAST: A tool to design target-specific primers for polymerase chain reaction. BMC Bioinform..

[34] Rasolofonirina R, Vaitilingon D, Eeckhaut I, Jangoux M (2005). Reproductive cycle of edible echinoderms from the southwestern Indian Ocean II. The sandfish Holothuria scabra (Jaeger, 1833). West. Indian Ocean J. Mar. Sci..

[35] Larionov A, Krause A, Miller W (2005). A standard curve based method for relative real time PCR data processing. BMC Bioinform..

[36] Merlin J (2015). Induction of vitellogenesis and reproductive maturation in tiger shrimp, *Penaeus monodon* by 17ß-estradiol and 17α-hydroxyprogesterone: In vivo and in vitro studies. Invertebr. Reprod. Dev..

[37] Thongbuakaew T (2019). Identification and characterization of a crustacean female sex hormone in the giant freshwater prawn, *Macrobrachium rosenbergii*. Aquaculture.

[38] Alpy F (2001). The steroidogenic acute regulatory protein homolog MLN64, a late endosomal cholesterol-binding protein. J. Biol. Chem..

[39] Chen CD, Kemper B (1996). Different structural requirements at specific proline residue positions in the conserved proline-rich region of cytochrome P450 2C2. J. Biol. Chem..

[40] Córdova P (2017). Characterization of the cytochrome P450 monooxygenase genes (P450ome) from the carotenogenic yeast *Xanthophyllomyces dendrorhous*. BMC Genom..

[41] Alqaisi KM (2016). A comparative study of vitellogenesis in Echinodermata: Lessons from the sea star. Comp. Biochem. Physiol. Part A Mol. Integr. Physiol..

[42] Barbaglio A (2007). Gametogenesis correlated with steroid levels during the gonadal cycle of the sea urchin *Paracentrotus lividus* (Echinodermata: Echinoidea). Comp. Biochem. Physiol. A Mol. Integr. Physiol..

[43] Voogt PA, Dieleman SJ (1984). Progesterone and oestrone levels in the gonads and pyloric caeca of the male sea star *Asterias rubens*: A comparison with the corresponding levels in the female sea star. Comp. Biochem. Physiol. A Physiol..

[44] Barbaglio A, Sugni M, Fernandes D, Porte C, Carnevali MDC (2012). Reproductive cycle and sex hormones in the feather star *Antedon mediterranea*. J. Exp. Mar. Biol. Ecol..

[45] Stefano GB (2003). Estrogen signaling at the cell surface coupled to nitric oxide release in *Mytilus edulis* nervous system. Endocrinology.

[46] Scott AP (2012). Do mollusks use vertebrate sex steroids as reproductive hormones? Part I: Critical appraisal of the evidence for the presence, biosynthesis and uptake of steroids. Steroids.

[47] Scott AP (2018). Is there any value in measuring vertebrate steroids in invertebrates?. Gen. Comp. Endocrinol..

[48] Baker ME, Nelson DR, Studer RA (2015). Origin of the response to adrenal and sex steroids: Roles of promiscuity and co-evolution of enzymes and steroid receptors. J. Steroid Biochem. Mol. Biol..

[49] Goldstone JV (2016). Genetic and structural analyses of cytochrome P450 hydroxylases in sex hormone biosynthesis: Sequential origin and subsequent coevolution. Mol. Phylogenetics Evol..

[50] Wasson KM, Hines GA, Watts SA (1998). Synthesis of testosterone and 5α-androstanediols during nutritionally stimulated gonadal growth in *Lytechinus variegatus* Lamarck (Echinodermata: Echinoidea). Gen. Comp. Endocrinol..

[51] Janer G, LeBlanc GA, Porte C (2005). A comparative study on androgen metabolism in three invertebrate species. Gen. Comp. Endocrinol..

[52] Lavado R, Barbaglio A, Carnevali MDC, Porte C (2006). Steroid levels in crinoid echinoderms are altered by exposure to model endocrine disruptors. Steroids.

[53] Lavado R, Sugni M, Carnevali MDC, Porte C (2006). Triphenyltin alters androgen metabolism in the sea urchin *Paracentrotus lividus*. Aquat. Toxicol..

[54] Reddy SB, Nolan CJ, Plautz CZ (2018). Disturbances in reproduction and expression of steroidogenic enzymes in aquatic invertebrates exposed to components of the herbicide Roundup. Toxicol. Res. Appl..

[55] Guo H (2013). Identification of Cytochrome P450 (CYP) genes in Zhikong scallop (*Chlamys farreri*). J. Ocean Univ. China.

[56] Lüchmann KH (2015). Key metabolic pathways involved in xenobiotic biotransformation and stress responses revealed by transcriptomics of the mangrove oyster *Crassostrea brasiliana*. Aquat. Toxicol..

[57] Markov GV (2009). Independent elaboration of steroid hormone signaling pathways in metazoans. Proc. Natl. Acad. Sci. USA..

[58] Hallmann A, Konieczna L, Swiezak J, Milczarek R, Smolarz K (2019). Aromatisation of steroids in the bivalve *Mytilus trossulus*. PeerJ.

[59] Sun H (2007). Dehydrogenation of indoline by cytochrome P450 enzymes: A novel "aromatase" process. J. Pharmacol. Exp. Ther..

[60] Cubero-Leon E (2012). Two CYP3A-like genes in the marine mussel *Mytilus edulis*: mRNA expression modulation following short-term exposure to endocrine disruptors. Mar. Environ. Res..

[61] Mindnich R, Adamski J (2009). Zebrafish 17beta-hydroxysteroid dehydrogenases: An evolutionary perspective. Mol. Cell. Endocrinol..

[62] Zhou J (2011). Identification and functional characterization of a putative 17beta-hydroxysteroid dehydrogenase 12 in abalone (*Haliotis diversicolor supertexta*). Mol. Cell. Biochem..

[63] Zhai HN, Zhou J, Cai ZH (2012). Cloning, characterization, and expression analysis of a putative 17 beta-hydroxysteroid dehydrogenase 11 in the abalone, *Haliotis diversicolor supertexta*. J. Steroid Biochem. Mol. Biol..

[64] Zhang Y (2014). Identification and mRNA expression of two 17β-hydroxysteroid dehydrogenase genes in the marine mussel *Mytilus galloprovincialis* following exposure to endocrine disrupting chemicals. Environ. Toxicol. Pharmacol..

[65] Liu J, Zhang Z, Ma X, Liang S, Yang D (2014). Characteristics of 17β-hydroxysteroid dehydrogenase 8 and its potential role in gonad of Zhikong scallop *Chlamys farreri*. J. Steroid Biochem. Mol. Biol..

[66] Aceves-Ramos A (2014). Cloning, characterization and functional expression of *Taenia solium* 17 beta-hydroxysteroid dehydrogenase. Gen. Comp. Endocrinol..

[67] Meng XL, Liu P, Jia FL, Li J, Gao BQD (2015). De novo transcriptome analysis of *Portunus trituberculatus* ovary and testis by RNA-Seq: Identification of genes involved in gonadal development. PLoS ONE.

[68] Kanazawa A (2001). Sterols in marine invertebrates. Fish. Sci..

[69] Bauer MP, Bridgham JT, Langenau DM, Johnson AL, Goetz FW (2000). Conservation of steroidogenic acute regulatory (StAR) protein structure and expression in vertebrates. Mol. Cell. Endocrinol..

[70] Pezzi V, Mathis JM, Rainey WE, Carr BR (2003). Profiling transcript levels for steroidogenic enzymes in fetal tissues. J. Steroid Biochem. Mol. Biol..

[71] Peek CE, Cohen RE (2018). Seasonal regulation of steroidogenic enzyme expression within the green anole lizard (*Anolis carolinensis*) brain and gonad. Gen. Comp. Endocrinol..

[72] Forlano PM, Deitcher DL, Myers DA, Bass AH (2001). Anatomical distribution and cellular basis for high levels of aromatase activity in the brain of teleost fish: Aromatase enzyme and mRNA expression identify glia as source. J. Neurosci..

[73] Belelli D (2006). Neuroactive steroids and inhibitory neurotransmission: Mechanisms of action and physiological relevance. Neuroscience.

[74] Rocha A, Zanuy S, Carrillo M, Gómez A (2009). Seasonal changes in gonadal expression of gonadotropin receptors, steroidogenic acute regulatory protein and steroidogenic enzymes in the European sea bass. Gen. Comp. Endocrinol..

[75] Kusakabe M (2002). Characterization and expression of steroidogenic acute regulatory protein and MLN64 cDNAs in trout. Endocrinology.

[76] Nishiyama M, Uchida K, Abe N, Nozaki M (2015). Molecular cloning of cytochrome P450 side-chain cleavage and changes in its mRNA expression during gonadal development of brown hagfish, *Paramyxine atami*. Gen. Comp. Endocrinol..

[77] Kumar RS, Ijiri S, Trant JM (2000). Changes in the expression of genes encoding steroidogenic enzymes in the channel catfish (*Ictalurus punctatus*) ovary throughout a reproductive cycle. Biol. Reprod..

[78] Tripathy M, Rai U (2017). Temporal expression and gonadotropic regulation of aromatase and estrogen receptors in the ovary of wall lizard, *Hemidactylus flaviviridis*: Correlation with plasma estradiol and ovarian follicular development. Steroids.

[79] Rashid H (2007). Fugu (*Takifugu rubripes*) sexual differentiation: CYP19 regulation and aromatase inhibitor induced testicular development. Sex. Dev..

[80] Iwade R, Maruo K, Okada G, Nakamura M (2008). Elevated expression of P450c17 (CYP17) during testicular formation in the frog. Gen. Comp. Endocrinol..

[81] Maugars G, Schmitz M (2008). Gene expression profiling during spermatogenesis in early maturing male Atlantic salmon parr testes. Gen. Comp. Endocrinol..

[82] Santillo A, Falvo S, Chieffi Baccari G, Di Fiore MM (2017). Seasonal changes in gene expression of steroidogenic enzymes, androgen and estrogen receptors in frog testis. Acta Zool..

[83] Kho KH, Inaba K (2004). Expression of 17beta-hydroxysteroid dehydrogenease in testis of the ascidian *Ciona intestinalis*. Mol. Cells.

[84] Weaver FE, Shaikh SR, Edidin M (2008). Plasma membrane lipid diffusion and composition of sea urchin egg membranes vary with ocean temperature. Chem. Phys. Lipids..

[85] Freeman DA (1989). Plasma membrane cholesterol: Removal and insertion into the membrane and utilization as substrate for steroidogenesis. Endocrinology.

[86] Venugopal S (2016). Plasma membrane origin of the steroidogenic pool of cholesterol used in hormone-induced acute steroid formation in Leydig cells. J. Biol. Chem..

[87] Takahashi N, Kanatani H (1981). Effect of 17β-estradiol on growth of oocytes in cultured ovarian fragments of the starfish, *Asterina pectinifera*. Dev. Growth Differ..

[88] Barker MF, Xu RA (1993). Effects of estrogens on gametogenesis and steroid levels in the ovaries and pyloric caeca of *Sclerasterias mollis* (Echinodermata: Asteroidea). Invertebr. Reprod. Dev..

