# Supplementary material for: Sex steroids and steroidogenesis-related genes in the sea cucumber, Holothuria scabra and their potential role in gonad maturation
Source: Sci Rep. 2021 Jan 26;11:2194. doi: 10.1038/s41598-021-81917-x (PMC7838161; doi:10.1038/s41598-021-81917-x)
Supplement: Supplementary file 1 — Supplementary Figure Legends. [file 41598_2021_81917_MOESM1_ESM.docx]

**Figure S1** Multiple sequence alignment of *H. scabra* cytochrome P450 genes associated with steroidogenesis. **(A)** Alignment of CYP10 and CYP11 sequences shows conservation within PERF/W and KET/S(x)R(x)P(x)R motifs but proline-rich motif (yellow highlights). **(B)** Alignment of CYP17 demonstrates conservation within heme-binding and PEHF motifs (yellow highlights). **(C)** Alignment of CYP3A reveals conservation within PERF and heme-binding regions (yellow highlights). Multiple sequence alignment represented by * = identical, : = strong homology, and . = less homology.

**Figure S2** Multiple sequence alignment of *H. scabra* hydroxysteroid dehydrogenases (HSDs). **(A)** Alignment of 3β-HSD demonstrates conservation within NAD-binding motif (yellow highlight). **(B)** Alignment of 17β-HSD reveals conservation within NNAG and YxxxK motifs (yellow highlights). Multiple sequence alignment represented by * = identical, : = strong homology, and . = less homology.
