# Supplementary material for: Sex steroids and steroidogenesis-related genes in the sea cucumber, Holothuria scabra and their potential role in gonad maturation
Source: Sci Rep. 2021 Jan 26;11:2194. doi: 10.1038/s41598-021-81917-x (PMC7838161; doi:10.1038/s41598-021-81917-x)

Title: **Sex steroids and steroidogenesis-related genes in the sea cucumber, *Holothuria scabra* and their potential role in gonad maturation**

Authors: Tipsuda Thongbuakaew[a*], Saowaros Suwansa-ard[b], Arada Chaiyamoon[c], Scott F. Cummins[b], Prasert Sobhon[d]

Affiliations:

[a] School of Medicine, Walailak University, Nakhon Si Thammarat 80161, Thailand

[b] Genecology Research Centre, School of Science and Engineering, University of the Sunshine Coast, Sippy Downs, Queensland 4556, Australia

[c] Department of Anatomy, Faculty of Medicine, Khon Kaen University, Khon Kaen 40002, Thailand

[d] Department of Anatomy, Faculty of Science, Mahidol University, Bangkok 10400, Thailand

* **Corresponding authors**: Tipsuda Thongbuakaew – tipsuda.th@wu.ac.th

Figure S1

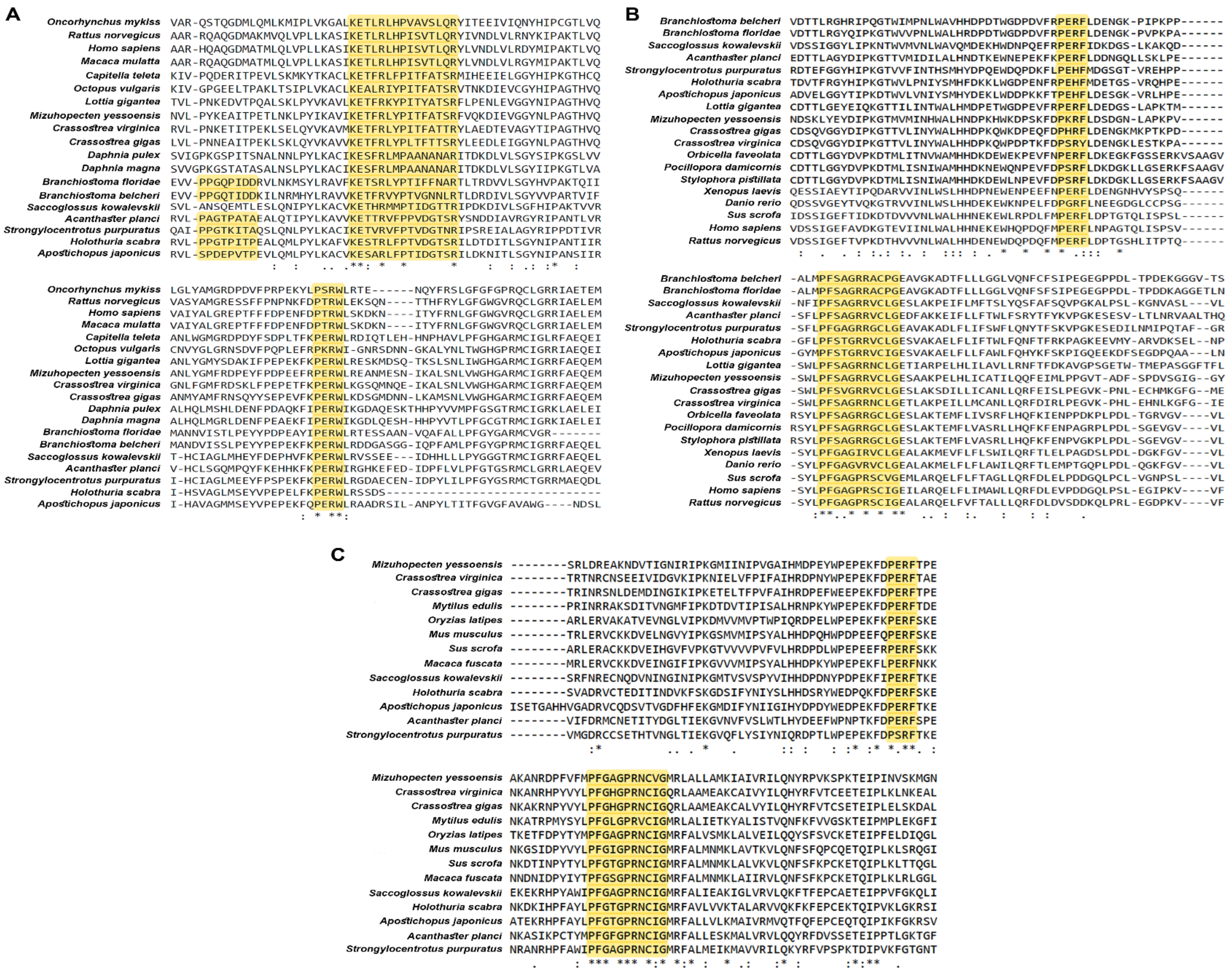

Figure S2

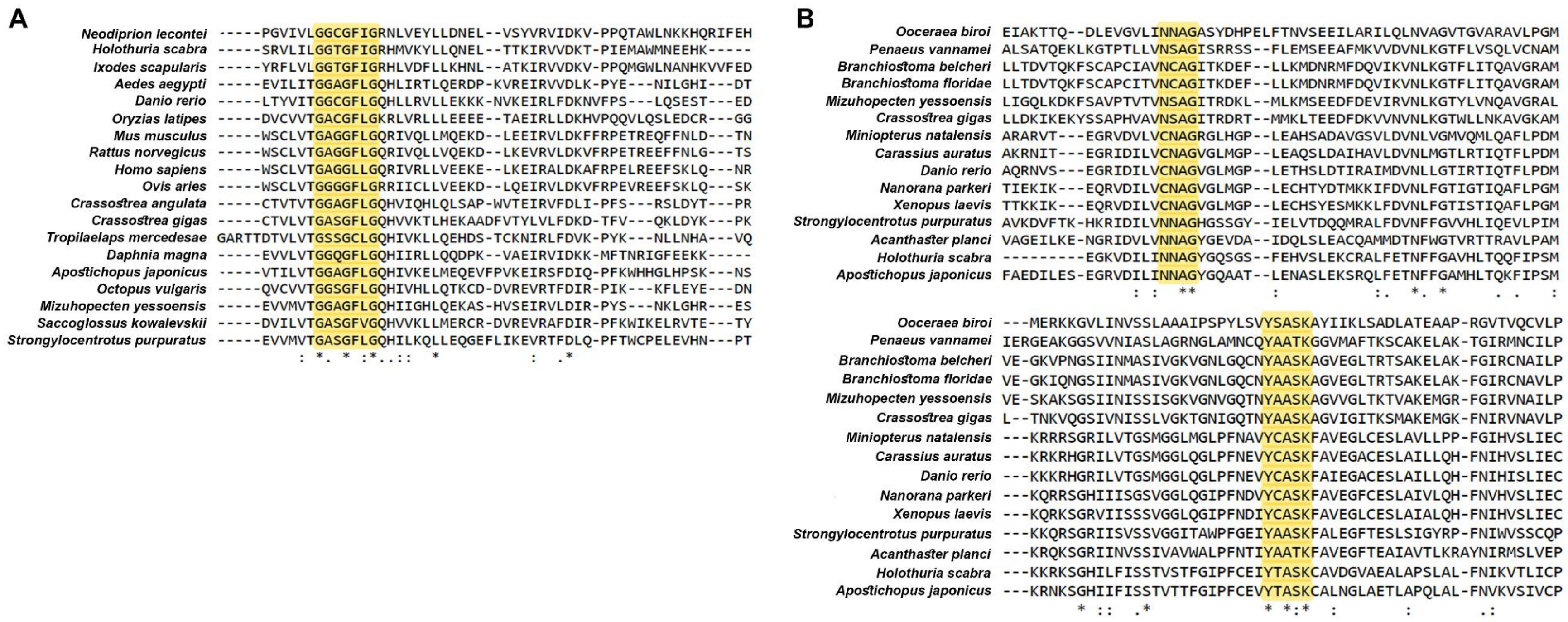
A
Neodiprion lecontei -----PGVIVLGGCGFIGRNLVEYLLDNEL--VSYVRVIDKV-PPQTAWLNKKHQRIFEH
Holothuria scabra -----SRVLILGGTGFIGRHMVKYLLQNEL--TTKIRVVDKT-PIEMAWMNEEHK-----
Ixodes scapularis -----YRFLVLGGTGFIGRHLVDFLLKHNL--ATKIRVVDKV-PPQMGWLNANHKVVFED
Aedes aegypti -----EVILITGGAGFLGQHLIRTLQERDP-KVREIRVVDLK-PYE---NILGHI---DT
Danio rerio -----LTYVITGGCGFLGQHLLRVLLEKKK-NVKEIRLFDKNVFPS---LQSEST---ED
Oryzias latipes -----DVCVVTGACGFLGKRLVRLLLEEEE--TAEIRLLDKHVPQQVLQSLEDCR---GG
Mus musculus -----WSCLVTGAGGFLGQRIVQLLMQEKD--LEEIRVLDKFFRPETREQFFNLD---TN
Rattus norvegicus -----WSCLVTGAGGFLGQRIVQLLVQEKD--LKEVRVLDKVFRPETREEFFNLG---TS
Homo sapiens -----WSCLVTGAGGLLGQRIVRLLVEEKE--LKEIRALDKAFRPELREEFSKLQ---NR
Ovis aries -----WSCLVTGGGGFLGRRIICLLVEEKD--LQEIRVLDKVFRPEVREEFSKLQ---SK
Crassostrea angulata -----CTVTVTGGAGFLGQHVIQHLQLSAP-WVTEIRVFDLI-PFS---RSLDYT---PR
Crassostrea gigas -----CTVLVTGASGFLGQHVVKTLHEKAADFVTYLVLFDKD-TFV---QKLDYK---PK
Tropilaelaps mercedesae GARTTDTVLVTGSSGCLGQHIVKLLQEHDS-TCKNIRLFDVK-PYK---NLLNHA---VQ
Daphnia magna -----EVVLVTGGQGFLGQHIIRLLQQDPK--VAEIRVIDKK-MFTNRIGFEEKK-----
Apostichopus japonicus -----VTILVTGGAGFLGQHIVKELMEQEVFPVKEIRSFDIQ-PFKWHHGLHPSK---NS
Octopus vulgaris -----QVCVVTGGSGFLGQHIVHLLQTKCD-DVREVRTFDIR-PIK---KFLEYE---DN
Mizuhopecten yessoensis -----EVVMVTGGAGFLGQHIIGHLQEKAS-HVSEIRVLDIR-PYS---NKLGHR---ES
Saccoglossus kowalevskii -----DVILVTGASGFVGQHVVKLLMERCR-DVREVRAFDIR-PFKWIKELRVTE---TY
Strongylocentrotus purpuratus -----EVVMVTGASGFLGQHILKQLLEQGEFLIKEVRTFDLQ-PFTWCPELEVHN---PT
: *. * :*..:: * : .*
B
Ooceraea biroi EIAKTTQ--DLEVGVLINNAGASYDHPELFTNVSEEILARILQLNVAGVTGVARAVLPGM
Penaeus vannamei ALSATQEKLKGTPTLLVNSAGISRRSS--FLEMSEEAFMKVVDVNLKGTFLVSQLVCNAM
Branchiostoma belcheri LLTDVTQKFSCAPCIAVNCAGITKDEF--LLKMDNRMFDQVIKVNLKGTFLITQAVGRAM
Branchiostoma floridae LLTDVTQKFSCAPCITVNCAGITKDEF--LLKMDNRMFDQVIKVNLKGTFLITQAVGRAM
Mizuhopecten yessoensis LIGQLKDKFSAVPTVTVNSAGITRDKL--MLKMSEEDFDEVIRVNLKGTYLVNQAVGRAL
Crassostrea gigas LLDKIKEKYSSAPHVAVNSAGITRDRT--MMKLTEEDFDKVVNVNLKGTWLLNKAVGKAM
Miniopterus natalensis ARARVT---EGRVDVLVCNAGRGLHGP--LEAHSADAVGSVLDVNLVGMVQMLQAFLPDM
Carassius auratus AKRNIT---EGRIDILVCNAGVGLMGP--LEAQSLDAIHAVLDVNLMGTLRTIQTFLPDM
Danio rerio AQRNVS---EGRIDILVCNAGVGLMGP--LETHSLDTIRAIMDVNLLGTIRTIQTFLPDM
Nanorana parkeri TIEKIK---EQRVDILVCNAGVGLMGP--LECHTYDTMKKIFDVNLFGTIGTIQAFLPGM
Xenopus laevis TTKKIK---EQRVDILVCNAGVGLMGP--LECHSYESMKKLFDVNLFGTISTIQAFLPGM
Strongylocentrotus purpuratus AVKDVFTK-HKRIDILVNNAGHGSSGY--IELVTDQQMRALFDVNFFGVVHLIQEVLPIM
Acanthaster planci VAGEILKE-NGRIDVLVNNAGYGEVDA--IDQLSLEACQAMMDTNFWGTVRTTRAVLPAM
Holothuria scabra ---------EGKVDILINNAGYGQSGS--FEHVSLEKCRALFETNFFGAVHLTQQFIPSM
Apostichopus japonicus FAEDILES-EGRVDILINNAGYGQAAT--LENASLEKSRQLFETNFFGAMHLTQKFIPSM
: : ** : :. *. * . . :
Ooceraea biroi ---MERKKGVLINVSSLAAAIPSPYLSVYSASKAYIIKLSADLATEAAP-RGVTVQCVLP
Penaeus vannamei IERGEAKGGSVVNIASLAGRNGLAMNCQYAATKGGVMAFTKSCAKELAK-TGIRMNCILP
Branchiostoma belcheri VE-GKVPNGSIINMASIVGKVGNLGQCNYAASKAGVEGLTRTSAKELAK-FGIRCNAILP
Branchiostoma floridae VE-GKIQNGSIINMASIVGKVGNLGQCNYAASKAGVEGLTRTSAKELAK-FGIRCNAVLP
Mizuhopecten yessoensis VE-SKAKSGSIINISSISGKVGNVGQTNYAASKAGVVGLTKTVAKEMGR-FGIRVNAILP
Crassostrea gigas L--TNKVQGSIVNISSLVGKTGNIGQTNYAASKAGVIGITKSMAKEMGK-FNIRVNAVLP
Miniopterus natalensis ---KRRRSGRILVTGSMGGLMGLPFNAVYCASKFAVEGLCESLAVLLPP-FGIHVSLIEC
Carassius auratus ---KRKRHGRILVTGSMGGLQGLPFNEVYCASKFAVEGACESLAILLQH-FNIHVSLIEC
Danio rerio ---KKKRHGRILVTGSMGGLQGLPFNEVYCASKFAIEGACESLAILLQH-FNIHISLIEC
Nanorana parkeri ---KQRRSGHIIISGSVGGLQGIPFNDVYCASKFAVEGFCESLAIVLQH-FNVHVSLIEC
Xenopus laevis ---KQRKSGRVIISSSVGGLQGIPFNDIYCASKFAVEGLCESLAIALQH-FNIHVSLIEC
Strongylocentrotus purpuratus ---KKQRSGRIISVSSVGGITAWPFGEIYAASKFALEGFTESLSIGYRP-FNIWVSSCQP
Acanthaster planci ---KRQKSGRIINVSSIVAVWALPFNTIYAATKFAVEGFTEAIAVTLKRAYNIRMSLVEP
Holothuria scabra ---KKRKSGHILFISSTVSTFGIPFCEIYTASKCAVDGVAEALAPSLAL-FNIKVTLICP
Apostichopus japonicus ---KRNKSGHIIFISSTVTTFGIPFCEVYTASKCALNGLAETLAPQLAL-FNVKVSIVCP
* :: .* * *:* : : .:

S1. List of genes and their amino acid sequences found in *H. scabra* involved in the steroidogenesis pathway. P; Partial sequence, F; Full-length sequence.

Steroidogenic-related genes	Transcripts	Length (aa)	BLAST hit and species	E-value	Accession numbers	Sequences
**steroidogenic activator**						
steroidogenic acute regulatory protein (StAR)	contig_2100	F/381	steroidogenic acute regulatory protein, mitochondrial-like [Strongylocentrotus purpuratus]	7.00E-69	XP_011660912.1	MADTEQKVEETPAAEEPKKPAEEEKPVENGEKTEEAVADGDAKTEEAKEEGGEAKEEAVENHENGKEGEEEEEEETPAPSLEAPTENPFTDEDVQQLLDMESGEEGWDKVKEKKGVLVYRKAEKNSSPLIKGVLEYSGIPTEKILEFYTDFEVRRKWNKKTINIEVLDEKDDFKIIHSIFKMPTGCDNRDAVQCTMVRSEEDGSRHVILYKSCNHPSKPPKKSFPIRADVQLMGAVIRPKEGEEGVTTVTFINQVNLKGWMPKAFVNKVTVGFPVALYDDLTLYWKKITEPPKSPEKKDKKNKEKNGEENGEAGEEEKNDEKKEEEEEENKEADVETEKKDEGAAAEGAAEAAGEGAPVGEGDGGKLDTADIPAADEEEAK
StAR-related lipid transfer protein	contig_1443	P/184	stAR-related lipid transfer protein [Strongylocentrotus purpuratus]	2.00E-91	XP_789877.3	KEVKVLYKKSTEFDGHIYRAECVIDSPAEKVIRYILPSPRGLRGKWDKTVKESEVIEKIDEDLIVGRSATHSVAMGLISSRDFVDLITVRRYPEESIICTNSKSIQRKDCPPKDGFVRGVNYHGGTFCTPVEGEPDKCYVVNIVQTDLSGMLPKSVVESALPTNLIDFFRDLDEALKDNNLNCT
steroid receptor RNA activator 1	contig_22299	F/238	steroid receptor RNA activator 1 [Orycteropus afer afer]	1.00E-24	XP_007937187.1	MADKFSGSGARGWNDPPMLQSAISGQNNNSSQPKKQLTQRVTHILPENGPSPPKTGDLKPPTEQIPLSAPRPKMPENLPPPPSELMKPKETEKGEETTSGTSSGIPEDLLPTLEEVLSKLLSTLEKCQGQLKARVCDDVKRKLNIFEKCWMDDKLSNPVKIQMGKLATAITDRKYEEANRLHLGLMMNHVSEVSSWMVGIKRLIQEAKGILPEETDTTAQSVDSKQEAPSILIPVTS
androgen-induced gene 1 protein	contig_55776	P/233	androgen-induced gene 1 protein isoform X1 [Strongylocentrotus purpuratus]	2.00E-64	XP_793283.4	ASLFVTTPLHFLLFYHYATTYWFYLTDTKERMYAARLYTFGGEFKYLTIWTMLLQLFYFFWCCVTDLISASASETSVGKAAVAVRDWFLAAIVFPIGTLVVTTFWAIYSIDRELIFPEALDKFFPSWLNHALHTYVLPLLLVDMLLVNHKYPSRRAGMAGTLAFGFSYLIWVLFLGFAKDIWVYPFFKVLHGIYFPLFFGVMSLVLFLFYIIGEKTNALLGGYRKEVSTEKMD

steroid hormone receptor

steroid hormone receptor 3	contig_4954	P/547	steroid hormone receptor 3 [Strongylocentrotus purpuratus]	0.00E+00	NP_001020384.1	MKLPPSKRWSSFHPPHSHCASPSTSPPHYPRSPPLHSLSSPRHVPPLLVVPGIFQSCSQNKSPVGDPYFVKSPHSTVIESSYPLTLSPYGHGTLSSPGSLSSLSQPPASLGVSRSAGFLPSPGSIGSGGVMLSPFSAGPTPRPGSTRFPFPPHLSVQTHHHMLHDSIETDSRLAKPSGRGSAGLDSQGHSDESNSDEDEALMLCSICSDKATGLHYGIITCEGCKGFFKRTVQNKRIYTCVGTGNCEVTKAQRNRCQYCRFQKCLTMGMMLEAVREDRMPGGRNTGLSYKCKPKNYDKLRKKFQLMAQQKAQMNRKNRAEKAAIKAAREANKGQGENKSKDPPTAGSDKRKPEIRQLKPQTAQLVEVLQQTESAVLKIANKDWREQLKIPSTLELSEFVRLQCMLGEQLVFHLVQWVKHLPFFTQLSAVEHTYILKTKWLELLLLCTVTQAMHFRQKSLGSSGNTTNPEVGLSFELCAHQNLLTLQECMNKTLDLQLDMDVFREEMGDVVEKITKLAASFRTLDIKRNEYLLLKVIVLLNQANGSHS
steroid hormone receptor spshr2	contig_61406	P/135	steroid hormone receptor spshr2 [Strongylocentrotus purpuratus]	2.00E-57	NP_001116968.1	VKLREDDGTSQGLSQIQIKSAAFAPQRPVELCVVCGDKASGRHYGAISCEGCKGFFKRSIRKSLGYTCRGSKECQIIKHNRNRCQYCRLQKCLAMGMKSDLQCERSPLKPREKTPDKVPARTGLFDQGILLNIQP
orphan steroid hormone receptor 2-like	contig_15452	P/316	orphan steroid hormone receptor 2-like [Saccoglossus kowalevskii]	6.00E-122	XP_002739506.2	IRKDIRSPLAATPTFGTDKVPARTGLFDQGILLNIQPNTTSTVQPTQTTDTTTTDLSTLASVVTSLASMKGNTEVQDGAAGDAQMVISNGETSTVTVTSQPETSSQISKAFDTLAKALNPAADGDGTNSSTSGSEQTLTLSGGSANNSTEQPLIELEGPMLTDTHTQFKLTTPSPMPQYLNVHYICESASRLLFLSMHWARSIPAFQALSPDCHTVMVQKCWSELFTLGLAQCAQSMALSTILTAIVNHLQTSLQQDKLTADRVKAVMEHIWKLQEFVSATSKLCIDQVEFAYLKTIVLFSPDHPGLVNPRQVERF
membrane-associated progesterone receptor component	contig_13885	F/175	membrane-associated progesterone receptor component 1 [Strongylocentrotus purpuratus]	7.00E-75	XP_783332.1	MAAVELLKELFLNPINVALLSVCVFLLYKIFAGGRRKPEPPKQPQLPRMKRRDFTLQDLKKYNGVDDERILIAVNGQVFDVTRGKNFYGPDGPYGIFGGHDASRALATFSLDKDSLPEEYDDLSDLNAEQMGSVREWHMQFQEKYDYVGKLLKPGEAPTDYSDEESNAEGDKKDS

estrogen-related receptor-like (ERR)	contig_41919	P/205	steroid hormone receptor ERR2-like [Lingula anatina]	3.00E-41	XP_013405267.1	MTSEQAPPQRDAVDLCVRQQTDSSYTSTNNRSQYEGSTRNVRLCLVCSDTATGCHYSVITCEGCKGFFKRTVQKQLNYTCREGSEGCVIDKLNRNVCQYCRFQKCLQVGMKTEAVREDRMPGGLPKAKRAKVSRDRHGKDRPPSDPEIGTSQLTRVAEEVDWQSVLKPILDAGPDLVPDTESLNSDTCSLNEMMKVGYMELNMII
**steroidogenic enzymes**						
17-beta-hydroxysteroid dehydrogenase type 1	contig_13797	P/212	estradiol 17-beta-dehydrogenase 1 [Miniopterus natalensis]	6.00E-31	XP_016057672.1	LSREGKVDILINNAGYGQSGSFEHVSLEKCRALFETNFFGAVHLTQQFIPSMKKRKSGHILFISSTVSTFGIPFCEIYTASKCAVDGVAEALAPSLALFNIKVTLICPGPVETNFAPNIIMFGQDENDEETMDIKNQYLKNLQQIIGENLQKRDEVAAVIKEAIEAEKPNLRYYTSEKDLQDAKNKLVDTTGNCAIEFLNNSYFKGVKLNSQ
17-beta-hydroxysteroid dehydrogenase type 2	contig_63395	P/170	estradiol 17-beta-dehydrogenase 2-like [Elephantulus edwardii]	1.00E-41	XP_006888866.1	SCSNRLVTIQMDVTKEDSIQNAVKEVKEKLQGRDLWALVNNAGILYMGEAEILSIKFARNMMDVNFMGAVRLTQAFLPMLRQSQGRVVSLCSLGSDVAIPFFGVYCASKAALKSFMDVLRLEMLKWNVLVSTIHPVGYKTALLGKENLKNKLEAVYHDMEPSLKEDYGPH
17-beta-hydroxysteroid dehydrogenase type 4	contig_19638	F/738	17 beta hydroxysteroid dehydrogenase 4 [Salmo trutta fario]	0.00E+00	ACN66287.1	MNQGPQEDSNMLRFADRVAVVTGAGGGLGREYALLFASRGASVVVNDLGGDTKGGGKSSAAADKVVQEIRDKGGKAVPNYDSVEDGEKIIETALKNFGRVDILVNNAGILRDRSFARTSDQDWDLIHRVHLRGSFLVSRAAWPHMKKQNYGRIIMTASAAGIYGNFGQSNYSAAKLGLLGLSNTLAIEGRKNNILCNTIAPLAISRMTEDILPDEMKPMLKPVNVAPLVAYLCHEDSDETGGLFEVGGGWMGKLRWQRANGAVCLSAGEGFSPESVKENWDKITDFDDGIFPASVVESMTSIVNSIRDAQSSANPSDPPSSSDNPNDAIGHKLPTNHFAFTNRDCMLYNLGVGVSTEQPDYLKFLFELNEDFCVLPTFGVIPAFSALQSLLSGSVPGLSGVNPARILHGEQFLEIVKPIPTSGTLRNEAVVTDVLDKKSGAVILMDINSYDENDELVVRNQYSTFAVGSGGFGGKRSSDKAIPTVNKPDRAPDATMSEKTSIDQAALYRLNGDYNPLHIDPSFAAMGGFDTPILHGLCTFGYAGRHVLKTFCDNDVTKFKAIKVRFSGVVLPGQTIQTDMWKEGNRILFNSKVAETGKDCITGAYVDLFEGAQSQQQTSSSGSLKSDLVFSEIGKHLAADSSLYKKVDGIIAYKITSGGAPAKTWTLDLKTPPGSVYEGDPKGGKADATVTIDDEDFADVASGSKNAQTLFQSGKLKASGNIMLLMKMGSLMQRPSKM

17-beta-hydroxysteroid dehydrogenase type 12	contig_36373, 34365	F/331	estradiol 17-beta-dehydrogenase 12 [Saccoglossus kowalevskii]	9.00E-54	XP_002733503.1	MEDKQLFGRFNTVLAGLGALCALYVAAKTAHFLLKSIKMFVLRLGTNPKKYGTWAAVTGATDGIGKAYAEQLASKGLNVVLLSRTESKLETVASEIRSKYNVETKIIAVDFTGGAEIYSDIAAKLEGLEVGVLVNNVGMSYSYPQFFCELPEAEKLIPSLINVNCLSLTMMTNIVLPSMLSRKKGLIINVASAAGCLPSPLLSIYSGTKAYVDFFSRSLNTEYASKGIVVQSVLPFFVVSKMSKIRKSSLTVPTATSYVKSALATVGMETRTFGCFMHNLQGAVGTSFPESIVKFVVMVMLLGARKRTMKEEQRKREQKGDGKSTNPKKDI
17-beta-hydroxysteroid dehydrogenase type 13	contig_27928	P/280	17-beta-hydroxysteroid dehydrogenase 13-like [Saccoglossus kowalevskii]	3.00E-85	XP_002732320.1	MNFILDFLVDFVTLILKLNWYFLEVVSHIFVAPPRKSVEGQVVLVTGAGHGIGRVMAKKFAEQKAKVVIWDINEAGNSRTAKEIQTLGGEVHSYTVDVSNKDKVYDAAARVKKDVGKVDILVNNAGILNGELLLELTDNQIQRVMDINVMAHFWTIRAFLPDMISSNKGHVVTISSASGYFGKNFLVDYTASKFAVTGLDEALEVELIKNYNNPDVHLTIVHPFVIQTGLVHGFTSRFMPMVDEEYAGEAIVDGVLRNRRKVFIPKDLRFSIIVKYIFPH
17-beta-hydroxysteroid dehydrogenase type 14	contig_13478	P/162	17-beta-hydroxysteroid dehydrogenase 14-like [Plutella xylostella]	3.00E-70	XP_011558998.1	PLLVTGDISKEEDNVNLVKKTVDHYGQLDILVNNAGILKLGNIETTKLEDFDEMMNVNVRSIFQLTQLAVPHLLKTKGNVVNISSVNGLRSFANVLAYCVSKSAVDQMTSCIALELADRGVRVNSVNPGVVVTELQKRGGLDDEKYAAFLERSKQTHALGRP
3-beta-hydroxysteroid dehydrogenase	contig_33425	P/54	3-beta-hydroxysteroid dehydrogenase, putative [Ixodes scapularis]	6.00E-13	XP_002407062.1	MSSDWIGSRVLILGGTGFIGRHMVKYLLQNELTTKIRVVDKTPIEMAWMNEEHK
steroid 17-alpha-hydroxylase/17,20 lyase	contig_19734	P/379	steroid 17-alpha-hydroxylase/17,20 lyase [Strongylocentrotus purpuratus]	1.00E-117	XP_789963.1	TPTWKLHRKLTYSAFRNLATGNNDRFERLVFTMTARVAKILESKGDEEFDPKRIVSLAVYNFIAQMVFGKQYELDDPELLEWQKLSEEGIEVVGNGLLADFFPIFSYIPTPGMRGLKDLVVRYYKLIDKELDRHKAYYNPRDEPKDFIEMLYHARQTMIDEGEEGIDQISESHIRQTVMDIFSAGTDTSIFTMHWAIACMVDFPEVQEKVQKEIDEVIGRDRNPRMNDRGSVPYTECALYEVFRFSSIAPIALPHATTTDVTFRGYHIPKGTWVLPNIYSMHFDKKLWGDPENFRPEHFMDETGSVRQHPEGFLPFSTGRRVCLGESVAKAELFLIFTWLFQNFTFRKPAGKEEVMYARVDKSELNPILKSYDIIVSKR

inactive hydroxysteroid dehydrogenase-like protein	contig_48833	P/108	inactive hydroxysteroid dehydrogenase-like protein 1-like [Saccoglossus kowalevskii]	8.00E-39	XP_002733500.1	NINVGAATMMTSIVLPKMVERKKGAIVNISASASIFPNPQLAVYAACKTYLDFFSRALQYECRKTGIFVQSLMPSYVATKMTDISIQGGKKMSLLMPSASEYARHAVS
hydroxysteroid dehydrogenase protein 2	contig_6984	F/415	hydroxysteroid dehydrogenase protein 2 [Daphnia magna]	3.00E-165	KZS05818.1	MAGQLAGKTLFISGASRGIGLAIAERAAKDGANIVIAAKTATPHPKLPGTIYTAAESIEKAGGRCLPCVVDIRDESAVKAAVNETVAKFGGIDVLVNNASAINLSGTEALDMKRYDLMQSINARGTYLCSKVCVPHLKKSSNPHILNLSPPLLMDAKWFKNHVAYTMAKYGMSMCVLGMAEEFKEVGIAVNALWPKTAIYTAAMEMLGGGEHIKASCRTADICADAAYSIITKDSRSFTGNFCVDEDILRKDGVTDFEKYACQPGHPLQPDFFLDFDPNDPTFQGDVSQIYSEGGGSKSTPGLGSEVKGMFDLIQNQLNEKVVQATGAVFQFELAGQEKGTWHLDLKSGAGAAGPGASSGEPDVIFKMDSKDFVKMFKGELKATSAFMSGKLKIKGDIGKAMKLEKLMGNVKSKM
estrogen sulfotransferase type 1	contig_63795	P/93	estrogen sulfotransferase [Strongylocentrotus purpuratus]	5.00E-11	XP_003726411.2	VIATYSKSGTHWIYEIVQLIRANGYFDKIDRSQMLASTIEMFNIPEMRRPTYEIVHEQKSPRVITTHLPWQFLPKQLTEQKKGKIVYLMRNPK
estrogen sulfotransferase type 2	contig_71705	P/88	estrogen sulfotransferase-like [Strongylocentrotus purpuratus] □	2.00E-21	XP_793921.2	LSQPETLDALQTFEVRDDDVYIVTYPKSGTHWIGEIVALVMEDGVVEKVNRELMATALEITLAPSPEKVKQSTPGYKIYEKLPSPRLM
NAD(P) dependent steroid dehydrogenase-like	contig_64252	P/136	NAD(P) dependent steroid dehydrogenase-like [Apostichopus japonicus]	2.00E-69	PIK44317.1	FTYVENVVHGHILAEEKLKPKSRVTGRAYNITNGEPVPFWDFIGRVLTGLNYSAPKISLPFALVFYIALFLQFLCNILKPIIEINPSLTPAKVTLSATVHYYSIKNANKDLGYQPIVSLDEGLQKTLESFQDLKRS
cytochrome P450 4V2 isoform 1	contig_68628	P/216	cytochrome P450 4V2 [Strongylocentrotus purpuratus]	7.00E-86	XP_799260.2	RRLAFLDLLIEVQKQDPSFTDEGIQEEVDTFMFEGHDTVSTSLTMAMYLIGIHPEVQKKIQEELEKVFGNDRERHVTYEDLQQLEYLSCVMKESQRFLSTVPIVGRDLAEDTKIGGIDVPKGAHVALGIYSLHRDPNQFPKPDKFDPDRFLPSNSEGQHNFAFIPFSGGHRNCIGQKFAVMEQKVILATLLRRIEIKSLQKIDEMRFATELVLRPV

cytochrome P450 4V2 isoform 2	contig_41285	P/202	putative cytochrome P450 4V2 [Apostichopus japonicus]	8.00E-97	PIK50796.1	GAQTGNNKEYVQAIIAMSDLIQERQKYPWLWIDALYGLLPSGKKHNRYLEILHKMTKSVIYERLGEKHQMNGTKETSKASNNGSVDKRRKMAFLDLLLEVHEEDSTFTIDDVREEVDTFMFEGHDTTAAAISWVLFMLGHHPEVQEKVHKELDEVFGSDKDRYVTSEDIQKLQYLGCVIKETLRMYPSVPLIGRQLEEDAVL
cytochrome P450 10	contig_59906, 27680	P/364	PREDICTED: cytochrome P450 10 [Strongylocentrotus purpuratus]	7.00E-67	XP_003727902.1	THVHIIEPYDVRELIRLEGVTPRRITLDPLVAYRKLRKRNIGTSNLQGEEWRRLRQPFNHLMFKPGQINTYIPKVEECTNDFCTLMRELRTQDGEVPDYMRQVQRWTLETLADIVLNTRIGCVDKYPSKLAERAINSAMHFTTGLGDLMYSFPLYKFGIYTETWKNFATAQDFFFEFVQPYVQDAMESMKKDDERATKLGSEYFRMERDATFLESLLAMHMDKDLEAISDLLNDLLLGAIDTISSMFAFVVYNLSRNPDCQERVYGEINRVLPPGTPITPEALQMLPYLKAFVKESTRLFPTVDGTSRILDTDITLSGYNIPANTIIRIHSVAGLMSEYVPEPELFKPERWLRSSDSSIMASP
cytochrome P450 3A24	contig_4256	P/464	putative cytochrome P450 3A24 [Apostichopus japonicus]	0.00E+00	PIK42887.1	FHKFIKQGFVQALQGMRERHGPIYGAYIGSTPATILADADILKEVLIRNFSHFHNRRGTKIDDYPLNKALTRLEGARWKFVRNTITPAFSASKMKPISVLTNRCCDNLIVHMKTLIATNPEFEVKGVYGNMTMDTIAQSAFGLEIDSQSNPNSPFVKHAMAIMNMKLTSPRILMAFWFPQLAPIFNYFRIGMIPNIHKQFFIDVTSKAVSLRKDPKNKRLDILQLMVDAQENPDKSESEEIPDLAGYKEKDEMKKELTTEELMAQAVVFFLAGFETTNIALSFMSYLLATHPEIQEKLYQEIQDVSSSAESFSYDVVNKMEYLDMVVSEALRLYPPGSVADRVCTEDITINDVKFSKGDSIFYNIYSLHHDSRYWEDPQKFDPERFSKENKDKIHPFAYLPFGTGPRNCIGMRFAVLVVKTALARVVQKFKFEPCEKTQIPVKLGKRSIMPEIGITLRVIERE

S2. List of amino acid sequences and their accession number for gene characterization and phylogenetic tree analysis.

StAR

>Hsc_StAR[*Holothuria scabra*]
MADTEQKVEETPAAEEPKKPAEEEKPVENGEKTEEAVADGDAKTEEAKEEGGEAKEEAVENHENGKEGEEEEEEETPAPSLEAPTENPF
TDEDVQQLLDMESGEEGWDKVKEKKGVLVYRKAEKNSSPLIKGVLEYSGIPTEKILEFYTDFEVRRKWNKKTINIEVLDEKDDFKIIHS
IFKMPTGCDNRDAVQCTMVRSEEDGSRHVILYKSCNHPSKPPKKSFPIRADVQLMGAVIRPKEGEEGVTTVTFINQVNLKGWMPKAFVN
KVTVGFPVALYDDLTLYWKKITEPPKSPEKKDKKNKEKNGEENGEAGEEEKNDEKKEEEEEEENKEADVETEKKDEGAAAEGAAEAAGEG
APVGEGDGGKLDTADIPAADEEEAK

>Hsa_StAR[*Homo sapiens*; AAB88174.1]
MLLATFKLCAGSSYRHMRNMKGLRQQAVMAISQELNRRALGGPTPSTWINQVRRRSSLLGSRLEETLYSDQELAYLQQGEEAMQKALGI
LSNQEGWKKESQQDNGDKVMSKVVPDVGKVFRLEVVVDQPMERLYEELVERMEAMGEWNPNVKEIKVLQKIGKDTFITHELAAEAAGNL
VGPRDFVSVRCAKRRGSTCVLAGMATDFGNMPEQKGVIRAEHGPTCMVLHPLAGSPSKTKLTWLLSIDLKGWLPKSIINQVLSQTQVDF
ANHLRKRLESHPASEARC

>Rno_StAR[*Rattus norvegicus*; AAU84904.1]
MFLATFKLCAGSSYRHMRNMKGLRHQAVLAIGQELNRRALGDPSPGWMGQVRRRSSLLGSQLEATLYSDQELSYIQQGEEAMQKALGIL
NNQEGWKKESQQENGDEVLSKVVPGVGKVFRLEVLLDQPMDRLYEELVDRMEAMGEWNPNVKEIKVLKKIGKDTVITHELRAAAAGNLV
GPRDFVSVRCTKRRGSTCVLAGMATHFGEMPEQSGVIRAEHGPTCMVLHPLAGSPSKTKLTWLLSIDLKGWLPKTIINQVLSQTQIEFA
SHLRKRLESSPASEAQC

>Atr_StAR[*Acipenser transmontanus*;ACI23577.1]
MLPATFKLCAGISYRHLRNMTGLQKTAVVAINHELKRFAGPGPSNWISQVRRKSSLLSSRIEEKPYSEVEMSYLKQGEEALQKSISILS
DQDNWKTEIVAANGDKVLSKVLPDIGKVFKLEVVLDQYPDRLYGELVDNMEQMGDWNPNVKQVKILQRIGQDTMVTHEMAAETAGNIVG
PRDFVSVRCAKRRGSTCFLAGMSTRYTGMPEQKGFIRAENGPTCIVLRPNAEDPTKTKFTWLLSIDLKGWLPKTIINQVLSQTQMDFAQ
HLRDRMETIALPC

>Xla_StAR[*Xenopus laevis*; AAG28595.1]
RPGRLLPATFKLCAGISYRHLRNMTGLRRTAAVALTHELEKLALVGPGPGKWINQIRRKSILLSSRLEEKTLNDVEMSYIKQGEEALKK
SLNILGDQDGWKTEIVMENGDKVLSKVLPDIGKVFKLEAVVEKPLDNVYGELVDNMEKMGEWNPNVKEVKILQKIGKDTVITHEKAAET
PGNIVGARDFVSVRCSKRRGSTCILAGMSTRFGGMPEQKGFVRGENGPTCMVLRPLAEDVSKTKLTWLLSIDLKGWLPKSIINQVLSQT
QVDFAKHLRSRMASSSTALSLC

>Dre_StAR[*Danio rerio;* AAG28593.1]
MLPATFKLCAGISYRHMRNMTGLRKNAMIAIHHELNKLSGPGASTWINHIRRRSSLLSSPIAEETYSEADQCYVQQGQEALQKSISILE
DQDGWQTEIESINGEKVMSKVLPGIGKVFKLEVTLEQQTGDLYDELVDNMEQMGEWNPNVKQVKILQKIGQETMITHEISAETPGNVVG
PRDFVNVRHAKRRGSTCFLAGMSTQHPGMPEQKGFVRAENGPTCIVMRPSADDPNKTKFTWLLSLDLKGWIPKTVINRVLSQTQVDFVN
HLRDRMASGGGIDAAIAC

>Sko_StAR[*Saccoglossus kowalevskii;* XP_002740722.1]
MSDETQTAVVTEPKPEENTEAPPKEDKPADEAKVSDATKKDAVAAAPVLVAPTESPFGEDDFEHILQIEASEGAENGWESAKKSDAVKI
FRKKDKNSEINLIKSYLTLPGIPADKAMELLTNYEERKKWDKGHKIDVLDEKSDFCILYSVFKMPAACTGRDFVIASTKRSDEDNKRHI
ILYKDCVHPDKPPNNSLIRGETIVNGFIVRQDENDEKSSRLTVLQQLEMKGWIPKFLLNRLTLARPIGIRDDLNAYWKEINKPKENGPT
DKKEEKKEEGEAEKTEDKEEEPKEENTEEKKE

>Apl_StAR[*Acanthaster planci;* XP_022091858.1]
MSETEQKPQTEEQPQEGDAPAEATEDQPAKEEQTEGTGEVGADGEAAKEEGGAEQAASAEKEESKPAVAAQEASPPSLEAPQENPFSED
DLEQILQLEASEGEDKGWEKSKKIKGVTVFKKPEKNGVPLIKAYMEFSGIPCDKVLEFFTDAELRKKWSRKTPNYEILEERDDFKIVYT
VIKMPMACDDRDIVQCMQVRSDEEQKRHVILYKSCNHPNKPPGKGPIRADVGIMGLVIRPKDGDETSTKVTWLSQIGLKGWALKMIVSR
VTVGYPVSLCDDLQQYWKKTTAPKEPEKKEENETPKEEKTDENAEPKQEEGKTEEAAGDAPVENNVAAPVTADDEDIPAADEEEKGEEK
AGEEA

>Spu_StAR[*Strongylocentrotus purpuratus;* XP_011660912.1]
MDFTGIPVDKVLEFFTDPEQRRKWNKKSPTFEVLEEKEDFKVVYTVFKMPTACDDRDVVQCCVVRSDEEPIRHTILYKSCNHPSKPPTS
KGHIRADVGVMGIVIRPKEGDEVATHVTWVGQVNLKGWMPKMMVNKVTVGYPVSLYDDINTYWKKLTGQDKKEKKDGKAEGEEENKEEE
GEAKPDEEVPAENGEAEPEKAEEKAEEKAEETNAEEKKEEAASEDKPASDEKPAEEGAATETAE

>Aja_StAR[*Apostichopus japonicas;* PIK39463.1]
MPTGCDNRDAVQCTMVRSEEDGSRHVILYKSCNHPNKPPKKSFPIRADVQLMGAIIRPKEGEEGVTTVTFVNQVNLKGWMPKAFVNKIT
VGFPVALYDDLTLYWKKITEPPKSPEKKEKKNKEKNGEENGEAAEEEKKEDKKEDGGEDEENKDADVKTEKKEEADEKPAEGATEEAAA
AAEGAPVGEGDGGKLDTADIPAADEEEAK

>Pva_StAR[*Penaeus vannamei;* XP_027225582.1]
MNRLGQLPSLGTSGSINATPADFLLANYVSGMQINGRMSTVRRFFCLFVTFDLLFTCLFWIICILINGENLVKALEEEVEHYSIKTSIF
DIVMTAAIRFTVLLLFYALLHINHWWLVALTTAGSCAFLIAKTLLYTWQTENSRPFEVMLVLISFLIAWAEAWFIDFRVLPQEARARSI
LEAAAGSSEHMENDPLLPQLERVRDYLSHYTETVGDFYSPMESPLGSDDDDDGFTRTDYRKLSPEEKQMQASGYDALQKAWETVNSPGW
KVEKETSHGDTVSSKVGPKGSKIYKLVGMVEVEPKTLFNEMLFNFENIPTWNKAITLGRVVQTIDSCTDVVYQVAAEGPGGVVSARDFV

NVRHWKKIGDCWVSANVSVVHKDEPPQKSIVRGENGPGCWVFSPVAGAPKKCSFQWLLDTDLKGWIPQYVIDQALTFAMTDFLQCLRNY
APHISASKCERYSAR

>Pmo_StAR[*Penaeus monodon;*ALO17561.1]
MNRLGHLPSLGASGSINATPADFLLANYVSGMQINGRMSTVRRFFCLFVTFDLLFTCLFWIICILINGENLVKALEEEVEHYSIKTSIF
DIVMTAAVRFTVLLLFYALLHINHWWLVALTTAGSCAFLIAKTLLYTWQTENSRPFEVMLVLISFLIAWAEAWFIDFRVLPQEARARSI
LEAAAGSSEHMENDPLLPQLERVRDYLSHYTETIGDFYSPMESPLGSDDDDDGFTRTDYRKLSPEEKQMQASGYEALQKAWETVNSPGW
KVEKETSHGDTVSSKVGPKGSKIYKLVGTVEVDPKTLFNEMLFNFENIPTWNKAITLGRVVQTIDSCTDVVYQVAAEGPGGVVSARDFV
NVRHWKKIGDSWVSANISVVHKDEPPQKSIVRGENGPGCWVFSPVEGALKKCSFQWLLDTDLKGWIPQYVIDQALTFAMTDFLQCLRNH
APYIAASKSERYSAR

>Dpu_StAR[*Daphnia pulex;* EFX88733.1]
MSKESRNRSDQAREVAEIFISNSADHSSQSLTRHHTINTMPNYIHESYYHNEDYLRETLSAGSPRDGRISAVRRFFCLFVTFDFLFTTL
MWIICLVISGENIQQMLIDQIVHYTIHGSLFDIVMASAGRFVILLLFYALLCINHWWVIAITTAGSCGFLIIKVFFFEWAKATNYTLDV
FLVLSSFVLSWGEAWFLDFRVLPQESLARDFFRAISSDDERAPLLQNQAPSVRSHNLDDFYSPIDSPQGMYSLFFPDEELARKAKEAYE
EALSTINSDGWKVEKQTPEGDVVHSKFIRRNHKIFRLTGTVEIPPKLLFDELDRNIENVPSWNPTLIECRNLKTVNDKVSVSYQLAAEG
GGGIISSRDFINLRYCENREGVYICAGTSVVFPDMPAQKGYVRGENGPGCWVMTPIPNEPNKCVFQWLLDTNLKGWIPQSIIDTALSFA
MCDYIRYVRTFADNLRQEGKF

>Ofu_StAR[*Ostrinia furnacalis;* XP_028156947.1]
MGGAGWARGEARVADDSDFQTLKNLLSSENGWTLEYERDGVKVWAEDAAHGALRTVKVVAEFSDVEPDALYDVLHDPEYRSVWDTHMLA
AEDAGHINVNNDVGYYAMSCPAPLKNRDFVLQRSWLDTGDEKMILNHSVFHKDYPPRKGYVRALSLLTGFVVRTRPEGGSWLGYVSRSD
PRGALPAWLVNRVTAQLAPRLVNQLHMAARKYPGWKSLTDLPYHKPWRHPEQVPPYRISLADCVDPDAPPVPEEPKPDTSKGKLEVPQA
KEIESHSVEDLGDISSEFSVEVEEDLSELTLDSTKKKGKFYKLVKSMKNRRKSVQVAKSADNLMEGKPEEKQEKKKGFKFHRRFSLSRG
RED

>Vta_StAR[*Vanessa tameamea;* XP_026484400.1]
MGGAGWTREARVADDTDFETLKHLLGSDDGWNLEYEKDGVKVWAEDAAHGALRTVKVVAEFEDVEPDALYDVLHDPEYRSVWDTHMLAA
EDAGHINVNNDVGYYAMSCPAPLKNRDFVLQRSWLDTGDEKMILNHSVFHKDYPPRKGFVRALSLLTGFVVRRRESPGSWLGYVSRSDP
RGALPAWLVNRVTAQLAPRLVNQLHTAARRYPGWKALTDTPYYKPWRHPEQVPPYRINLEDCIDPDAPPPPPEPKPNTSKTKLEVPQVK
ETESHSIEDLGDISSEFSVEVEEFSELSLDPTKKKGKFYKLVKSMKNRRKSVQVAKSADSLVDGKSSEEKHEKRKSFKFHRRFSLSRGR
ED

>Cgi_StAR[*Crassostrea gigas;* XP_011434195.2]
MPIMSINASTSDMRDVSSSRQDKARQAGTALYGSIHNQAVEPRMENESYTYVYGPERISTHRYSPCSTVSSVRRTFCLIALFDLCFCFI
LWVIYTQLTGSETVWEAFEQQVANYQFQTSLFDTVMVAALRFILIELGYALFRLNHWWLIAISTSLTCIFLITKVFLFEFSHDGKAENP
LSYILLLTSFILTWIETWFLDFKVLPTEKKILLRGTHGEANERSGLLGRAEEGGNRVVHDDDKDRYYSPAGSDDEGEDRYRSLPPSRSH
SRKDHEYLKIANETMDMLFELVREGGWITERESQGDMVQSAYIKKLKRKIFKLECTLDISPHELWKDTIDNVNDSPSWNPTLLESKTLL
VVDEKTDISYNIAAEAAGGLVSARDFINLRHWGERDQTFFIAVNKATFPDMPPQKKYVRGESGPCGLVFNRVTGQPNRCKLQWFLNTNL
KGWIPQAVIDQGLTGVLMDYCKYLRQHIQEIKGRSFS

>Mye_StAR[*Mizuhopecten yessoensis;* XP_021369546.1]
MSINTNEADMVRTSTNRGHASKTAAQAIYGSLSSSSSVSAANGSVGHIGRTDNRMCGWFPDSGKMSPVRRTFCLFVTFDLILTFILWVI
YTQLIGDSSVWEAFTKQLAGFTIYSSLFDTVMLAAMRFSFLLLGYALFRLDHWWVIALTTFLTCGYLLAKSFLFDFKENNNNPLSYVVL
IISFVLAWAETWFLDFKVIPVEKKHRDRVNRDHHYASERSRLIGDSEQGVMSGEDNEYYSPLVTPDGSDDEGEHRGGFQTPRRGHSRQE
QDFVKQARETMDITLEMLNLEDGWKYQAGNDLEQGVVHSMFFKQYNRKVFRLQAIMNLEPKRIWEELAYGTNSSPQWNPTLTECRTLET
IDDNTEISYSIAAEAAGGLVTSRDFVNVRCWGVRDGVFLSCASGTTYPDMPPQKKYVRGENGPGGLILKAMKDDPNKCELTWFLNSSLK
GWIPQSAVEQGLSGFLLDYLKYLSQRLEEIRNQEGS

>Aca_StAR[*Aplysia californica;* XP_005099198.1]
MPAAPIMSINSDVSPLPASSSAREEQAKVAGHVIYNSVHGAADNTRIPPPVSAPVLPSFSDPDMGGPSGPAPLTASLAADPMSEGRMSS
VRRTFCLFVLFDLILMFILWVIYAQLIGDTGYKAFEKEVQNYTFASSLFDSVMLSAVRFTFLLLAYALYRSGHWWMVAFTTALTCGVLI
AKIFLFDFEGSKSNNNPLSYCIIIISFVLAWVETWFLDFKVLPHEKKLMSRLAASTGRGYGSTYTSQGYRTFSNDDMQSVITEDNQFYS
PLDSPEGSDTESDVKESRGAVGGAVAARGLSRQENDYVRLARHSWEVLWTYITSPESDWKSESGSSIEAGVVHSKKVKGVGKVFRLTTL
IEWSPKATYEAVNFQPETQPQWNSALKESQVLQMVDDNTDILYNIAAEVAGGVITSRDFVSLRTWGQREGMYISAGMGVNHPDKPPQKA
YVRGKNGVGGWVYKPVEGEPNKCLFLWFMNTDIGGWFPQRLIDVNMAKVLMDFNRDLQAHVRELKSSRSEP

>Ovu_StAR[*Octopus vulgaris;* XP_029633644.1]
MSIERSIHGLVKQKKQPEEASTTVYARAISEHSATSMSNFATTSVEAEVGAEGPDGKMSPVRRTFCLIATFDIIFTVVIWIIYTQILSA
SKTDSYKNEVLNYDIHYSMFDIVMSAAVRFVLLILAYALFRIRHWWMVAIATFLTCTFLIAKCLVFDFNYASNSLTYALLLVSFVICWI
ETWFLDFKVLPQEKRYLLRTLIPAVSERTPLLRGNNPENPDAISTTDEFHSPIDSPASSDEEPMEGTNKYSQAVNRQTLIDKTPMDLSY
LKLGKESWEQVYSLYKSESNWVFQCGKDATEGCVLSKKSHVGNKIFFLQAVLNISAKELWEDLVFRTEDLSTWNPTILDSKLIKPLSEN
CDISYYVAAPGAKGLVKSRDFVVVRKWGKRKGLYFSTGMSVKFPNIPPVDKCVRGENGAGGYLVEPLSTEPNKCKFYWFCNTNLKGMLP
QKLVDSALGGMQLDYLKHLRHHVEQLRQYKV

CYP10/CYP11

>Hsc_CYP10[*Holothuria scabra*]
THVHIIEPYDVRELIRLEGVTPRRITLDPLVAYRKLRKRNIGTSNLQGEEWRRLRQPFNHLMFKPGQINTYIPKVEECTNDFCTLMREL
RTQDGEVPDYMRQVQRWTLETLADIVLNTRIGCVDKYPSKLAERAINSAMHFTTGLGDLMYSFPLYKFGIYTETWKNFATAQDFFFEFV
QPYVQDAMESMKKDDERATKLGSEYFRMERDATFLESLLAMHMDKDLEAISDLLNDLLLGAIDTISSMFAFVVYNLSRNPDCQERVYGE
INRVLPPGTPITPEALQMLPYLKAFVKESTRLFPTVDGTSRILDTDITLSGYNIPANTIIRIHSVAGLMSEYVPEPELFKPERWLRSSD
SSIMASP

>Hsa_CYP11 [*Homo sapiens;* AAA52162.1]
MLAKGLPPRSVLVKGYQTFLSAPREGLGRLRVPTGEGAGISTRSPRPFNEIPSPGDNGWLNLYHFWRETGTHKVHLHHVQNFQKYGPIY
REKLGNVESVYVIDPEDVALLFKSEGPNPERFLIPPWVAYHQYYQRPIGVLLKKSAAWKKDRVALNQEVMAPEATKNFLPLLDAVSRDF
VSVLHRRIKKAGSGNYSGDISDDLFRFAFESITNVIFGERQGMLEEVVNPEAQRFIDAIYQMFHTSVPMLNLPPDLFRLFRTKTWKDHV
AAWDVIFSKADIYTQNFYWELRQKGSVHHDYRGMLYRLLGDSKMSFEDIKANVTEMLAGGVDTTSMTLQWHLYEMARNLKVQDMLRAEV
LAARHQAQGDMATMLQLVPLLKASIKETLRLHPISVTLQRYLVNDLVLRDYMIPAKTLVQVAIYALGREPTFFFDPENFDPTRWLSKDK
NITYFRNLGFGWGVRQCLGRRIAELEMTIFLINMLENFRVEIQHLSDVGTTFNLILMPEKPISFTFWPFNQEATQQ

>Mmu_CYP11 [*Macaca mulatta;* EHH27471.1]
MLAKGLPPRSVLVKGCQTFLSAPKERLGHLRVPTSEGAGISTRSPRPFNEIPSPGDNGWLNLYHFWRETGTHKVHLHHVQNFQKYGPIY
REKLGNVESVYVIDPEDVALLFKSEGPNPERFLIPPWVAYHQYYQRPIGVLLKKSAAWKKDRVALNQEVMAPETTKNFLPLLDAVSRDF
VSVLHRRIKKAGSGNFSGDISDDLFRFAFESITNVIFGERQGMLEEVVNPEAQRFIDAIYQMFHTSVPMLNLPLDLFRLFRTKTWKDHV
AAWDVIFSKADMYTENFHWELRQKGNVHHDYRGILYRLLGDSKMSFEDIKANVTEMLAGGVDTTSMTLQWHLYEMARNLKVQDMLRAEV
LAARRQAQGDMATMLQLVPLLKASIKETLRLHPISVTLQRYLVNDLVLRGYMIPAKTLVQVAIYALGREPTFFFDPENFDPTRWLSKDK
NITYFRNLGFGWGVRQCLGRRIAELEMTIFLINMLENFRVEIQHLSDVGTTFNLILMPEKPISFTFWPFNQEATQE

>Rno_CYP11 [*Rattus norvegicus;* NP_058982.1]
MLAKGLCLRSVLVKSCQPFLSPVWQGPGLATGNGAGISSTNSPRSFNEIPSPGDNGWINLYHFLRENGTHRIHYHHMQNFQKYGPIYRE
KLGNMESVYILDPKDAATLFSCEGPNPERYLVPPWVAYHQYYQRPIGVLFKSSDAWRKDRIVLNQEVMAPDSIKNFVPLLEGVAQDFIK
VLHRRIKQQNSGKFSGDISDDLFRFAFESITSVVFGERLGMLEEIVDPESQRFIDAVYQMFHTSVPMLNMPPDLFRLFRTKTWKDHAAA
WDVIFSKADEYTQNFYWDLRQKRDFSKYPGVLYSLLGGNKLPFKNIQANITEMLAGGVDTTSMTLQWNLYEMAHNLKVQEMLRAEVLAA
RRQAQGDMAKMVQLVPLLKASIKETLRLHPISVTLQRYIVNDLVLRNYKIPAKTLVQVASYAMGRESSFFPNPNKFDPTRWLEKSQNTT
HFRYLGFGWGVRQCLGRRIAELEMTIFLINVLENFRIEVQSIRDVGTKFNLILMPEKPIFFNFQPLKQDLGSTMPRKGDTV

>Omy_CYP11 [*Oncorhynchus mykiss;* AAB25804.1]
MMVSWSVCRSSLALPACGLPSARHNSSMPVVRQALSPDNSSTVQNFSEIPGLWRNGLANLYSFWKLDGFRNIHRVMVHNFNTFGPIYRE
KIGYYDSVNIIKPEMPAILFKAEGHYPKRLTVEAWTSYRDYRNRKYGVLLKNGEDWRSNRVILNREVISPKVLGNFVPLLDEVGQDFVA
RVHKKIERSGQDKWTTDLSQELFKYALESVGSVLYGERLGLMLDYINPEAQHFIDCISLMFKTTSPMLYIPPAMLRRVGAKIWRDHVEA
WDGIFNQADRCIQNIYRTMRQDTNTHGKYPGVLASLLMLDKLSIEDIKASVTELMAGGVDTTSITLLWTLYELARHPDLQEELRAEVAV
ARQSTQGDMLQMLKMIPLVKGALKETLRLHPVAVSLQRYITEEIVIQNYHIPCGTLVQLGLYAMGRDPDVFPRPEKYLPSRWLRTENQY
FRSLGFGFGPRQCLGRRIAETEMQLFLIHMLENFRVDKQRQVEVHSTFELILLPEKPILLTLKPLKSGQ

>Bfl_CYP10[*Branchiostoma floridae;* XP_002596632.1]
KTYGKIYREKLGPGREMVFVCDPRDIGTVFRSDGRLPQRPPVNSLATYRKMRKKPLGLGNLMGEDWHRVRSSVNKEMMRPKSVGAYATM
QDDVSREMAEQIQTVVRKGDSGGQVDNFMNLMHKWGLESLSLVILGKRLDCLTLDQLAEDSDAQRMISAVLEFFLYFGKLEMSLPLYKY
FNTPAWKRFVRALDTMNRYAICPIQERILTELSKLEEPPQETDFLSNLLSQKDMTLDEAVMMAIELLTGAFESTANTLAFNLYCLAKNP
AAQQKLYEEILEVVPPGQPIDDRVLNKMSYLRAVFKETSRLYPTIFFNARTLTRDVVLSGYHVPAKTQIIMANNVISTLPEYYPDPEAY
IPERWLRTESSAANVQAFALLPFGYGARMCVGRFLPVKNRSVS

>Bbe_CYP10[*Branchiostoma belcheri;* XP_019631487.1]
MSLLLKVVRRVVQGSWFRAGIISVAPRTTTTTATVRSQSTGEEPEGSVAYESSARPFEEIPGPKGLPLIGTALDYSPFGQFPIKTKLME
SFKERTKTYGNIYREKIGPMDLVVISDPKEIERVFRNEGRYPERRPLVSVQIYREAKKLPAGLANLTGEEWQRVRSSVQKDMMRPKTVG
AYAALQDDVTRDLVDVIRALTGKEESGGQIHNFINYVYRWALEAISVVVLDKRLGSLTLDDLEPGSDAQLMINGVNDFFRAFVKLELAA
FGLHRYISTPTWRRFEKAIDQWHYVAAKLLKEKLARSTTEDGKPAESDTDFLHSLLSREDVTFEEAMMMAVDLLAAGIDTTGNTLMFNL
FCLAKNPEAQEKLYREIVEVVPPGQTIDDKILNRMHYLRAVVKETFRVYPTVGNNLRTLDRDIVLSGYVVPARTVIFMANDVISSLPEY
YPEPEKFKPERWLRDDGASGGIQPFAMLPFGYGPRMCIGRRFAEQELHLGLIRIVQNFHVGWSGEDMKQENRFINAPDRDSFVFRERF

>Sko_CYP10[*Saccoglossus kowalevskii;* XP_006825134.1]
MFSLRQVKLISECARFQQQAMFIASRCAMATSAALDISDDDNIISEEMVVKKFEDIPGPRGLPFIGSLLDYTIGPYQLDKLHLAAIERF
REYGPIFKETIGENTFINIIEASDVETLFRSSGKNPARPPMAPLQYIRELRKRNIGLASLQGEEWARVRKPVQQVMLKPKNVQSYIPIV
DEVTTEFTESIKRLRREDGEVDNYQNEVYKWALECVGTLALGTRLGCMERNIQSGSDTQKMITATLGFFDTMRDLTFSMPLYKFGIYNK
TWKQFEKHHETFYKIAEKHIKKALDKLKRLSREGRLEEESKECSLLSYLLSKKSLSSEEIAMVPVDIMQGGVDTTSHTAIFIMYQLATN
PDAQEKLHQEVDSVLANSQEMTLESLQNIPYLKACVKETHRMMPTIDGTTRIPDKDIVLSGFHIPAKTVVRTHCIAGLMHEYFDEPHVF
KPERWLRVSSEEIDHHLLLPYGGGTRMCIGRRFAEQELHILIAKLIKNFRIEWHHGPMQQLFRLANAPDVPAQFKFVDREQ

>Spu_CYP10[*Strongylocentrotus purpuratus;* XP_003727902.1]
MLPASVGRTSACRMASMAQQILHRHVGTMVTSEHLEDAKPYDEIPGPKGVPFFGSLFDYTGLGPYKLDKLHEATVDRFRNFGPIFKETL
AGVTHVHIIDPTDVRELFRNEGRSPKRTPIDAMVKYRRTRKRHIGMANTEGEEWQRLRKPVQHLLMKPQSVYAYIPIMEECADDFVTLM
KETQDIESQEVPEFNHKMQRWTLESVCAIVFDTRMGCLNTGLDNNPEALDMIVSVSNFFDSLKDLTFSFPFWKFGITTKAWTKFTEAQD

YFFGMSKKYATIALDRMKSAVGTSTQSESEGTFLECLLARRDFDIDSLCDLMNDLMIAAVETTASTLAFNLYCLAKNPEVQERVFQEIN
QAIPPGTKITAQSLQNLPYLKACIKETVRVFPTVDGTNRIPSREIALAGYRIPPDTIVRIHCIAGLMEEYFPSPEKFKPERWLRGDAEC
ENIDPYLILPFGYGSRMCTGRRMAEQDLYIMLIKIVQNFKIEWHHEEDMELIFRLTNVPDRPAQFRFVSRT

>Apl_CYP10[*Acanthaster planci;* XP_022096144.1]
MFSASQSSTMFQLAKAAVGSRPVQLYLCRMTCTSSPANAAQAGVCPMANNQDMTEATADVALASAPQPRTGSVTGPNALSTARPYEEVP
GPKGLPVLGTLLDYTKLGDYSLEKMHEAIVDRFKKYGPIYKENIAGAEAVHILDPKDVADLFRNEGRTPRRIIMEPMAFYRKIRKKNAG
IANLQGEQWRKWRSALQHIMLKPKSVADWIPNMEACANDFIEMMAARRDDKGEVPDYLGQIHKWALESSFSIVFGKRIGCLDPSTDTNP
DAAKLISSTNDFFTGLCNLTFGFPLYKYGIRTKKWSKFANAQDLLYNTAVKYIHQYIEEMNNRDATSPADGQKDIGDEQATVIDYLLRR
GTLEFEDLCTLTNDLLLAAIDTTSNLMAFCLYCLAKNPEVQERLHEEVNRVLPAGTPATAEALQTIPYLKAVVKETTRVFPPVDGTSRY
SNDDIAVRGYRIPANTLVRVHCLSGQMPQYFKEHHKFKPERWIRGHKEFEDIDPFLVLPFGTGSRMCLGRRLAEQEVYLLLARLVQRFT
IEWHHEDLDYLFRLTNVPSRPLELTFNDRA

>Aja_CYP10[*Apostichopus japonicus;* PIK42398.1]
MISDLLNDLLLGAIDTTSSTFSFIIYNLAKNPECQEKVYEEISRVLSPDEPVTPEVLQMLPYLKACVKESARLFPTIDGTSRILDKNIT
LSGYNIPANSIIRIHAVAGMMSEYVPEPEKFQPERWLRAADRSILANPYLTITFGVGFAVAWGNDSLNSKSTRDLSSFCKISASNGTEK
ISLWTLRLPVDQAVQCNQRSLTGHEL

>Dpu_CYP10[*Daphnia pulex;* EFX79019.1]
MKDPRISKIIDPTCETDNVKTFEEVPGLRVLPIIGTTWGFFPHIGDGIPISRMLELQKLRFKQFGYIWRDIVPGRPPIVYTADPEDVEK
LFKTEGKHPVRPGMETIKAYRAQRIQDFSSAGILLSSGEEWWNTRSKAQHTFLKPKNVTHYITELSEIADDFVNRIRLIRPENNEMTPD
FLNEMYRWALETVGVVGLNTRLGCLQQDLAPDSEAQKMIDAANFSFSAINELEHKFPFWKFFVTPMLRKLYDAQDFFTETTIKYINQTV
EALKGSSFDSYLSIIEQLLVNGMDPSDVTTMVIDMLMAGVDTSANTSAFLIYYLAKNPDKQEKLREEIFSVIGPKGSPITSNALNNLPY
LKACIKESFRLMPAANANARITDKDLVLSGYSIPKGSLVVALHQLMSHLDENFPDAQKFIPERWIKGDAQESKTHHPYVVMPFGSGTRM
CIGRKLAELEIDQLIIKLLQNFKIEYHHEDMSCFMRVTNMPDQPLLFKFIDL

>Dma_CYP10[*Daphnia magna;* KZS08529.1]
MSKRMSATEFDNAKPFDQVPGLRVLPIIGTTWGMFPHIGDGIPISRMLELQKLRFKQFGYIWRDIVPGRPPIVYTARPEDVEKLFKTEG
KHPVRPGMDTIKAYRAQRIKDFTSAGILLSSGDAWWQTRSKTQQTFLKPKNATHYIPVLNDIAEDFIDRIRLIRPENNEMKPDFINDMY
RWALESVGVVGLNTRLGCLRPDLAPDSEAQKLINAANFSFDAINDLEHNFPFWKFFVTPMLQKLYDAQDFLTDTTIKYISQTVEALKGS
SFDSYLSILEQLLVRGMEPSDVVTMVIDMLMAGVDTSANTSAFLLYYLARNPEKQEKLRQEILSVVGPKGSTATASALNSLPYLKACIK
ESFRLMPAANANARITDKDLVLSGYIIPKGTLVAALHQLMGRLDENFPEAEKFIPERWIKGDLQESHHHPYVTLPFGCGTRMCIGRKIA
ELEIQQLTIKMLQNFKVEYDHEDMSCFMRMTNMPDKPLLFKFIDL

>Lgi_CYP10[*Lottia gigantea;* XP_009056782.1]
MSSLIPKTFAKIAVTALPEIATKRVVSTSVAHKQSARVIEVDPLTLGTCPFRSTFEKVESIVNPIEPKVEPQELTKETGIKSFEHVPGP
KGLPILGSLLDYFKKDGLRFDKMFEAFKVRSIEFGPVYKESIGPIDTVVISDPAEYAKVIRADGKFPNRKEMEPMAFYREQKGIGLGLV
NSQGEEWHRQRSAVIKKMLMLNEVQDFSQPMNVVANDFITHLSSSRDASGEIQQLDKQIFKWAMESIGTFLFEERIGCLNEKPTELAQE
FIDTLQKYFRLMQQLMYNLPMYKVFRTKKWQEFEKLSDRIMEVGREFVDKKIEKLQNADPSTSQEKGAFLTHLISQKSLSTSDVTSNAV
DLLSAAVETTSNASLWCLYNLATNPEAQQTMFNEINTVLPNKEDVTPQALSKLPYVKAVLKETFRKYPITYATSRFLPENLEVGGYNIP
AGTHVQANLYGMYSDAKIFPEPEKFKPERWLRESKMDSQTKSLSNLIWGHGARMCIGRRFAEQEMHIMLTKIIQNFKLEYHHEPVEPTL
NTVMTPDRPVQIKFVPRS

>Cvi_CYP10[*Crassostrea virginica;* XP_022312941.1]
MAPIQKPQELTNLSQTSSVKPFDSIPGPKGLPVIGTLFDYMKKDGFRFNKLFEAYRQRAQVFGPIFKENIANISTVVITDPTEYNKVVR
ADGKYPKRHQMEPWYHYREQRNRGQGLVDLEGSEWHRVRTVVSKKMLKLKDVQDYCTDMDEVSQDFTTHLSSLRNQHNEIVGIEKELFK
WSMESIGTFLFNSRIGCLRPXPPQETKDFVDHLQGFFKLMQPLMYNIPLYKVFPTKIWXQYVTHADNIFNIGRSLVDKEALRLEQNPPA
EGEKSSFIQYMMSQKSLTENEALSTVVDMLISATETTSSGTLWALYNLAXNPEVQEKLYEDTKRVLPNKETITPEKLSELQYVKAVMKE
TFRLYPITFATTRYLAEDTEVAGYTIPAGTHVQGNLFGMFRDSKLFPEPETFKPERWLKGSQMNQEIKALSNLVWGHGARMCIGRRFAE
QEMHILLTKIVQNFRLEYNHPRWAVLNTVMTPDRPLQISFIPRN

>Mye_CYP10[*Mizuhopecten yessoensis;* XP_021376627.1]
MASTTTKALTKLVKINTPIKRVVSTSTSSRQGAQVLAVGADIAGTCPFRSTFETIDNMMSRPQQTAAIQVETPVIENKVQNYDAIPGPK
GLPLIGTLFDYLKKDGLRFNKMFEAYRTRALEFGPIYKEKIATISTVIISDPDEYSKVIRDEGKYPMRREMEPMAYYRSKNGIGLGLVN
AQGEEWHKYRTVVSKKMLKMKEVLDYCGSMDTVAEDFVQHLKDRSTTKHEVPVLEKEIFKWAMESIGTFLFEERIGCFGSPTPTQAQSF
IDNLQGFFKLMQPLMYNIPLYKVMPTKMWKQYEAYADNVMKIGRSFVDKKAKALEGVVPKENEKVSFLTYLMSQESLTPDDALSTAVDL
LVGATETTSNGSLWVMYCLAKNPTVQEKLFQEIQNVLPYKEAITPETLNKLPYIKAVIKETFRLYPITFATSRFVQKDIEVGGYNLPAG
THVQANLYGMFRDPEYFPDPEEFRPERWLREANMESNIKALSNLVWGHGARMCIGRRFAEQEMHIMLSKMVQNFKIEYHHEDIEPVLNT
VMTPDRPLKLSFVPRESECL

>Ovu_CYP10[*Octopus vulgaris;* XP_029640276.1]
MATFRRLTQQTYKKTVFYSNMRNISTSATGQQRVAELDDTFITECPMRKTLNVLKTYASEERASSNLIGRPFEEIPGPKGLPILGTLLD
YFKKDGLSFTKMFEAYNKRALEYGPIFREQITTVKTVVISDLDEYSKVIKAEGRFPNRREMEPMMYHRYQRGIDLGLINSQGEEWHKYR
SVLSRKLLLSKEIQQHCSAMDAVANDFITRLDLLQQSENGEVHNLEHEIFKWSMESVGTVLFEERIGCLDPEPPKLALDFIANLVGYFK
LMQPLMYNAPIYKVFPTKQWNQFVSFADNVMNIGKTFVERKLNKLQKEIEDGNVNSEETSKFLSYLLTQKSLSLCEATSSAVDLLSGAV
ETTSNSIVWVLYCLAKNPQVQEEIYKEVCKIVGPGEELTPAKLTSIPLVKACLKEALRIYPITFATSRVTNKDIEVCGYHIPAGTHVQC
NVYGLGRNSDVFPQPLEFRPKRWIGNRSDNNGKALYNLTWGHGPRMCIGRRIAEQEIFILLTKIIQNFRLEYHHEDVQPVLNTVMTPDR
PLLLKFVPRDPENQ

>Cgi_CYP10[*Crassostrea gigas;* XP_011439055.2]
MYRSQKRVVSLSSSSPQTIVTNVAPIACPLQGGQDSLDKSFEQNKDQQPLTPSVKAFDNIPGPKGLPIVGTLLDYMKKDGLPFNKLYKV
YRQRSLQYGPVFKEKLANFTTVVISDPAEYNKVVRAEGKYPLRRDVEPWFLYREKRKMGQGLVNSHGPEWHKYRSAASKKMLKMKEVSE
YCKSMDEVADDFIVHISCIRNQQGEVIGIEKECFKWAMESMGTFLFESRLGIFSSNPPHEAQIFIENLQGLFRLMQVLMYSLPIYKIFP
TKDWRQYVNYCDNVFRIGKSYVNKKAAQIRENPAKEGERTPFIEYMMNQESLTEQEALSTCVDLLVGATETTSSGILWALYCLAKNPVA
QEKLYKEIKLVLPNNEAITPEKLSKLQYVKAVVKETFRLYPLTFTTSRYLEEDLEIGGYNLPAGTHVQANMYAMFRNSQYYSEPEVFKP
ERWLKDSGMDNNLKAMSNLVWGHGARMCIGRRFAEQEMYILLTKIVQNYKVEYHHGSVEPLLNMVMSPDRPLQFSFIPRE

>Cte_CYP10[*Capitella teleta;* ELT96518.1]
MKGGQVVRKEKKLKPFKDIPGPKGLPVIGTLLEFTKKDGLKFNKMFEVMTSRSKEFGPVYKERIGMIESVIVSDPHEYAKVIQVDGKHP
HRIELFPMVHYRQKKKMALGTVNAQGEEWYRGRMVLSKVMLKPKEVQDYVIAQSDVGNDFIRHMKSIVSSDGQLISFEREIFKWALESI
CVVLFEERIGCFPNPPTEKAQEFITNLIGFFKYMQPLMYNFPMYKIYPTKTWRQYEKHGDIVVGIGLDMVRKVKKESKSEFLTYLLAQK
SLSPVEANSHAVDLMMGAVETTANSMMWMLYCLSNFPDAQRKLQEEIDKIVPQDERITPEVLSKMKYTKACLKETFRLFPITFATSRMI
HEEIELGGYHIPKGTHCQANLWGMGRDPDYFSDPLTFKPERWLRDIQTLEHHNPHAVLPFGHGARMCIGLRFAEQEIYIAVAKLMKHFT
VKHEGELLPVLNTVMTPDRPVNFRFLLRTKPALSGLGDTKKPIAVELK

<u>CYP17</u>

>Hsc_CYP17[*Holothuria scabra*]
TPTWKLHRKLTYSAFRNLATGNNDRFERLVFTMTARVAKILESKGDEEFDPKRIVSLAVYNFIAQMVFGKQYELDDPELLEWQKLSEEG
IEVVGNGLLADFFPIFSYIPTPGMRGLKDLVVRYYKLIDKELDRHKAYYNPRDEPKDFIEMLYHARQTMIDEGEEGIDQISESHIRQTV
MDIFSAGTDTSIFTMHWAIACMVDFPEVQEKVQKEIDEVIGRDRNPRMNDRGSVPYTECALYEVFRFSSIAPIALPHATTTDVTFRGYH
IPKGTWVLPNIYSMHFDKKLWGDPENFRPEHFMDETGSVRQHPEGFLPFSTGRRVCLGESVAKAELFLIFTWLFQNFTFRKPAGKEEVM
YARVDKSELNPILKSYDIIVSKR

>Hsa_CYP17[*Homo sapiens;* NP_000093.1]
MWELVALLLLTLAYLFWPKRRCPGAKYPKSLLSLPLVGSLPFLPRHGHMHNNFFKLQKKYGPIYSVRMGTKTTVIVGHHQLAKEVLIKK
GKDFSGRPQMATLDIASNNRKGIAFADSGAHWQLHRRLAMATFALFKDGDQKLEKIICQEISTLCDMLATHNGQSIDISFPVFVAVTNV
ISLICFNTSYKNGDPELNVIQNYNEGIIDNLSKDSLVDLVPWLKIFPNKTLEKLKSHVKIRNDLLNKILENYKEKFRSDSITNMLDTLM
QAKMNSDNGNAGPDQDSELLSDNHILTTIGDIFGAGVETTTSVVKWTLAFLLHNPQVKKKLYEEIDQNVGFSRTPTISDRNRLLLLEAT
IREVLRLRPVAPMLIPHKANVDSSIGEFAVDKGTEVIINLWALHHNEKEWHQPDQFMPERFLNPAGTQLISPSVSYLPFGAGPRSCIGE
ILARQELFLIMAWLLQRFDLEVPDDGQLPSLEGIPKVVFLIDSFKVKIKVRQAWREAQAEGST

>Rno_CYP17[*Rattus norvegicus;* NP_036885.1]
MWELVGLLLLILAYFFWVKSKTPGAKLPRSLPSLPLVGSLPFLPRRGHMHVNFFKLQEKYGPIYSLRLGTTTTVIIGHYQLAREVLIKK
GKEFSGRPQMVTQSLLSDQGKGVAFADAGSSWHLHRKLVFSTFSLFKDGQKLEKLICQEAKSLCDMMLAHDKESIDLSTPIFMSVTNII
CAICFNISYEKNDPKLTAIKTFTEGIVDATGDRNLVDIFPWLTIFPNKGLEVIKGYAKVRNEVLTGIFEKCREKFDSQSISSLTDILIQ
AKMNSDNNNSCEGRDPDVFSDRHILATVGDIFGAGIETTTTVLKWILAFLVHNPEVKKKIQKEIDQYVGFSRTPTFNDRSHLLMLEATI
REVLRIRPVAPMLIPHKANVDSSIGEFTVPKDTHVVVNLWALHHDENEWDQPDQFMPERFLDPTGSHLITPTQSYLPFGAGPRSCIGEA
LARQELFVFTALLLQRFDLDVSDDKQLPRLEGDPKVVFLIDPFKVKITVRQAWMDAQAEVST

>Ssc_CYP17[*Sus scrofa;* NP_999593.1]
MWVLLVFFLLTLTYLFWPKTKGSGAKYPRSLPVLPVVGSLPFLPRRGHQHMNFFKLQDKYGPIFSFRLGSKTTVVIGDHQLAKEVLLKK
GKEFSGRPRVMTLDILSDNQKGIAFADHGTSWQLHRKLALSTFSLFKGGNLKLENIINQEIKVLCDFLATRNGESIDLAQPLSLAMTNI
VSFICFNFSFKKGDPALQAIVNFNDGILDAVGKEILYDMFPGIRILPSQTLENMKQCVRMRNELLREILENRKENYSRNSITNLLDIMI
QAKTNAESNTGGPDHNLKLLSDRHMLATVADIFGAGVETSASVVKWIVAFLLHYPLLRKKIQDAIDQNIGFNRAPSISDRNQLVLLEAT
IREVLRFRPVSPTLIPHRAIIDSSIGEFTIDKDTDVVVNLWALHHNEKEWLRPDLFMPERFLDPTGTQLISPSLSYLPFGAGPRSCVGE
MLARQELFLFTAGLLQRFDLELPDDGQLPCLVGNPSLVLQIDPFKVKIKERQAWKEAHTEGSTS

>Xla_CYP17[*Xenopus laevis;* AAG42003.1]
MISYVAGALLLAFGLALISVWKFAGGKHRGAKYPNSLPCLPFIGSLLHIGNHLPPHILFCKLQEKYGSLYSFRMGSHYIVIVNHHEHAK
EVLLKKGKTFGGRPRAVTTDILTRNAKDIAFANYSPSWKFHRKVVHAALSMFGEGTVAIEKIISREATSLCQSLISFQDNPLDMAPELT
RAVTNVVCALCFNTRYKRCDPEFEEMLAYSKGIVDTVAKDSLVDIFPWLQIFPNKDLDILKRSVAIRDKLLQKKLKEHKEAFCNEEVND
LLDALLKAKLSMENNNSNISQEVGLTDDHLLMTVGDIFVAGVETTTTVLKWTMAYLLHYPEVQTKIQEELDFKVGFGRHPVLSDRRILP
YLDATISEVLRIRPVAPLLIPHVALQESSIAEYTIPQDARVVINLWSLHHDPNEWENPEEFNPERFLDENGNHVYSPSQSYLPFGAGIR
VCLGEALAKMEVFLFLSWILQRFTLELPAGDSLPDLDGKFGVVLQVKKFRVTTKLREAWKNIDLTT

>Dre_CYP17[*Danio rerio;* NP_997971.2]
MAEALILPWLLCLSLFSAVTLAALYLKQKMNGFVPAGNRSPPSLPSLPIIGSLMSLVSDSPPHIFFQDLQKKYGDLYSLMMGSHKLLIV
NNHHHAKEILIKKGKIFAGRPRTVTTDLLTRDGKDIAFADYSSTWKFHRKMVHGALCMFGEGSVSIEKIICREASSMCEVLTESQNSAV
DLGPELTRAVTNVVCALCFNSSYKRGDAEFESMLQYSQGIVDTVAKDSLVDIFPWLQIFPNKDLRILRQCISIRDKLLQKKYEEHKVTY
SDNVQRDLLDALLRAKRSSENNNSSTRDVGLTEDHVLMTVGDIFGAGVETTTTVLKWSIAYLVHNPQVQRKIQEELDSKIGKERHPQLS
DRGNLPYLEATIREVLRIRPVSPLLIPHVALQDSSVGEYTVQKGTRVVINLWSLHHDEKEWKNPELFDPGRFLNEEGDGLCCPSGSYLP
FGAGVRVCLGEALAKMELFLFLAWILQRFTLEMPTGQPLPDLQGKFGVVLQPKKFKVVAKVRADWEKSPLMQHC

>Bbe_CYP17[*Branchiostoma belcheri;* BAF61104.1]
MWLMTITVGVVTLVLVTWLRDYVRRWRMPPGPFFWPIFGNLSLFAGKFYLTFIDLAKKYGDVFSLKQGMTDVVVLNSLDAIREAFVKKG
VEFAGRPTFVSADIFTEGGKDIAFTDYSPTWKLHRKLFHTAIRGYASGENLQNKVHESLEETIAEFSKMEGQAVDLEDYIYKLVYNVIC

SAAFGVRYNMDDKDFNTLMKVSKDSAEVFGEGILADVFPSLRFLPTPGITAIQKIVRPFLDIMQHHLDQHRETFDPNNLRDITDHMIKA
QKDAEEEGMQDINSLTDTHLRQTIADIFGGGTDTTILTLRWAVLFLSAHPEIQEKVAAELDSVVGRDRLPELSDREATPYTEATIHEVM
RMGSIAPSSLPHTTTVDTTLRGHRIPQGTWIMPNLWAVHHDPDTWGDPDVFRPERFLDENGKPIPKPPALMPFSAGRRACPGEAVGKAD
TFLLLGGLVQNFCFSIPEGEGPPDLTPDEKGGGVTSLPSPYKVVMTCRK

>Bfl_CYP17[*Branchiostoma floridae;* XP_002601863.1]
MWLMTITVGLVTLVLATWLRDYVKRWRMPPGPFFWPIIGNIYFFSGRMYTTCTDLAKKYGDVFSLKMGMTDVVVLNSLDTVKEAFVKKG
VDFAGRPKILSMDIFSEGGKDIAFTDYSPTWKLHRKLFHGAIRGYALGENLQTRVHESLEDTIAEFTKMEGQAVNIEDYIYQLVYNVIC
SAAFGVRYTMDDEEFNTLMKINKDTEEIVGQGLLADVYPSLRFLPSPTLTNIRKMTKQYVGIMQRHLEEHRETFDPSNLRDITDHMIKA
QKDAEEEGIQDINSLTDTHLRQTISDIFGGGTDTTIFTLRWAILYLAVHPEIQEKVAAELDSVVGRDRLPELSDREATPYTEATILEVM
RMGSIAPLSLPHATTVDTTLRGYQIPKGTWVVPNLWALHRDPDTWGDPDVFRPERFLDENGKPVPKPAALMPFSAGRRACPGEAVGKAD
TFLLLGGLVQNFSFSIPEGEGPPDLTPDDKAGGETLNTPSARTTVSPDVVKHLR

>Sko_CYP17[*Saccoglossus kowalevskii;* XP_002730850.1]
MIWSIHRPSGMPPGPRGFPIIGSMLSLTDNLHLDFLQMAKEYGDIFTIKIGSHQVVIVNSYELIKETLVKKSTDFAGRPQTFTGNVGSE
GFKDIAFSDYSPSWKLHRKIAHQAIRNYASGDKLEDMMRKDAIPLLTQILEDNEGKVIAPRPLLFLTVNNIVAQFCFGQKYTLDDPELK
AFMDLIDEFANVIGKGLVADVIPWAAIFPTSAARKMDKVNNKYLGMIYKKFYEHRQRFDEDNLNDLIDYLLDAQRQAKEENADNINSLT
DTHLVQTVSDIFGAGIDTTTHTMDWSIIFLTRYPDVQAKVAREIHEVIGRDRLPLISDRPNLPYCDAVIHELMRIRTVVPMSVPHKALV
DSSIGGYLIPKNTWVMVNLWAVQMDEKHWDNPQEFRPERFIDKDGSLKAKQDNFIPFSAGRRVCLGESLAKPEIFLMFTSLYQSFAFSQ
VPGKALPSLKGNVASLVLRAPEYEVVVKKRI

>Apl_CYP17[*Acanthaster planci;* XP_022111252.1]
MAILGDFYVNATTVLVAVCTLAAYALYRSTKRPANFPPGPFAVPLLGNVTAFMSNKTAVEVFSDLHKKYGPVYSLKFANRDVIMLNTVE
VVKEALLKKAVEFAGRPAIYSVEIFTEGYKDIVFSNYGPQWRYQRKLGHTALRHFAKKEQLEKLVYSVTPEIAKVLDGLGDKPFAPKYT
IGQIVYNILASMCFGRQYEFNQPELLQWIDLNVEAQKAFGNGLLPDYFPALSFIPTQSEKKVCKIVGAMLDVFYGELKRHREQFDPDNV
RDFFDSLLLTQKQAIEAGEEEADKITDTHIVQTVSDIFGAGTETTIITLHWAVGLMVENPDIQQKVAMEIDEVLGRDRLPSLDDRASLP
YTEATILEIFRYGTVAPTGLPHAVMEDTTLAGYDIPKGTTVMIDILALHNDTKEWNEPEKFKPERFLDDNGQLLSKLPESFLPFSAGRR
VCLGEDFAKKEIFLLFTWLFSRYTFYKVPGKESESVLTLNRVAALTHQPIDEMEVCVKKQY

>Spu_CYP17[*Strongylocentrotus purpuratus;* XP_789963.1]
MIDLLGAGESGFGQFNALTITATLGVVTATLALWTIMRPNRFPPGPRGLPIVGSIFSLNDSPEVVFGEWAKKYGDIFGFKAGERWMVVL
NRQALIKEAVLKQGVDFAGRPDFYSVNIFTEGFKDIVFSPYSETWKLHRKLAHSALRHFATGKPLQGLISSVYPKVEKKLAMTEGQPID
PKVLITLIMYNVLAQMCFGLSYELEDPNVTQWMDVNNDVNEKLGLGLAADIFSWAKYIPTSGPRMIKEITETMFGFLRSQVDEAREHYD
PENINDFYSLLLKAQEDARKEGENVDKLTDTHIFQTVADIFGAGIQTTVETLYWAMALLVTYPEIQAKIRAEIDDVIGRDRLPTINDRG

NLPYTEASLYEVLRYSSIAPIAVPHATSRDTEFGGYHIPKGTVVFINTHSMHYDPQEWDQPDKFLPEHFMDGSGTVREHPPSFLPFGAG
RRGCLGEAVAKADLFLIFSWFLQNYTFSKVPGKESEDILNMIPQTAFGRLLLSYEIMINKRD

>Aja_CYP17[*Apostichopus japonicus;* PIK40958.1]
MAFESSAVVVNSTSLTLVTITTVLAGMWIWSQQKPTKNFPPGPKGWPLIGNILELARNEKPAFVVFTEYAKEYGDIFSIRVGQRWAVVL
NGAATIKEALLKKGVEFANRPTSVTIDLFTEGGQDIVFGQYSPSWKLHRKLAFSAFRKLATGDNKRFEKLVYSIIPALTANLDSKGSEP
FDTRRVLASSIYNILASLCFGKQYEFDDPELTRLMYLSKEGNDIAGSGLLADYIPIFKYVPTPGQTKLKAIFKDFFSLVNKEVDEHREK
YDGGEPKDFIEMLFQSRQEIKDEGKEDMSLITEAHIRQTVVDVFGAGTDTSIFTLQWCAGLMVQYPEVQAKVAEEVDRVVGRDRLPSLD
DREELVYTTATLYEVMRYSTLVPLAVPHATSADVELGGYTIPKDTWVLVNIYSMHYDEKLWDDPKKFTPEHFLDESGKVRLHPEGYMPF
STGRRVCIGESVAKAELFLLFAWLFQHYKFSKPIGQEEKDFSEGDPQAALNMLKDIEVVVEKRF

>Cgi_CYP17[*Crassostrea gigas;* XP_011454188.1]
MLKLSINIQTILAGMSIGLFVYYVIKRMRYRLPPGPWCIPLIGHYKIYSSPEFHLKTAALAKEYGPIIRLSVGPQTWVVLNDINSVLEA
MVTKKTDFAGRPHITSGDVFTEGGKDITLANYSASWKLHRKIVGKALRNYLQGDLLENMIQDNMNKFLDKMAEEKGPFVFKEYMDLIIF
HQLYTICFGEKRPVDDPEVKRFIKLDNDLLEKFGSGFFEDVFPYLRHIFTTAKWKKMLLLTEEVLNVLRKNLKEHKETFQPGVNRDFTD
SMLIAKQEAKDEGDEAALEILDDTHLVQTISNIFFAGADTTRFLIDWFVYFMTRFPETQAKCQEEIDRVVGSKQLSMKDRGNLDYTEAC
IFETMRISNSVGFGIPHMTVCDSQVGGYDIPKGTTVLINYWALHHDPKQWKDPEQFDPHRFLDENGKMKPTKPDSWLPFSVGRRVCLGE
SLAKSDILLICANLLQRFEISLPEGVKPNLECHMKGFGMEMTSDYKIVVKERSG

>Mye_CYP17[*Mizuhopecten yessoensis;* XP_021361085.1]
MEDIKDQLRDGFQQLKETLGPMHKYVTPTSALVGLGAGLSVYWLIKRSKYNLPPGPTPIPVVGNYSLMGDKDFHASCVKLSHKYGPVIT
IYLGMSPVIIVNTIEAATEVLVKSKADFADRVEFPSGNKFSEGGKDIAFAHYTPTWKLHRKIAGSALRTYLKGGKLEEAIHGSLSQVIE
LFENLKEEPFVPHPYIDLAVFNIIHGLCFHKVYKFDDPRFKRLVDLDNELITTFGSGFLEDLFPFLLKIWPSSRFNKAMQLAEQVLQFL
DDELQEHITSFDEGNIRDFADALILARKEAENDEDTELLDQLTEAHLRQTLNDIFFAGFDTTRFTLQWFFLYMAANPEEQKIAQKEIDT
AVGGDRLPGLGDRNAMPYTEAVLHEVMRIASVAPFGVPHAARNDSKLYEYDIPKGTMVMINHWALHNDPKHWKDPSKFDPKRFLDSDGN
LAPKPVSWLPFSAGRRVCLGESAAKPELHLICATILQQFEIMLPPGVTADFSPDVSGIGGYTANQYKIVVQKRT

>Lgi_CYP17[*Lottia gigantea;* XP_009050593.1]
MLETYLNKIPVSTTTQALLLGVTIGAITYTLFKKKTKIPPGPRPLPLIGNLLLLNSSEPLFKLLFKWRKEYGSIITLYMGNFFKMITLN
NADVVNDALVKKGGDFAGRMHLHSLEVISEGYKSIAMADTTYAWKLQRKLSLQGLRQYLSGNKLEEKLHESVSRTIRLLKEKENEPVSI
LSYISLTVFNLLTGVCFSKSYEMGDKEFIELVSDFDTFSTEFGNGLWEDIVPLLKYWPTKKYNRLMKMFNSFLDVMYKNYHEHVQSFSE
DNLKDFTDFMIYAKKQAAVDDPNIENIITDTHIVQTIADIFGAGVDTSRQTIVWTLYLLAENPEIQKKVQSEIDEILDLNQAPSVKDRS
SLPYTEAVLHESMRICPVAPLSVPHTSQCDTTLGEYEIQKGTTILINTWALHMDPETWGDPEVFRPERFLDEDGSLAPKTMSWLPFSAG
RRNCLGETIARPELHLILAVLLRNFTFDKAVGPSGETWTMEPASGGFTFLPPDNYMKIQSRK

>Cvi_CYP17[*Crassostrea virginica;* XP_022344772.1]
MGILSLNTQTVLAGVCIGLLVYYVIKRMRYRLPPGPWCIPLIGHYSVYSSMEIHKKVRDLSKEYGPVLRLSFGPQMWIFLNDSETVLEA
MVKKKADFAGRPQFSSGDYFTEGGKDIAFSNYSHSWKFHRKAAGKALRTYLQGDLLENMIQDNMNKFLDKMAEEKEPFMFKNIADLMVF
HQIYTICFGEKRPADDPEVKKLLELGNDLLATFNNGLVEDIVPYLKDIYPTARWKKMVALIDQILNVFRGKLQEHKDTFKPDLNRDFID
SMLIAKQEAQAEGDKASLESLDDTHLVQTLSDIFFAGVDSTRFTIDWFVYFMTRFPEIQTECQKEIDRVVGSDQPSMKHRSKLDYTEAC
LFETLRLGSVAGLGVPHMTICDSQVGGYDIPKGTTVLINYWALHHDPKQWPDPTKFDPSRYLDENGKLESTKPASWLPFSAGRRNCLGE
TLAKPEILLMCANLLQRFDIRLPEGVKPHLEHNLKGFGIELPMDYKIVVKERSTN

>Pda_CYP17[*Pocillopora damicornis;* XP_027053398.1]
MILEIAFVFVCVTTIAFFVHRAHAQKSKKLAPGPRPYPLIGNMPRLAGKPGHLAMTELAEEYGKIYTLYLPGGQRCVVINSIDLAREAL
LARRDEFSGRPVTFVGDYLSRGGRNIICADFTQSMILLRKIAHSALRMYGSGLKHLECLISSEVDHMAKRITSQQGKPFDPKKEIQLTI
LNVICAVVYGESYDIEDEEFLRIIQYNDDFIRLFGSYNILDLLPWLRFFPLQDMKTLRESRQIRDDILGAKYQEHKKRFEEDSANNNFE
IEDLTDALLKAYHEAQEEDGKVAQLLTEDHLVMTMNDVFNAGLETSATTLRWLLAFLVTYPDIQARVHAELDDVIGSGRMPCLNDRGNL
PYLESTIAEVLRIRAIVPLSLPHKATCDTTLGGYDVPKDTMLISNIWAMHHDKNEWQNPEVFDPSRFLDKDGKLLGSERKFSAAGVRSY
LPFSAGRRGCLGESLAKTEMFLVASRLLHQFKFENPAGRPLPDLSGEVGVVLMPGPFEICAKDRL

>Ofa_CYP17[*Orbicella faveolata;* XP_020626306.1]
MILEIALVCSCVMLIIFLIQRARGQKALSLAPGPSPYPLIGNMLRLAGKPGHQAMTELAEEYGKIFTLYLPGGQRCVVINSIDLAREAL
LTKRDDFSGRPSTFISGYLSRGGKDIICADFTQTMILLRKIAHSALRMYGSGLQHLEGLICSEVSQLAKRISAHEGEPLDPKNDLQLTV
LNVICAVVYGESYDIEDEEFQRIIEYNDDFIRLFGSYHILDLLPWLRFLPLEDVKTLRKSRAIRDDILGTKYREHKKRFEEDNANNNFE
MEDLTDALLKAYHEAKEEDGKVEQLLTEDHIVMTMNDVFNAGLETTATTLRWLFAYMAHYPEVQARLHAELDDVIGCGRMPCLKDRGNL
PYLESTIAEVLRIAATVPLSLPHKATCDTTLGGYDVPKDTMLITNVWAMHHDKDEWEKPEVFNPERFLDKEGKFGSSERKVSAAGVRSY
LPFSAGRRGCLGESLAKTEMFLIVSRFLHQFKIENPPDKPLPDLTGRVGVVLMPGPFEVCIKERF

>Spi_CYP17[*Stylophora pistillata;* XP_022798766.1]
MILEIAFVIVCVTTIAFFVHRAHAQKSKNLAPGPRPYPLIGNMPRLAGKPGHLAMTELAEEYGKIYTLYLPGGQRCVVINSIDLAREAL
LTRRDEFSGRPVTFVGDYLSRGGRNIICADFTQSMILLRKIAHSALRMYGSGLKHLECLISSEVDHMAKRISSQQGKPFDPKKEIQLTI
LNVICAVVYGESYDIEDEEFLRIIQYNDDFIRLFGSYNILDLLPWLRFLPLQDMKTLRESRQIRDDILGAKYQEHKKRFEEDSANNNFE
IEDLTDALLKAYHEAQEEDGKVSQLLTEDHLVMTMNDVFNAGLETSATTLRWLLAFMVTYPEIQARVHAELDDVIGSGRMPCLNDRGNL
PYLESTIAEVLRIRAIVPLSLPHKATCDTTLGGYDVPKDTMLISNIWAMHHDKDEWLNPEVFDPSRFLDKDGKLLGSERKFSAAGVRSY
LPFSAGRRGCLGESLAKTEMFLVASRLLHQFKFENPVGKPLPDLSGEVGVVLMPGPFEVCAKDRM

CYP3A

>Hsc_CYP3A[*Holothuria scabra*]
FHKFIKQGFVQALQGMRERHGPIYGAYIGSTPATILADADILKEVLIRNFSHFHNRRGTKIDDYPLNKALTRLEGARWKFVRNTITPAF
SASKMKPISVLTNRCCDNLIVHMKTLIATNPEFEVKGVYGNMTMDTIAQSAFGLEIDSQSNPNSPFVKHAMAIMNMKLTSPRILMAFWF
PQLAPIFNYFRIGMIPNIHKQFFIDVTSKAVSLRKDPKNKRLDILQLMVDAQENPDKSESEEIPDLAGYKEKDEMKKELTTEELMAQAV
VFFLAGFETTNIALSFMSYLLATHPEIQEKLYQEIQDVSSSAESFSYDVVNKMEYLDMVVSEALRLYPPGSVADRVCTEDITINDVKFS
KGDSIFYNIYSLHHDSRYWEDPQKFDPERFSKENKDKIHPFAYLPFGTGPRNCIGMRFAVLVVKTALARVVQKFKFEPCEKTQIPVKLG
KRSIMPEIGITLRVIERE

>Mmu_CYP3A[*Mus musculus;* BAB20498.1]
MNLFSALSLDTLVLLAIILVLLYRYGTRTHGLFKKQGIPGPKPLPFLGTVLNYYTGIWKFDMECYEKYGKTWGLFDGQTPLLVITDPET
IKNVLVKDCLSVFTNRREFGPVGIMSKAISISKDEEWKRYRALLSPTFTSGRLKEMFPVIEQYGDILVKYLRQEAEKGMPVAMKDVLGA
YSMDVITSTSFGVNVDSLNNPEDPFVEEAKKFLRVDFFDPLLFSVVLFPLLTPVYEMLNICMFPNDSIEFFKKFVDRMQESRLDSNQKH
RVDFLQLMMNSHNNSKDKDSHKAFSNMEITVQSIIFISAGYETTSSTLSFTLYCLATHPDIQKKLQAEIDKALPNKATPTCDTVMEMEY
LDMVLNETLRLYPIVTRLERVCKKDVELNGVYIPKGSMVMIPSYALHHDPQHWPDPEEFQPERFSKENKGSIDPYVYLPFGIGPRNCIG
MRFALMNMKLAVTKVLQNFSFQPCQETQIPLKLSRQGILQPEKPIVLKVVPRDAVITGA

>Ssc_CYP3A[*Sus scrofa;* AAL13316.1]
MDLIPGFSTETWVLLATSLVLLYLYGTYSHGLFKKLGIPGPRPLPYFGNILGYRKGVDHFDKKCFQQYGKMWGVYDGRQPLLAVTDPNM
IKSVLVKECYSVFTNRRSFGPLGAMRNALSLAEDEEWKRIRTLLSPTFTSGKLKEMFPIISHYGDLLVSNLRKEAEKGKPVTMKDIFGA
YSMDVITSTAFGVNIDSLNNPQDPFVENSKKLLKFSFFDPFLLSLIFFPFLTPIFEVLNITLFPKSSVNFFTKSVKRMKESRLTDQQKR
RVDLLQLMINSQNSKEMDPHKSLSNEELVGPGIIFIFAGYETTSSALSLLAYELATHPDVQQKLQEEIEATFPNKAPPTYDALAQMEYL
DMVVNETLRLYPIAARLERACKKDVEIHGVFVPKGTVVVVPVFVLHRDPDLWPEPEEFRPERFSKKNKDTINPYTYLPFGTGPRNCIGM
RFALMNMKLALVKVLQNFSFKPCKETQIPLKLTTQGLTPTEKPVVLKILPRDGPLSGA

>Mfu_CYP3A[*Macaca fuscata;* BAE45352.1]
MDLIPDLAVETWLLLAVTLVLLYLYGTHSHGLFKKLGIPGPTPLPLLGNILSYRKGFWTFDMECYKKYGKVWGFYDGRQPVLAITDPNM
IKTVLVKECYSVFTNRRPFGPVGFMKNAISIAEDEEWKRIRSLLSPTFTSGKLKEMVPIIAKYGDVLVRNLRREAETGKPVTLKDVFGA
YSMDVITSTSFGVNIDSLNNPQDPFVENTKKLLRFDFLDPFFLSITIFPFIIPILEVLNISIFPREVTSFLRKSVKRIKESRLKDTQKH
RVDFLQLMIDSQNSKETESHKALSDLELVAQSIIFIFAGYETTSSVLSFIIYELATHPDVQQKLQEEIDTVLPNKAPPTYDTVLQMEYL
DMVVNETLRIFPIAMRLERVCKKDVEINGIFIPKGVVVMIPSYALHHDPKYWPEPEKFLPERFNKKNNDNIDPYIYTPFGSGPRNCIGM
RFALMNMKLAIIRVLQNFSFKPCKETQIPLKLRLGGLLQTEKPIVLKIESRDGTVSGA

>Ola_CYP3A[*Oryzias latipes;* AAG35209.1]
MGNFLYFSAETWTLLVAFITLLLVYAYWPYGTFKRLGIPGPKPVPFFGTMLAYRKGFPIFDEECHKKYGKTWGIYDGRQPVLCITDPAT
IKAVLVKECYSLFTNRRNFHLNGPMYDSVFNAEDDQWKRIRSVLSPSFTSGRLKEMFDIMKHHSANLVNSMKKKADKDEPLNLKDHFGP
YSMDVVTSTAFSVDIDSLNNPSDPFVTNIKKMLKFDFLNPLFLLVAFFPFLGPIMEKMELSFFPASAFDFFYSALQKIKSDHKNNKQKS
RIDFLQLMIDSQKNNQNGLQDKGLSDHEILSQAMIFIFAGYETTSSSLTFLAYNLATNPEVMKKLQKEIDATFPNKAPIQYAPLLEMEY
LDSVVNESLRLYPIAARLERVAKATVEVNGLVIPKDMVVMVPTWPIQRDPELWPEPEKFKPERFSKETKETFDPYTYMPFGAGPRNCIG
MRFALVSMKLALVEILQQYSFSVCKETEIPFELDIQGLIAPKRPIFLKLVPRSAC

>Sko_CYP3A[*Saccoglossus kowalevskii;* XP_002731620.1]
MEMSWFHSFSPTTFVLVVIVIVLLYLYSTWTFSTFKNMGVRGPTPWPFIGSMLEYRKGMVIKDLEWSKKYGKVFGIYEGRSPMLLVTDP
EMCKQICVKHFSSFRNRKAIPLRSKPMSSGLFQLADSRWKVTRNTLIPSFSGSKMRLMSPLISRCADELIKNMEAHCKEGKTVQCKELF
GGYVVDSIASVGFGMDVSSQTQPEHPFVEHAKVAFTFGVFSPAFAIVFFFPFLVPLLNYFEIPLLPKKTVDYFAQVVEEAIKLRKSDEC
ASQRVDFLQLMINAHDVYDEYVKNKEDEEDRDEGVNRVEFIKDGAHSPVNLSKGLSKDEMLAQSIVFFIAGYETTNTTMSFVCYNLATN
PETQVKLQREIDEVMRNYDDVGYEAVSKMKYLDMVVSETLRIFPPPSRFNRECNQDVNINGINIPKGMTVSVSPYVIHHDPDNYPDPEK
FIPERFTKEEKEKRHPYAWIPFGAGPRNCIGMRFALIEAKIGLVRVLQKFTFEPCAETEIPPVFGKQLILSPPNGIKLSVKLRA

>Aja_CYP3A[*Apostichopus japonicus;* PIK42887.1]
MELTTWVLIASVIIIACVYDYWRKSYFKQRGIPCDTILPFLGHYHKFISGGFTEFIHRSAKQHGKIFGMLLGSSPVVVIGDADILKEIL
VRKFSSFHNRRPQAIDDYPVNKALTRLEGARWKQVRNTITPAFSASKMKPIAALLNDSCDALIKHMKAKVATNPDIEVKEMYGNMTMDC
IAKGGFGLQVDSQEDPNDPFVNHAKAIINMNLLNPRVIIASKKFFMDVTQQAMHMRHDKASKRVDVLQLMVDAQETDDSPAESEDMPEI
AGGEEKGLHRKTELTEAEIMAQSLIFFLAGYSTTTVALSFVSYNLATHPELQDKLFQEVQEMAPDRDSVNYDTVNKMEYLDKFLSETLR
LFPPLLYSIRISETGAHHVGADRVCQDSVTVGDFHFEKGMDIFYNIIGIHYDPDYWEDPEKFDPERFTKEATEKRHPFAYLPFGTGPRN
CIGMRFALLVLKMAIVRMVQTFQFEPCEQTQIPIKFGKRSVSPDKGITLRVVARE

>Apl_CYP3A[*Acanthaster planci;* XP_022082383.1]
MELLGFELSLTWFLIICVVLVAAWYDRWCHQYFQRRGIPVPDYIPFFGNILQWRHGVQKTFTDYVKKHGRVVGMYDFRRPVLIVNDPDI
LKNVLVKNFSSFYNRRKFPLGQEPLNRGLTQLVNEEWKETRNVITPSFSASKMRQMSVMINECCDTLVTNFSKAQEGGKSADCRVLFGG
FTMDAIAKCGFGLKVDSQQNKDDPFVQNAKKAFEFSLFNPAFMIVSAFPALAPVLAYLKFDLFDPKTVKFFMDVTEAACNIRRQEGSEA
SKHIDFLQLMLNAHNDEEHDEEDTPTIENGTDKGVVHRRALSKIDVMAQAITFFLAGYETTNTLLSFTAYLLATNQDVQEKLHAEIDDL
APTRDNLGYDVIAKMEYLDMVINESLRMYPPAVIFDRMCNETITYDGLTIEKGVNVFVSLWTLHYDEEFWPNPTKFDPERFSPENKASI
KPCTYMPFGFGPRNCIGMRFALLESKMALVRVLQQYRFDVSSETEIPPTLGKTGFLAPKSLMLKAVQRN

>Spu_CYP3A[*Strongylocentrotus purpuratus;* XP_011666125.1]
MNRELWNLTYFWRRGIKCSRPLPLLGNILSCTKGFYSYIYTCQQNYGNIFGLYFFRSPLIVVCDPDVLRDVLVKSFSKFSNHYQFFLKN
RPLDQGLLDLRDQRWKDVRSVISPTFSALKMKQMSPVINECCDVLVTNLKKKQGEDINVNSVFEAFTMDGIAKCAFGLQVDSQNNPDDP

FVKHAKRIMDGTVESPVAMIAGVFPPAAYLFNALDIALFPAETRKFFRDVVVKAIELKKRSAGNERRDFLQLMMNAHNDDEHDSRSKVI
KDDNDLHNLIEDHQDKHVSNAQNTKTQLTMDEIFAQAVLFFVAGYETTNVTLGFLAYALATNPDIQDRLIEEVDEVTPTRDSVDYNSIA
KMSLLDMVVCETLRLYPPAVMGDRCCSETHTVNGLTIEKGVQFLYSIYNIQRDPTLWPEPEKFDPSRFTKENRANRHPFAWIPFGAGPR
NCIGMRFALMEIKMAVVRILQKYRFVPSPKTDIPVKFGTGNTLKPDGGIFLRVEERQ

>Cvi_CYP3A[*Crassostrea virginica;* XP_022315198.1]
MDVLGFLDIKGWILLTAFSLLSVYLYFKWKFGLWKRLGVPSPNYFPYIGFIKAIRDKGFAELDVELVQKHGKLFGLYFGNTPSLVISDP
DVMKDIMIKDFSKAPNRYALINRRDELKHSLTEVVDDHWRFMRNTILPTFSSGKLRKMTPLLKVKYQMLMDGLKKRADQGEVIEFKEVF
GNYTMDVIASLGFGMEIDSQTNPKNSFVKYAKELFAVDFNPLIVLAAFSSKFDAVLDYFKISPLNNRRIMGFYQSAVQQAMEMRDDTDK
NRKDMLQLMLNAHTDTDSNEIEDKHAYKNPEEWKKRGLTVDEITGNAILFMLAGYDTTSSTMTFMAYNLATNPECQDRLINEIDSVLGE
EMPTYDNMQKLDYLDMVFSETLRLYPPATRTNRCNSEEIVIDGVKIPKNIELVFPIFAIHRDPNYWPEPEKFDPERFTAENKANRHPYV
YLPFGHGPRNCIGQRLAAMEAKCAIVYILQHYRFVTCEETEIPLKLNKEALVSPANGVKLRLEKR

>Cgi_CYP3A[*Crassostrea gigas;* XP_011456748.1]
MDVQCLLDIKGWILLAIAVLLTMYMYIKWKFGIWHRLRIPGPDFMPYIGQMKQFREKGLAQLDVELVQKYGRLVGLFFGSTPSLMISDP
ELIKNIMVKDFSKAPNRFALITRRDELKYSLTDVVDDHWRFIRNTILPTFSSGKMRKMSSILQVKYKMLLDALNKRAAEGEVIEFKEVY
GNYTMDVIASLGFGMEIDSQTNPDNTFVKYAKELFTVKFTPLLILAAMSSKIDAVLNYFKISPLNNRKVMEFYKSAVHQAVGMRDDTDK
DRKDMLQLMLNAHKDTDRNEIEETHTYENPEQWKKRGLTVEEITGNSILFLLAGFDTTASTMTFMTYNLATNPECQDRLIEEIDSVLGT
ELPTYDNIQKLEYLDMVFCETLRLYPPATRINRSNLDEMDINGIKIPKETELTFPVFAIHRDPEFWEEPEKFDPERFTPENKAKRNPYV
YLPFGHGPRNCIGQRLAAMEAKCALVYILQHYRFVTCSETEIPLELSKDALMKPANGVKLKLEKKITD

>Med_CYP3A[*Mytilus edulis;* BAH24057.1]
MDVLGLVDIPSWIFTILLFIFTLYLYTLWKHTLWRRLGVPGPIPFPILGSIKDLAVKGVSKTDQDLVPKYGSVVGVYNGTIPVLLVTDT
DLIKEIFIKEYQTFTNRVLILKTDDLLDSMITAAEDDHWKFIRSTMTPTFTSGKLKNMLPMILRCCDDLVNNINGKLEQNDTVEMTEVF
GGFTLDVIASTAFGLQVNSQKEPDNEFVTNTKKTLTDSVVKPYFVIVMIFPFLKNLMGTLFKAPVFGEGSEDFFRNIVQQLVAERKSNS
NSHFRDFIQLMLNSHKDKTKEKENGRIDELGGLDFSEYKNRGMNENEILTNSMVFFGAGFDTTKTTPSFAAYVLALHPDMQERLFTEID
NELGKSPPTYDSLPNMKYLEMFLAEVLRLYAAVPRINRRAKSDITVNGMFIPKDTDVTIPISALHRNPKYWPEPEKFDPERFTDENKAT
RPMYSYLPFGLGPRVCIGMRLALIETKYALISTVQNFKFVVGSKTEIPMPLEKGFITRPLNGIHLKIEKR

>Mye_CYP3A[*Mizuhopecten yessoensis;* XP_021350792.1]
MEVLGLVDIPTWLQWVMLLVGLVVTYVIYMSWDHNTFKKMGIDGPKPDPVFGNMRMLTEDGVIKAELDMYRKYGKVFGMYESYVPVLYI
ADPALLKDILVKDFKNFVNRRDTFRKFSDSKMNLMLTQLQDDHWRFVRNTITPTFSGKKLRQMTSLINNSADKFMEHIDQKIKEDEDID
MKGFIGGFTMDVIASTGFGLDVNSQNNPDNSFTKHARYFSDPTSIAVLVVIVFFLPFFWWILRLSAFLGFNLTKVRTAMNFFARVTEKA
LTERRQSPGEHFDFLGIMVKAEKGDEPTEEEKGDELLKENDASETLSTWTRKTLTVEEITAQATLFFVAGYDTTSNSLCFLMYHLVLYP

EICDNLLQEIDDKLNGQAPNYDNVAKLSYMEMCINETMRLFPAASRLDREAKNDVTIGNIRIPKGMIINIPVGAIHMDPEYWPEPEKFD
PERFTPEAKANRDPFVFMPFGAGPRNCVGMRLALLAMKIAIVRILQNYRPVKSPKTEIPINVSKMGNIPLGLYLKFEKRT

3beta-HSD

>Hsc_3beta-HSD[*Holothuria scabra*]
MSSDWIGSRVLILGGTGFIGRHMVKYLLQNELTTKIRVVDKTPIEMAWMNEEHK

>Hsa_3beta-HSD[*Homo sapiens;* NP_000189.1]
MGWSCLVTGAGGLLGQRIVRLLVEEKELKEIRALDKAFRPELREEFSKLQNRTKLTVLEGDILDEPFLKRACQDVSVVIHTACIIDVFG
VTHRESIMNVNVKGTQLLLEACVQASVPVFIYTSSIEVAGPNSYKEIIQNGHEEEPLENTWPTPYPYSKKLAEKAVLAANGWNLKNGDT
LYTCALRPTYIYGEGGPFLSASINEALNNNGILSSVGKFSTVNPVYVGNVAWAHILALRALRDPKKAPSVRGQFYYISDDTPHQSYDNL
NYILSKEFGLRLDSRWSLPLTLMYWIGFLLEVVSFLLSPIYSYQPPFNRHTVTLSNSVFTFSYKKAQRDLAYKPLYSWEEAKQKTVEWV
GSLVDRHKETLKSKTQ

>Mmu_3beta-HSD[*Mus musculus;* NP_038849.2]
MPGWSCLVTGAGGFLGQRIVQLLMQEKDLEEIRVLDKFFRPETREQFFNLDTNIKVTVLEGDILDTQYLRKACQGISVVIHTAAVIDVT
GVIPRQTILDVNLKGTQNLLEACIQASVPAFIFSSSVDVAGPNSYKEIILNGNEEEHHESIWSDPYPYSKKMAEKAVLAANGSMLKIGG
TLHTCALRPMYIYGERSPFISNTIITALKNKNILGCTGKFSTANPVYVGNVAWAHILAARGLRDPKKSPNIQGEFYYISDDTPHQSYDD
LNYTLSKEWGFCPDSSWSLPVPLLYWLAFMLETVSFLLSPIYRFIPPFNRHLVTLTGSTFTFSYKKAQRDLGYEPLVSWEEAKQKTSEW
IGTLVEQHRETLDTKSQ

>Oar_3beta-HSD[*Ovis aries;* NP_001129404.1]
MAGWSCLVTGGGGFLGRRIICLLVEEKDLQEIRVLDKVFRPEVREEFSKLQSKIKLTLLEGDILDEQCLKRAFQGISVVIHTASVIDVR
NAVPRETIMNVNVKGTQLLLEACVQASVPVFIHTSTIEVAGPNSYREIIQDGHEEEHHESAWFCPYPYSKKLAEKAVLEANGWALKNGG
TLYTCALRPMYIYGEGSPFLSAYMHGALKNNGILTNYCKFSRVNPVYVGNVAWAHILALRALRDPKKVPNIQGQFYYISDDTPHQSYDD
LNYTLSKEWGFCLDSRMSLPISLQYWLAFLLEIVSFLLSPIYKYHPCFNRHLVTLCNSVFTFSYKKAQQDLGYEPLYTWEEAKQKTKEW
IGSLVKQHKETLKTKIH

>Rno_3beta-HSD[*Rattus norvegicus;* NP_058961.4]
MPGWSCLVTGAGGFLGQRIVQLLVQEKDLKEVRVLDKVFRPETREEFFNLGTSIKVTVLEGDILDTQCLRRACQGISVVIHTAALIDVT
GVNPRQTILDVNLKGTQNLLEACVQASVPAFIYCSTVDVAGPNSYKKIILNGHEEEHHESTWSNPYPYSKKMAEKAVLAANGSILKNGG
TLHTCALRPMYIYGERSPFLSVMILAALKSKGILNVTGKFSIANPVYVGNVAWAHILAARGLRDPKKSQNVQGQFYYISDDTPHQSYDD

LNYTLSKEWGLHLDSSWSLPLPLLYWLAFLLEIVSFFLHPVYNYRPSFNRHLVTLSNSKFTFSYKKAQRDLGYKPLVSWEEAKQKTSEW
IGTLVEQHRETLDTKSQ

>Dre_3beta-HSD[*Danio rerio;* NP_956103.1]
MSNNNKSKLTYVITGGCGFLGQHLLRVLLEKKKNVKEIRLFDKNVFPSLQSESTEDVKVVIIQGDITKYEDVRNAFLGADLVFHAASLV
DVWYKIPEKVIFAVNVQGTENAIKACVEIGIQYLVYTSSMEVVGPNVKGDEFVRGNEDTPYNIFHEMPYPKSKAAAEKIVLEANGTKVE
GGNILYTCCLRPTGIYGEQHQLMKDFYLNSVRNGGWVMRGVPPHTEHGRVYAGNVAWMHLLAARALQEHPNRLGGECYFCYDDSPYKPY
DEFNMQFLSAFNFRSLRLPVWMLWIIAWMNDMVRWVLKPIYNYTPLLNKYTLAVACTSFTVSTDKAFRHFQYQPLYSWQQCLSRTQSWV
NTFPFETSTKDK

>Ola_3beta-HSD[*Oryzias latipes;* NP_001131037.1]
MSLRGDVCVVTGACGFLGKRLVRLLLEEEETAEIRLLDKHVPQQVLQSLEDCRGGTKLSAFEGDIRDSDFVRKACRGATNVFHTASMID
VLESVEYSEIYGVNVKGTQLLLEACLHENVMSFIYTSTIEVVGPNPRGDPMVNGTEDTVYDSRLTLSYSKTKKEAEDRTLQANGQLLQN
GGRLATCSLRPAYIFGEGCRFLLGHMTDGIRKGNVLNRLSAREALVNPVYVGNVAFAHLQAARSLKEPQKRDTVGGKFYFITDDTPHLS
YADFNYCMMSPLGFSVQDKLQMPLRIFYIVIFFLEALCMLLRPFIRIVPPMNRQLLTLLNTTFTFSYQKAKRDLGYFPKYSWEEARRRT
FDWLASQLPQQRETLQSN

>Sko_3beta-HSD[*Saccoglossus kowalevskii;* XP_002738669.1]
MSSEEEGDVILVTGASGFVGQHVVKLLMERCRDVREVRAFDIRPFKWIKELRVTETYVDLVHVRGDITQLSDIRKACRGVDAVVHTAGY
VDVGSLPDMEKLKAINIVGAENVLKACIDNHVTRLVYTSTQDVVLGMEPIENADESSVGIPNTFLYEGYAGTKYEAEKIILKANSLILE
NGRKLKTCSLRPTTMYGEGDIYFLPPTLKASKQQGGVLMRIGDGKALFHASYVGNVAWAHILALQQLKRQRSEDDISGQACFISDDTEP
MNLFDFMEPFLQARGFRLSRYHIPYWFMYIVAFIVEFLAWFLQPFSKINFPINRNVLHHMCTSCYFSYHGAKRYLNYSPLFSVEESMER
TVRYVKRLRL

>Spu_3beta-HSD[*Strongylocentrotus purpuratus;* XP_011664172.1]
MPGTEGEVVMVTGASGFLGQHILKQLLEQGEFLIKEVRTFDLQPFTWCPELEVHNPTTQLSHIKGDLLCMEEVRRAFKGVTVVIHTAGV
IDVSPVPDAELLRSVNIQGSENVLQACIHHNIQYLVYTSTVDVVIGQEPITAGTETILGIPQHHHFGLYATTKYEAEKIVLKASNLILK
NGKRLQTCALRPTPVYGEGDTYNRDVLRQACHYKMMVRMGSESSRYQCTYAGNIAWGHVLAVKELLKPTTNESPAGQAFFLTDETPVSK
VSDFFTPFVIGVDAKMSSFSLPFWLLYSIAVVIEICAWLLQPIYKVKFFLTTATVTYAYGVYYFQCEGAERCLGYEPLYTYDDAVERSL
VYYRRECGLS

>Aja_3beta-HSD[*Apostichopus japonicus;* PIK61940.1]
MTNQEGVTILVTGGAGFLGQHIVKELMEQEVFPVKEIRSFDIQPFKWHHGLHPSKNSQKLYHIEGSICDPAALTQACENVDVVIHNCSC
VAFGAYPNRKSTRAVNVDGTENVVDACIEQNVECLVYTSSIEVANDLKKPSVNASETSVTVPAINPIFHVYSKSKYDAEKMVLTANNKT
LKNGKKLRTCALRPCGIYGEGDHILTTNLGYFLNAGIAFRIGEPSGKSQYAYAGNLAYSYVFAVKSLLRPDEKTDISGEAFFLGDDTPI

YSIGELVSHFAKALVAETGTATPPYWLMYTIAFFLTVMCWLVSNLKYWEMPFHTNAVRYMFGEYHYSWEKLQRVLGYKPRYTYKEAFDR
TITYYEHFFGLNNKSNQTKKQK

>Isc_3beta-HSD[*Ixodes scapularis;* XP_002407062.1]
MGDGDSSRKYRFLVLGGTGFIGRHLVDFLLKHNLATKIRVVDKVPPQMGWLNANHKVVFEDPIVEYKSANLINQASCKAAFEGSEEPFD
FAVNLAGETKINLPDSVYQEGIAKLSENCAKEAIAHNIKRYIEISSGHIYAEGKKPSQEESALDPWTAVARFKRDVEKTLEAFKDLDYV
ILRPALVYGVADKRSITPRILVGAVYKHLKETMKLLWTKDLKMNTVHVSDLCSAVWHVCLHGKAREVYNVVDPGDTTQGKITDIICEIF
GVNHGYWGTAASFLAKADMQGAVQEINEKHLAPWAEACQKDAIANTPLSPYLSQELLYDKNLCMDGSKLQSIGFTYRVPEINKDCINEI
IDDYIEMNIFPKSLIK

>Aae_3beta-HSD[*Aedes aegypti;* XP_001650664.1]
MDKKADMEAKEVILITGGAGFLGQHLIRTLQERDPKVREIRVVDLKPYENILGHIDTHRVVSYVGDICQPSKIESAFEGVDCVFHMAAY
INFDFPPNHQELQRVNVDGTQMVIDLCRKYNVPRLVFTSDCLIHMTPYMGRANFTIVCNQTEPKTKVPAKESEFQIPGYASSKFRAENL
VLAANDTPTANGGMLKTIAIRPPVVFGEGDQRFIPSIIKVALRFNGEIPKLAGPGGKQQLVYAGNVAWAHLRAKDALLTKPKEISGHPV
FVTDDSGIEDTTRFCQRLSRANETLKLRPSWYQIPLFLSFFLAFLLELLIKALNPIVKIKLSFPPCGLLSYMSSILLYNRIRASIYLDY
EPIYGEEKAVSNAALWYEKWYQDYCQQHKLKKLKVK

>Dma_3beta-HSD[*Daphnia magna;* KZS12706.1]
MGSEVVLVTGGQGFLGQHIIRLLQQDPKVAEIRVIDKKMFTNRIGFEEKKVVKSFVADISKPETMGNEIFDGVNCVIHTAAETGFSHFP
SAKIMEEVNVNGTRHVIHKCITANVGCLIFTSTTHVVIPKKDPLFLATEHLAKAPPKDGFLFGHYGRTKHDAENLIRDANGKRLADGSG
NLATLCLRPTLLYGELDPYYVTHALKIAKDHGGCLYRIGWGGERIQVTYAGNAAWAHILAKDQLMKEPTIVGGEVIFVTDDTVIQNAFE
FLDPFLKARSMKASTFVYPATLAVIFVLFLYFVSRMLRPLVHLKNPFPHPSTLYFIVCFYCFNRLKATLRLGYKPIYSPEEAISKSLDY
YKNVPLS

>Tme_3beta-HSD[*Tropilaelaps mercedesae;* OQR69050.1]
MYRSSGKQFGLRSRLRYILSSGPEGVISGHQRQRQTSKMVPLPSIENTEDIDSLWRGPSVGARTTDTVLVTGSSGCLGQHIVKLLQEHD
STCKNIRLFDVKPYKNLLNHAVQKPMKEIQGDLTNAKEVLDACEGVDCVIHCAALVDISLFPNEAALEAINVEGTRNVIDACIRQNVPY
LVFTSTTDTVVSSNHIFYGAENTTFVPKNFLMGPYAETKHRAEQLVLQANNRLLADGSTKLRSCVLRPTVFYGEQDEHFIPRIMKIAKY
YGGNKVQRVKSIDERFQVTYVGNAAHAHIVAKNRLRENSDCAGEVYYITDDTPLQELYSAIKPYVDSQGKFRISDWSIPYLLAILGISI
LVLIVRLVRPIYQVGKYFPTPAAVTYACTSVFFNRQKATLRLKYYPHYTVEESHENSLKYYKDLKV

>Nle_3beta-HSD[*Neodiprion lecontei;* XP_015514980.1]
METANFETKPGVIVLGGCGFIGRNLVEYLLDNELVSYVRVIDKVPPQTAWLNKKHQRIFEHPLLEFRSANLINSASCDNAFAGEQRIDI
VFNCAGETKYGQTDPVYKDGIHKLSMNCAQKAAELGVQRYIEISSGHLSSSEKVPHKEDDEVDPWTFIAKHKVQVENDLKNIPGLKFTI
FRPAIIYGPGDRNSLAPRLVVGAVYKHLGEMMKLLWGPNLRMNTVHVRDVVRAMWHVTRHPNTIGQIYNVVDESDSTQGSISALVSELF

NINHDYWGAALSMLAKVDMTTIVEEVNDKHMSPWAEACSKDGVENSPLSPYINRELLYNKNLYLQPGKLSSTTNFTYLYPKLMKESLKE
VVDDYVEMKIFPHSLVL

>Can_3beta-HSD[*Crassostrea angulata;* AFK26304.1]
MCTVTVTGGAGFLGQHVIQHLQLSAPWVTEIRVFDLIPFSRSLDYTPRLDVITYTGNLGDTESLRKAFHNASAVLHLASAIDARFQPDK
KLLQEVNVKGTANVIAACVEEGVPILIYCSSIGTVQGYFNCQGGSETELKDVRPLLFRDYGGTERVAEQQVLKADQTPLANGKKLRTMS
LLPPTMYGEGDKLIAMMLQYAEDNNGSFIRMGNGENLEAYAYAGNVAWGFVCCLKTMYNNPSFGNERMFIMDDTPPQSIQALSRPYLES
RGFQMTSYYVPLSIVFFICFLVETVCLLISPFKRFSFPLSLSGIIFSARKFYVRYDKAKTLIGYVPPFSVEGARERSLPYYNNVKLRKN

>Ovu_3beta-HSD[*Octopus vulgaris;* XP_029645198.1]
MFLLQQHRRHHLNVKALRYSSNMAPERRAKQVCVVTGGSGFLGQHIVHLLQTKCDDVREVRTFDIRPIKKFLEYEDNFSSRHIIGDVTN
YTDIMQALDGVDVVYHVAGVVSYGTRPDTELMQKVNVSGTENVIHACIEMSVPRLIFCSTVDVVVGDKNIVDGTEDTAITPSHFLFPGY
PQTKHEAEDLVCRANGSHLANGGRLYTVSLRPNVMYGEGDPFLIPSALQSAYSAGGWSVCVGNGKAIFQVSYAGNVAWAFLMANRTLLD
NKLTNVEGQYFFVPDDTPATSIYTTLEPYLHDRGFKHMPVYIPYWLIHGLLSIRDLFLWLISPLVKINAQPPICSIKYINMTLTFKNDK
ARTYLGFRPLFNPEEALQRSRKYYKKIKLN

>Mye_3beta-HSD[*Mizuhopecten yessoensis;* XP_021363944.1]
MRNGLTFWTRRGKYHSHLSRSLSKGRKLPANNFAMTTTKDFNQSAAGEVVMVTGGAGFLGQHIIGHLQEKASHVSEIRVLDIRPYSNKL
GHRESKPLTSTLGDICDQQTVQQACQGVTTVIHVAGLVSYGTFPDINGMQRINVQGTRNIVESCLNTGVERLIFCSTVDVVVGFDDIRE
GMETTTCVPKQFLFPGYPATKYEAECLVLNANGQKSVNGNPLWTMSLRANVMYGEEDPFYVTSGLRSAASNRGRLVQVGNGQSKFQQVY
VGNTAWAFVCADRALRLNYNLGGEIFYIPDYTPVQNSFLFMQPYLESRNYGLSQYRLPCGLVYYSLYLMELVLHLLAPLIRINLPTASC
SVRYINMDLYFNGSKGRRLLGYQPLYSPQEAEKRSLEYYKHVKL

>Cgi_3beta-HSD[*Crassostrea gigas;* XP_011438773.1]
MPCTVLVTGASGFLGQHVVKTLHEKAADFVTYLVLFDKDTFVQKLDYKPKIPIECITGSITDPEVVRKAVQKVDCVIHLAAVISVSTFP
NVKAMQDVNVKGTALLLDACVGANVGIVITCSTQDTAVDLTDQTMVDETYKVEPRNLAFGDYARTKLEAEKIVLAKHGSRLKNGDRLRT
VVLRPGVLYGELDAIFVTEVIRNAKKQGGVLYHFGSPQAVSQYAYVGNVAWGFVCALRKLSQDTSIGGEAYFIGDETPLMQLFKFSELF
IKLYGGRLSSRPIPYTLILVIVLLLEWIVWLISPIKQINLPISSSTVRYANKKWSFSYNKAKTELNYSPLFDWEESYSRSCEYYKTAVT

17beta-HSD

>Hsc_17beta-HSD[*Holothuria scabra*]
LSREGKVDILINNAGYGQSGSFEHVSLEKCRALFETNFFGAVHLTQQFIPSMKKRKSGHILFISSTVSTFGIPFCEIYTASKCAVDGVA
EALAPSLALFNIKVTLICPGPVETNFAPNIIMFGQDENDEETMDIKNQYLKNLQQIIGENLQKRDEVAAVIKEAIEAEKPNLRYYTSEK
DLQDAKNKLVDTTGNCAIEFLNNSYFKGVKLNSQ

>Mna_17beta-HSD[*Miniopterus natalensis;* XP_016057672.1]
MDRTVVLITGCSSGIGLHLALRLASDPSQSFKVYATLRDLSTQDQLWEAARSQGCPPGSLETLQLDVQDADSVAAARARVTEGRVDVLV
CNAGRGLHGPLEAHSADAVGSVLDVNLVGMVQMLQAFLPDMKRRRSGRILVTGSMGGLMGLPFNAVYCASKFAVEGLCESLAVLLPPFG
IHVSLIECGPVRTAFPEKVQDGLDVVLEGADAQTRDLFSQYQRHFEQVFREGAQDPEEVTEVFLRALRSSQPALRYFTSERILPLVRLR
LADPSGCSYVAAMHRAVFSDKPAAGSDGAQTEAGGGELGGPELSASPAALK

>Npa_17beta-HSD[*Nanorana parkeri;* XP_018426360.1]
MEKRVVLITGCSSGIGLGLAVLLASDSHQRFKVYATMRDLSKKEQLLECVRDCHVDTFEILQMDVTDQQSMIGTIEKIKEQRVDILVCN
AGVGLMGPLECHTYDTMKKIFDVNLFGTIGTIQAFLPGMKQRRSGHIIISGSVGGLQGIPFNDVYCASKFAVEGFCESLAIVLQHFNVH
VSLIECGPVNTNFMNKLQNGEPSNSQLQNVDSDTRSLYAQYLQHCQAIFQDVAQDTDEILQVFLEAIEAQVPSLRYFTTQFFMPLTKLK
LSCASGSEYIHAMHKFVFSEAKPKE

>Xla_17beta-HSD[*Xenopus laevis;* XP_018107456.1]
MEKRVVLITGCSSGIGLGLAVLLASDTCQRFKVYATMRDLSKKENLMECVKDCHADTFEILQMDVTDQQSVVSTTKKIKEQRVDILVCN
AGVGLMGPLECHSYESMKKLFDVNLFGTISTIQAFLPGMKQRKSGRVIISSSVGGLQGIPFNDIYCASKFAVEGLCESLAIALQHFNIH
VSLIECGPVSTNFMNNLHSVDTSDSCLQEVDSDTRLLYDQYLQHCQSIFQDVAQDTEEILQVFLEAIEAPTPSLRYFTTQFFMPLIKLK
LSCAGGSEYVRAMHKFVFSGTKQKEEEK

>Cau_17beta-HSD[*Carassius auratus;* XP_026063884.1]
MEQKVVLITGCSSGIGLSLAVHLASNLSKAYKVYATMRNLDKKQRLMESVRGLHKDTMAILQMDVTDQQSILDAKRNITEGRIDILVCN
AGVGLMGPLEAQSLDAIHAVLDVNLMGTLRTIQTFLPDMKRKRHGRILVTGSMGGLQGLPFNEVYCASKFAVEGACESLAILLQHFNIH
VSLIECGPVNTDFLMNLKRTEPSEEALEVDPHTRSLYDQFLQHCQTVFQNAAQDTDDIIQVYMEAIEAQTPFLRYYTNRALLPMSSLKL
TSMDGSQYIRAMSKLIFSAPDTDPQI

>Dre_17beta-HSD[*Danio rerio;* AAP74564.1]
MEQKVVLITGCSSGIGLSLAVHLASNPAKAYKVYATMRNLDKKQRLLESVRGLHKDTLDILQMDVTDQQSILDAQRNVSEGRIDILVCN
AGVGLMGPLETHSLDTIRAIMDVNLLGTIRTIQTFLPDMKKKRHGRILVTGSMGGLQGLPFNEVYCASKFAIEGACESLAILLQHFNIH

ISLIECGPVNTDFLMNLKRTETGDKELEVEVDAHTRSLYDQYLQHCQSVFQNAAQDTEDIIQVYLEAMEAQTPFLRYYTNRALLPMSSL
KLTSMDGSQYIRAMSKLIFSSPGTDAQK

>Bbe_17beta-HSD[*Branchiostoma belcheri;* XP_019619357.1]
MAAAPGRLAGRLALVTGGGSGIGRAVCQAFAAQGSKVAVVDINEAPANETLQSLSCDQGSEHHAFGCDVSSTQSVSRLLTDVTQKFSCA
PCIAVNCAGITKDEFLLKMDNRMFDQVIKVNLKGTFLITQAVGRAMVEGKVPNGSIINMASIVGKVGNLGQCNYAASKAGVEGLTRTSA
KELAKFGIRCNAILPGFIETPMTDAVPQKVLDKFRDFIPIGRLGKPSDIADVCVFLASDESSYITGASIEVAGGLHM

>Bfl_17beta-HSD[*Branchiostoma floridae;* XP_002609475.1]
MAAGTGRLVGRLALVTGGGSGIGRAVCQALAAQGSKVAVVDINEAPANETLQSLSCDQGLQHHSFGCDVSSTQSVSRLLTDVTQKFSCA
PCITVNCAGITKDEFLLKMDNRMFDQVIKVNLKGTFLITQAVGRAMVEGKIQNGSIINMASIVGKVGNLGQCNYAASKAGVEGLTRTSA
KELAKFGIRCNAVLPGFIETPMTDAVPQKVLDKFRDVIPMGRLGKPSDIADVCVFLASEESSYITGASIEVTGTLS

>Aja_17beta-HSD[*Apostichopus japonicus;* PIK53404.1]
MGMKVVVISGCSSGIGLATAVMMAKDSGQRYKVFATMRNLSKKTDLEISAGDQLGKTLFIKELDVSKEESVKKFAEDILESEGRVDILI
NNAGYGQAATLENASLEKSRQLFETNFFGAMHLTQKFIPSMKRNKSGHIIFISSTVTTFGIPFCEVYTASKCALNGLAETLAPQLALFN
VKVSIVCPGPVGTKFAPNMAIIDDAESDTETKAVREKYMKTLTEFISGNLQTGEEVAAVVQEAIQAEKPDFRYYTSPKDLQSASTKYSD
TTGNGVIQFVSKLYFKDIKVGHSSD

>Apl_17beta-HSD[*Acanthaster planci;* XP_022080934.1]
MAPQTVLITGCSTGIGLATAVMMAKDARYKVYATMRNLKSKTDLEKAAGDTLNKSLFIRELDVTKEATIKLVAGEILKENGRIDVLVNN
AGYGEVDAIDQLSLEACQAMMDTNFWGTVRTTRAVLPAMKRQKSGRIINVSSIVAVWALPFNTIYAATKFAVEGFTEAIAVTLKRAYNI
RMSLVEPGPVQTPMVTKFGSVSPAERTKDYDPPLRDEYMNFIEKLAGPMMNTAVSADEVANLILEAIVSENPHLRYQIHEVYKKAAAKK
FIDPHTDAQLDMKNFMDMIN

>Spu_17beta-HSD[*Strongylocentrotus purpuratus;* XP_780174.1]
MAPRVAIITGCSSGIGLSTAVMMAKDPSKGYIVYATMRNLAKKGDLEKAAGATLNKTLFITQLDVTRGDSIKRAVKDVFTKHKRIDILV
NNAGHGSSGYIELVTDQQMRALFDVNFFGVVHLIQEVLPIMKKQRSGRIISVSSVGGITAWPFGEIYAASKFALEGFTESLSIGYRPFN
IWVSSCQPGPVKTAFIANMGSANNQPEFLKLTEEKNIDKESKEVCGKIFKLLINSPTHDEEQQTPEEIASVIKGALEDEKPHLRYATSK
LRLDYAKKYFVDPTGDSFCDTIVPHIIPDSLKQKK

>Mye_17beta-HSD[*Mizuhopecten yessoensis;* AXY92159.1]
MASAVGLLAGRLALVTGAGSGIGKAVCQVFAKEGATVAVVDLHADTANSTLETLPKGDHQAFGADVSCSASVNDLIGQLKDKFSAVPTV
TVNSAGITRDKLMLKMSEEDFDEVIRVNLKGTYLVNQAVGRALVESKAKSGSIINISSISGKVGNVGQTNYAASKAGVVGLTKTVAKEM
GRFGIRVNAILPGFIETPMTDKVPDHLIQMTKMLIPLSRLGKPEEIANACLFLASDHSSYVTGTTLEVAGGLFP

>Cgi_17beta-HSD[*Crassostrea gigas;* XP_011430925.1]
MLSGRLAVVTGAGSGIGRAVCRVFASEGATVIGADMNEKGMEETLAMIKDTGDHQSFQCDVSSSASVNNLLDKIKEKYSSAPHVAVNSA
GITRDRTMMKLTEEDFDKVVNVNLKGTWLLNKAVGKAMLTNKVQGSIVNISSLVGKTGNIGQTNYAASKAGVIGITKSMAKEMGKFNIR
VNAVLPGFIETPMTETVPENLMQMTKLLIPLGRLGNPEEIANTCAFLASDKSSYITGATIEVTGGLFM

>Pva_17beta-HSD[*Penaeus vannamei;* ROT63560.1]
MASGNNFDGKIALVTGGGSGIGRAVCQILARDGARVSVCDINLQGAQETLASLNQPGVHVALHMDVADKESVREALSATQEKLKGTPTL
LVNSAGISRRSSFLEMSEEAFMKVVDVNLKGTFLVSQLVCNAMIERGEAKGGSVVNIASLAGRNGLAMNCQYAATKGGVMAFTKSCAKE
LAKTGIRMNCILPGAIRTPMMAVIPEELIKEALRVNALGRQGEPEGKIVAEMVAFLLSEKSTYMVGACVEVTGGHYA

>Obi_17beta-HSD[*Ooceraea biroi;* EZA54209.1]
MAFICWEKALLVIFAVVTLRILIKLGILVWKKLIAPNLGFGIDLRTQGRWAVVTGATDGLGKAFARALAQKGLDIVLVSRSMSRLKDVA
AEIEQEYRVETRVVQADLTEGQVVYAEIAKTTQDLEVGVLINNAGASYDHPELFTNVSEEILARILQLNVAGVTGVARAVLPGMMERKK
GVLINVSSLAAAIPSPYLSVYSASKAYIIKLSADLATEAAPRGVTVQCVLPGPVATKMSKIKRATWMAPTPEKFVEATLKTVGIESHTT
GYPPHSLIIGVVNTLRYVCETGALWLVTKTMLNIRGRALRKKMKSQAGGRNVSQEDAAVMS

Agarose gel electrophoresis

HscStAR

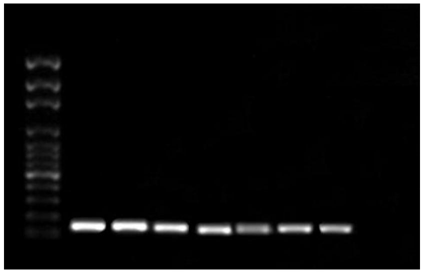

HscCYP10

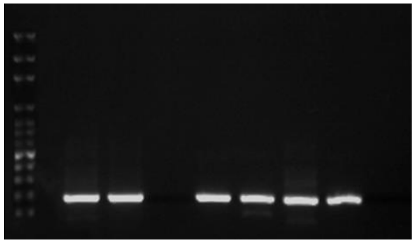

HscCYP17

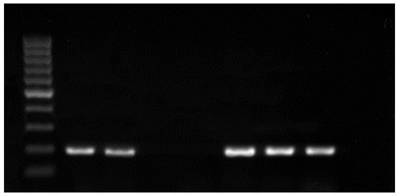

HscCYP3A

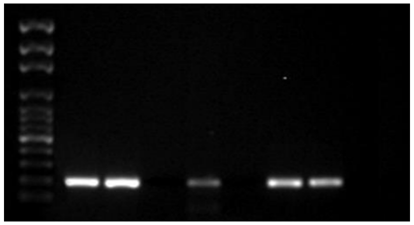

Hsc3β-HSD

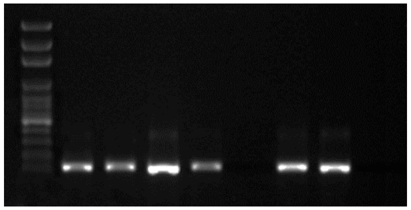

Hsc17β-HSD

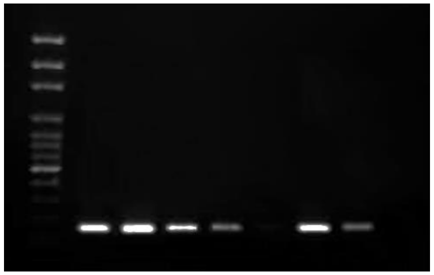

16S rRNA

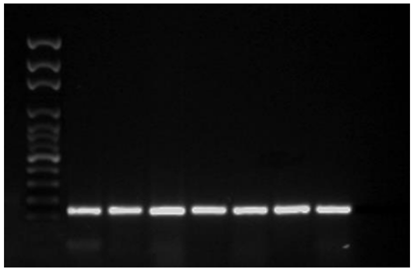

Supplement: Supplementary file 2 — Supplementary Information 1. [file 41598_2021_81917_MOESM2_ESM.pdf]
